# Coupled Rotary Motion in Molecular Motors

**DOI:** 10.1021/jacs.3c14430

**Published:** 2024-02-13

**Authors:** Carlijn L. F. van Beek, Ben L. Feringa

**Affiliations:** Stratingh Institute for Chemistry, Faculty of Science and Engineering, University of Groningen, Nijenborgh 4, Groningen, 9747 AG, Netherlands

## Abstract

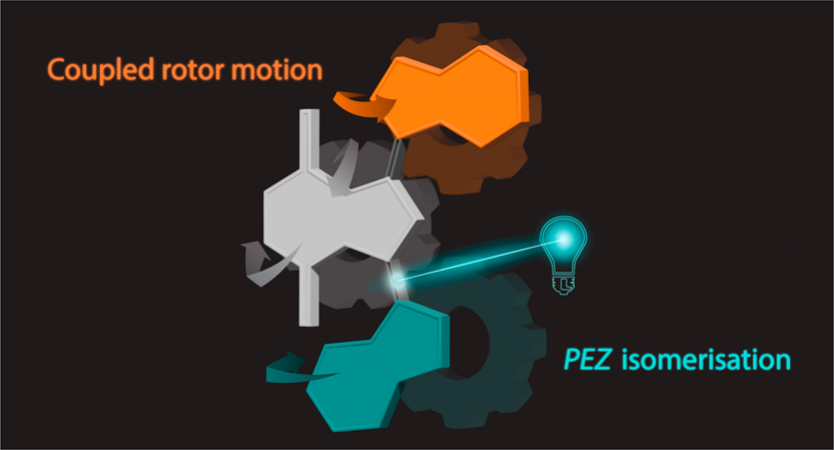

Biological molecular machines play a pivotal role in sustaining life by producing a controlled and directional motion. Artificial molecular machines aim to mimic this motion, to exploit and tune the nanoscale produced motion to power dynamic molecular systems. The precise control, transfer, and amplification of the molecular-level motion is crucial to harness the potential of synthetic molecular motors. It is intriguing to establish how directional motor rotation can be utilized to drive secondary motions in other subunits of a multicomponent molecular machine. The challenge to design sophisticated synthetic machines involving multiple motorized elements presents fascinating opportunities for achieving unprecedented functions, but these remain almost unexplored due to their extremely intricate behavior. Here we show intrinsic coupled rotary motion in light-driven overcrowded-alkene based molecular motors. Thus far, molecular motors with two rotors have been understood to undergo independent rotation of each subunit. The new bridged-isoindigo motor design revealed an additional dimension to the motor’s unidirectional operation mechanism where communication between the rotors occurs. An unprecedented double metastable state intermediate bridges the rotation cycles of the two rotor subunits. Our findings demonstrate how neighboring motorized subunits can affect each other and thereby drastically change the motor’s functioning. Controlling the embedded entanglement of active intramolecular components sets the stage for more advanced artificial molecular machines.

## Introduction

The exquisite function and motion arising from biological nanoscale machinery inspired the development of artificial stimuli-responsive molecular motors and machines.^1−10^ Translating molecular-level motion to achieve useful responsive behavior and dynamic functions at a macroscopic level by performing collective action with molecular machines is mastered by biomolecular systems, but despite major developments, it remains challenging to accomplish with artificial systems.^10−16^ Understanding and controlling the cooperative action of intramolecular, instead of intermolecular, motor components presents another important fundamental challenge to be addressed to establish truly multifunctional synthetic machines.^17^ Recent advances regarding transmitting unidirectional rotational motion to secondary passive components such as coupled geared motion, threading and winding, present the first steps toward unraveling these intricate intramolecular dynamics.^18−25^ However, advanced synthetic molecular machines will likely contain several active motorized entities and their increased complex behavior is barely explored.^26,27^

Here, we present the fundamental principle of coupled rotary motion in light-driven overcrowded-alkene-based molecular motors. In our novel design of bridged-isoindigo molecular motors, this unique coupling of the rotation cycles of individual rotor units demonstrates the profound impact that active motorized subcomponents can have on each other in a molecular machine. Furthermore, we identified for the first time eight distinct species involved in a full 360° rotary cycle and observed an unprecedented double metastable isomer involved in the rotary process.

**Figure 1:**
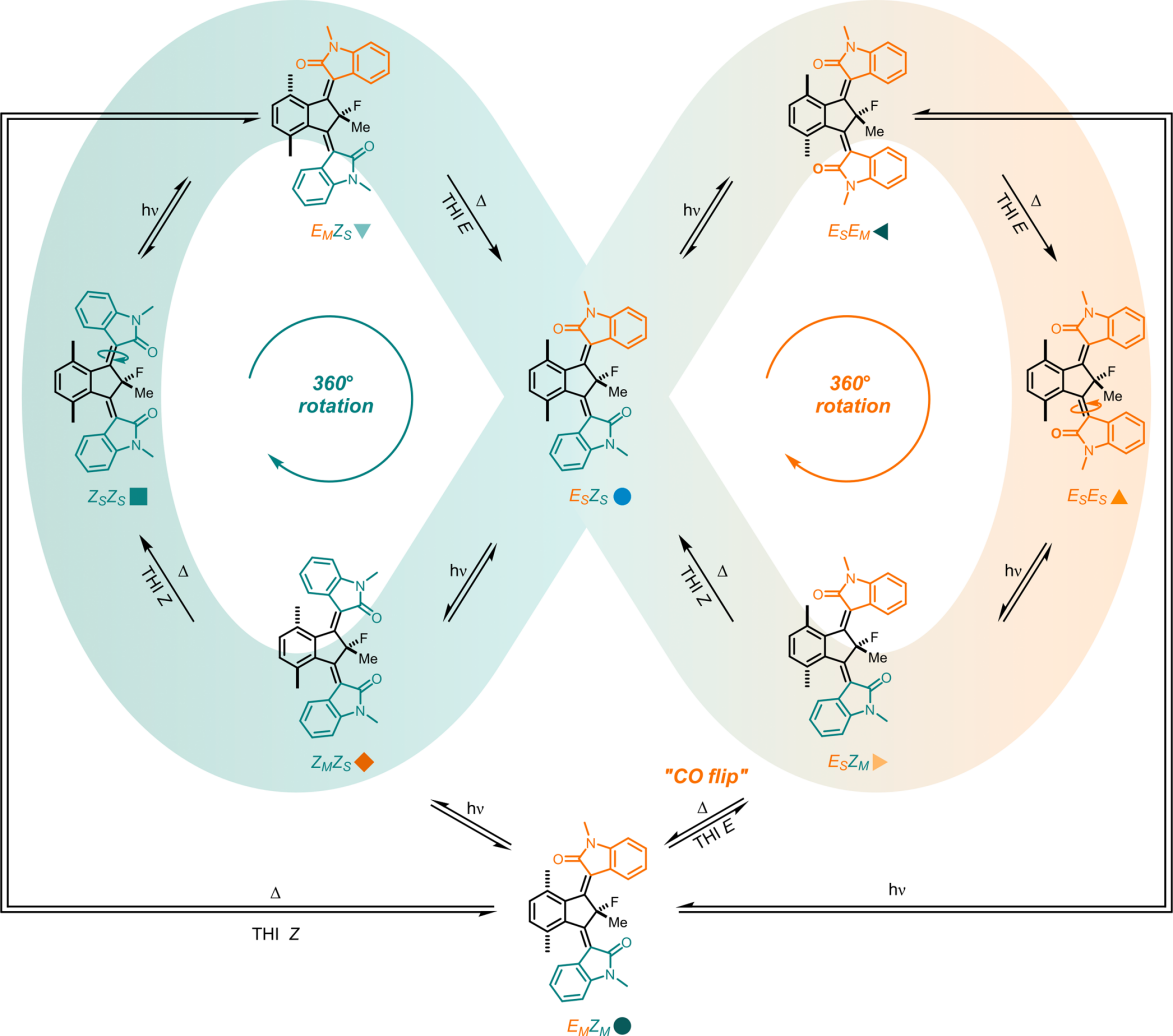
Rotational cycle of bridged-isoindigo molecular motor **1**. Unidirectional rotational cycle of **1** consisting of photochemical *E/Z* (PEZ) isomerization and thermal helix inversion (THI) processes, which connect the three dimensions of intermediates (stable, single metastable, and double metastable states). The stable-state isomers (*Z*_S_*Z*_S_, *E*_S_*Z*_S_, and *E*_S_*E*_S_) are located in the in-loop middle, the single metastable-state isomers (*Z*_M_*Z*_S_, *E*_M_*Z*_S_, *E*_S_*Z*_M_, and *E*_S_*E*_M_) are located in the in-loop top and bottom, and the double metastable-state isomer (*E*_M_*Z*_M_) is located in the off-loop bottom. PEZ isomerization converts a stable state rotor to a metastable configuration. During a THI, a rotor moves to the opposite side of the central core (helical inversion), which transforms the rotor’s configuration from metastable to stable. The off-loop steps indicate previously unknown mechanistic pathways.

Unidirectional rotary motion of light-driven molecular motors is governed by a photochemical *E/Z* (PEZ) isomerization and subsequent thermal helix inversion (THI) between the stable states of the motor (Figure 1).^28,29^ The asymmetry of the two rotors gives rise to four different stable isomers of the resulting molecular motor, for example for motor **1**: (*Z*_S_*Z*_S_)-**1**, (*E*_S_*Z*_S_)-**1**, (*Z*_S_*E*_S_)-**1**, and (*E*_S_*E*_S_)-**1** (the subscript label represents the stable (S) or metastable geometry (M) of the associated rotor). The rotational cycles of (*E*_S_*Z*_S_)-**1** and (*Z*_S_*E*_S_)-**1** are identical but mirror images due to their enantiomeric relationship and overlap at the meso isomers (*Z*_S_*Z*_S_)-**1** and (*E*_S_*E*_S_)-**1**. This reflects the equal probability of activating each of the two rotors of (*Z*_S_*Z*_S_)-**1** or (*E*_S_*E*_S_)-**1**. For clarity, we only display one isomer of the enantiomeric pair ((*E*_S_*Z*_S_)-**1**) in the mechanistic overview of the rotary process (Figure 1). For third generation molecular motors, irradiation with light causes one of the two rotors to isomerize, thereby generating a single metastable state (e.g., isomerization of *Z*_S_*Z*_S_ to *E*_M_*Z*_S_). The produced metastable state releases the build-up strain via a ratcheting THI step to form the corresponding stable isomer (e.g., relaxation of *E*_M_*Z*_S_ to *E*_S_*Z*_S_). Our previously proposed rotational cycle of third-generation molecular motors consists of four unique PEZ isomerization–THI sequences connecting the three stable isomers (*Z*_S_*Z*_S_*, E*_S_*Z*_S_, and *E*_S_*E*_S_ (Figure 1, middle)) through four distinct single metastable states (displayed by the in-loop steps in Figure 1).^30,31^ The unidirectional rotary motion is ensured by the helicity of each rotor induced by the pseudoasymmetric center bearing a fluorine and methyl substituent of distinct size. The rotation direction of the two rotors is inherently coupled because of the motor design with each rotor connected through a C–C double bond, the rotation axis, on opposite sides of the central core bearing the pseudoasymmetric center. The individual rotors experience opposite helicities and together thus produce a disrotary motion. The rotor rotation can be compared to the directional rotation of two wheels on an axis. From the perspective of the car driver, who is positioned at the symmetry plane, the wheels rotate in opposite directions (one rotates clockwise and the other counterclockwise).

Based on our previous studies employing fluorene-based rotors,^30,31^ the rotation cycle of each rotor was understood to be independent of the other rotor, such that a rotor completes a full PEZ isomerization–THI sequence (which produces a unidirectional 180° rotation) before the same or the other rotor can be activated. However, the present study shows that sequential rotation of the rotor units upon light irradiation is not necessarily the motor’s mode of action. Unprecedented coupled rotary motion was found for motor **1**, which demonstrates for the first time the involvement of a molecular motor isomer with both rotors in a double metastable-state geometry in the rotation cycle. The unique observation of this coupled motion at the nanoscale featuring a double metastable-state isomer (*E*_M_*Z*_M_)-**1** (Figure 1, bottom) is a key consequence of the influence that the proximal rotor units have on each other. This rotor–rotor influence (represented by the off-loop pathways in Figure 1) further demonstrates the complexity of the rotational behavior of third-generation motors without compromising the unidirectionality.

## Results and Discussion

**Figure 2:**
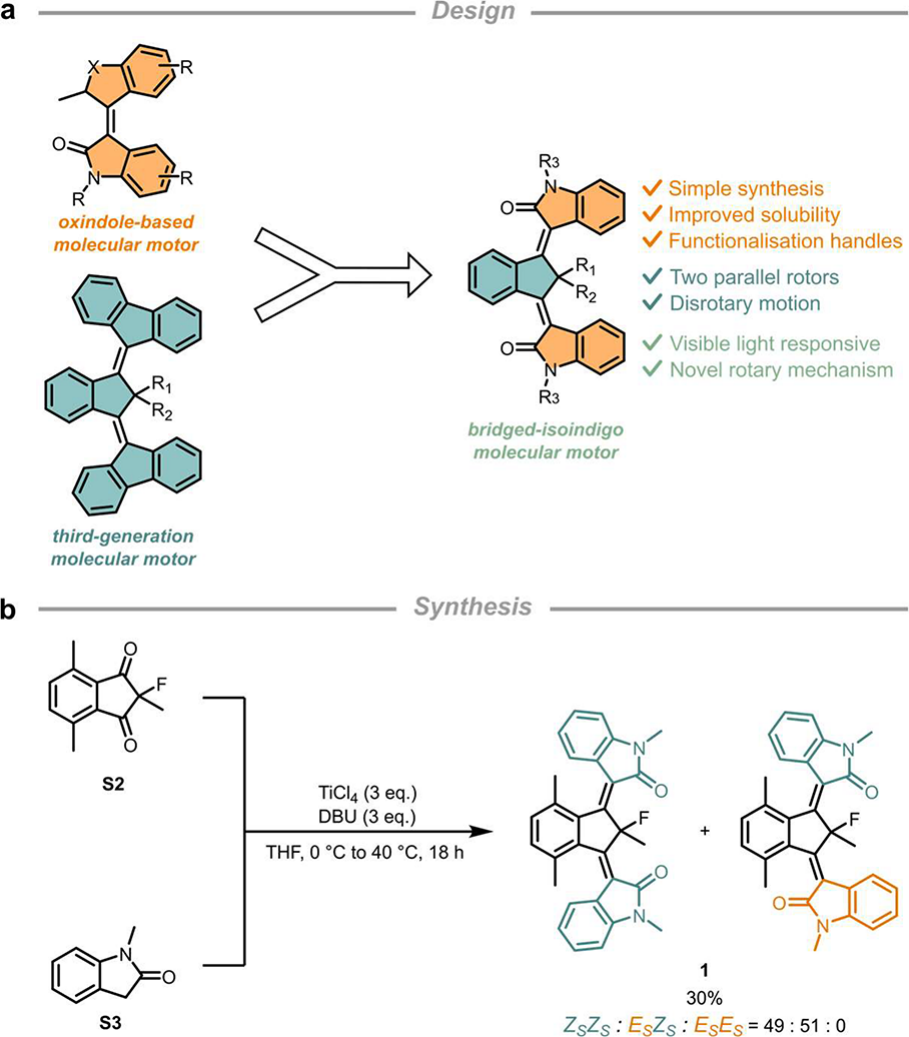
Design and synthesis of bridged-isoindigo molecular motors. (a) Introducing functionalized rotors by merging third-generation molecular motors with oxindole-based molecular motors. (b) One-pot double Knoevenagel condensation to synthesize bridged-isoindigo molecular motor **1**.

Our motor design is a heteroatom analogue of third-generation molecular motors^30−32^ in which the central core bridges two oxindole-based rotors (Figure 2a). The visible-light-driven disrotary motion of two parallel rotors, which is a key third-generation molecular motor property, is maintained in the new bridged-isoindigo molecular motor design. Bridged-isoindigo molecular motors overcome several limitations of third-generation molecular motors. The use of oxindole rotors not only circumvents the tedious conventional synthesis of traditional third-generation molecular motors based on a Barton-Kellogg coupling but also greatly enhances the solubility of the motor in common organic solvents and provides additional handles for functionalization. Apart from addressing these practical aspects, the bridged-isoindigo molecular motor also allowed for an in-depth mechanistic study of the rotor motions, which was previously not possible due to overlapping signals for motors with fluorene-based rotors. Most importantly, this enabled us to discover unprecedented coupled rotary motion in light-powered motors.

**Figure 3:**
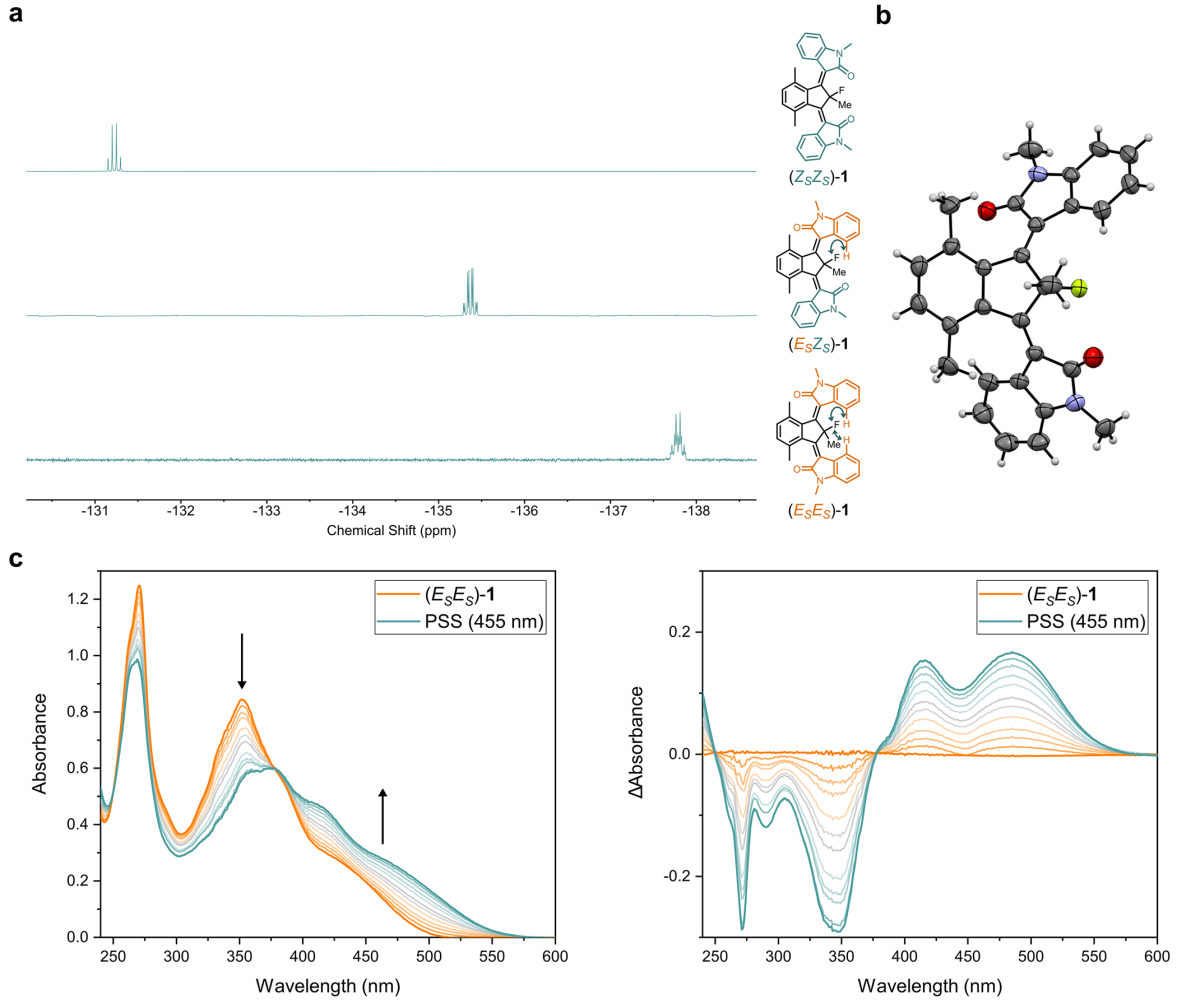
Characterization and photochemical isomerization of motor **1**. (a) ^19^F NMR spectra (376 MHz, 20 °C, CD_2_Cl_2_) of (*Z*_S_*Z*_S_)-**1**, (*E*_S_*Z*_S_)-**1**, and (*E*_S_*E*_S_)-**1**. The arrows indicate through-space ^1^H–^19^F coupling interactions. (b) X-ray structure of (*E*_*S*_*Z*_*S*_)-**1**. Ellipsoids are set at the 50% probability. (c) UV/vis absorption spectra (left) and difference spectra (right) of (*E*_S_*E*_S_)-**1** in CH_2_Cl_2_ (4 × 10^–5^ M, −90 °C) upon irradiation with 455 nm light. The pure isomer (*E*_S_*E*_S_)-**1** is shown in orange and the obtained PSS containing (*E*_S_*E*_S_)-**1** and (*E*_S_*Z*_M_)-**1** in blue. An isosbestic point is maintained at 378 nm.

Molecular motor **1** was readily prepared using a newly developed single-step double Knoevenagel condensation (see Figure 2b and the Supporting Information) inspired by our work on simple oxindole-based molecular motors.^33,34^ The stable isomers ((*Z*_S_*Z*_S_)-**1**, (*E*_S_*Z*_S_)-**1**, and (*E*_S_*E*_S_)-**1**) were easily distinguishable using ^19^F NMR spectroscopy due to their characteristic chemical shifts as well as splitting patterns. Through-space coupling of the fluorine atom with proximal aromatic protons was previously observed for fluorene-based molecular motors,^30^ but the dissymmetry of the oxindole rotor units provides additional information for differentiating its *E* and *Z* isomers (Figure 3a). In our motor design, the proton-fluorine through-space coupling (*J* = 5.4–5.7 Hz) occurs only for rotors in the *E* configuration.^35^ The assignment of the stable state isomers was consistent with our additional one-dimensional (1D) and two-dimensional (2D) nuclear magnetic resonance (NMR) data. Single-crystal X-ray analysis of (*Z*_S_*Z*_S_)-**1** and (*E*_S_*Z*_S_)-**1** provided unequivocal proof for the structure of motor **1** (Figure 3b and Figure S29 and S30).

The rotary behavior of motor **1** was investigated in-depth using UV/vis absorption and NMR irradiation studies. Irradiation of (*E*_*S*_*E*_*S*_)-**1** at −90 °C with visible light (455 nm) resulted in a bathochromic shift in the UV/vis absorption spectrum with an isosbestic point at 378 nm, which is consistent with the formation of (*E*_S_*Z*_M_)-**1** (Figures 1 and 3c, bottom right). Starting instead from (*Z*_S_*Z*_S_)-**1** or (*E*_S_*Z*_S_)-**1** gives an identical photostationary state (PSS) to that initiated from (*E*_S_*E*_S_)-**1** but without maintaining isosbestic points (Figures S4 and S5). The lack of isosbestic points is attributed to concurrent transformations of the formed metastable states ((*E*_M_*Z*_S_)-**1** and (*E*_S_*E*_M_)-**1**, respectively) due to the relatively low THI_*E*_ activation barriers. Lowering the temperature to −110 °C enabled us to study the photochemical isomerization from (*Z*_S_*Z*_S_)-**1** or (*E*_S_*Z*_S_)-**1** to their respective metastable state by avoiding the associated THI_*E*_ processes (Figures S7–S10). The emergence of two different isosbestic points for the individual photochemical and thermal steps reveals a sequence-specific conversion from one stable state isomer to a different stable state isomer (e.g., (*Z*_S_*Z*_S_)-**1** → (*E*_M_*Z*_S_)-**1** → (*E*_S_*Z*_S_)-**1**; see Figure 1, top left) as expected from the proposed unidirectional rotation mechanism. The determined activation barriers for the THI_*E*_ relaxations were very similar for (*E*_M_*Z*_S_)-**1** to (*E*_S_*Z*_S_)-**1** (Δ^‡^*G*°_THI-*E*_ (−110 °C, Et_2_O) = 12.0 kcal mol^–1^) and (*E*_S_*E*_M_)-**1** to (*E*_S_*E*_S_)-**1** (Δ^‡^*G*°_THI-*E*_ (−110 °C, Et_2_O) = 11.8 kcal mol^–1^). To our surprise, PEZ isomerization of (*E*_S_*Z*_S_)-**1** is strongly biased toward (*E*_S_*E*_M_)-**1** and hence results in the minimal formation of (*Z*_M_*Z*_S_)-**1**. The asymmetry of the (*E*_S_*Z*_S_)-**1** photoexcitation is of particular interest as it could potentially offer selectivity for *E/Z* rotor activation. Modulation of the PSS ratio is possible using different irradiation wavelengths, but in no case considerable formation of (*Z*_M_*Z*_S_)-**1** was observed. The strong bias of (*E*_S_*Z*_S_)-**1** for activation of the *Z* rotor producing (*E*_S_*E*_S_)-**1** (via (*E*_S_*E*_M_)-**1**) thus precludes selective rotor activation of (*E*_S_*Z*_S_)-**1** to favor the generation of (*Z*_M_*Z*_S_)-**1** (see the Supporting Information, page 26, for a detailed discussion). Switching between (*E*_S_*E*_S_)-**1** and (*E*_S_*Z*_M_)-**1** using 455 and 530 nm light alternately was performed without any observable decomposition over three switching cycles, demonstrating the robustness of motor **1** (Figure S12).

**Figure 4:**
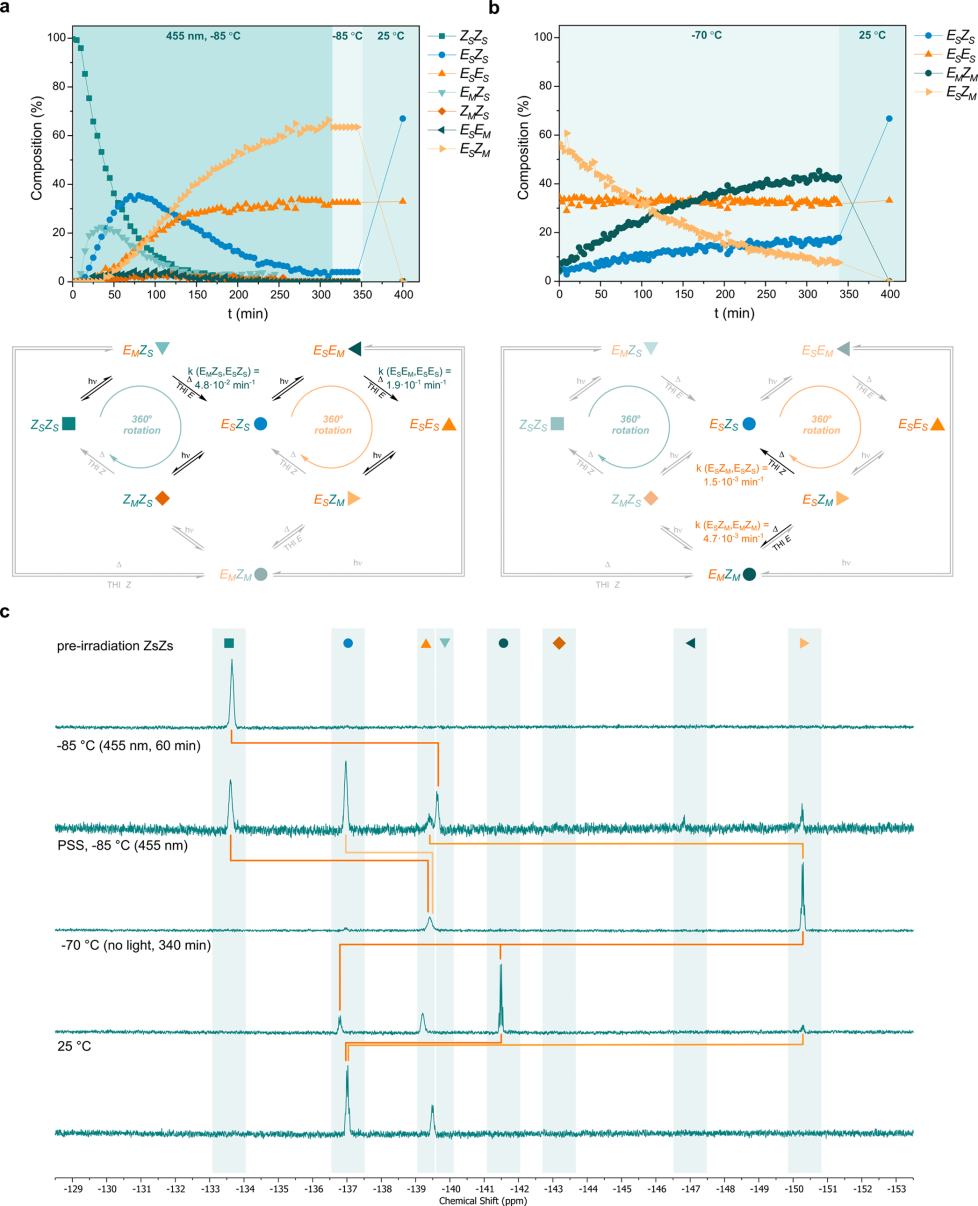
Photochemical and thermal isomerization of motor **1**.(a) Sample of (*Z*_S_*Z*_S_)-**1** irradiated to PSS with 455 nm light at −85 °C. The sample was subsequently kept at −85 °C for 45 min and warmed up to room temperature. The proposed mechanism with determined rate constants and occurring processes highlighted (bottom). (b) Thermal decay of pre-irradiated (*Z*_S_*Z*_S_)-**1** (PSS with 455 nm light at −85 °C) at −70 °C. The sample was subsequently warmed up to room temperature. Proposed mechanism with determined rate constants and occurring processes highlighted (bottom). (c) Corresponding ^19^F NMR spectra (470 MHz, 5 mM in CD_2_Cl_2_) of (*Z*_S_*Z*_S_)-**1** upon irradiation and after partial and full relaxation.

To gain further insight in the complex rotary behavior, we followed the compositional changes upon irradiation and relaxation of motor **1** using ^19^F NMR spectroscopy, which enabled the differentiation and unprecedented characterization of all eight isomers (Figure 4c; see the Supporting Information for complementary ^1^H NMR data). Irradiation of (*Z*_S_*Z*_S_)-**1** with 455 nm light at −85 °C resulted in the appearance of six additional upfield ^19^F shifts showing a stepwise and sequence-specific kinetic profile (Figure 4a). The isomerization from (*Z*_S_*Z*_S_)-**1** to (*E*_M_*Z*_S_)-**1** is followed by the formation of (*E*_S_*Z*_S_)-**1**, indicating that the consecutive THI_*E*_ occurred. When (*E*_S_*Z*_S_)-**1** is the main component of the sample, the PEZ isomerization–THI sequence from (*E*_S_*Z*_S_)-**1** to (*E*_S_*E*_S_)-**1** becomes dominant until the PSS comprised of primarily (*E*_S_*Z*_M_)-**1** and (*E*_S_*E*_S_)-**1** ((*E*_S_*Z*_M_)-**1**:(*E*_S_*E*_S_)-**1** = 65:35) is established. The composition of the PSS mixture remained unchanged in the dark confirming the anticipated stability of (*E*_S_*Z*_M_)-**1** at −85 °C. Repeating the irradiation experiments using another starting stable isomer generated the same PSS in accordance with the behavior observed by UV/vis absorption spectroscopy (vide supra).

Warming the PSS mixture to room temperature in the dark induced the relaxation of (*E*_S_*Z*_M_)-**1** to (*E*_S_*Z*_S_)-**1**. However, relaxation of the PSS mixture at −70 °C in the dark revealed the involvement of two thermal processes (Figure 4b). The direct conversion of (*E*_S_*Z*_M_)-**1** to (*E*_S_*Z*_S_)-**1** via a THI_*Z*_ is outcompeted by a CO flip forming mainly (*E*_M_*Z*_M_)-**1** instead of (*E*_S_*Z*_S_)-**1**. Subsequent full relaxation at room temperature yielded the same composition of isomers as direct complete relaxation from the PSS, and thus a thermal pathway connecting (*E*_M_*Z*_M_)-**1** and (*E*_S_*Z*_S_)-**1** must exist. The involved thermal processes were quantified by determination of the rate constants from the kinetic data. The activation barriers of THI_*E*_ in CD_2_Cl_2_ at −85 °C (Δ^‡^*G*°_THI-*E*_ = 13.5 kcal mol^–1^ for THI_*E*_(*E*_M_*Z*_S_*, E*_S_*Z*_S_) and Δ^‡^*G*°_THI-*E*_ = 13.0 kcal mol^–1^ for THI_*E*_(*E*_M_*E*_S_*, E*_S_*E*_S_)) are again comparable to each other, indicating that these THI_*E*_ steps are minimally affected by the stable configuration of the second rotor. The fact that the THI_*E*_ barriers based on the NMR data are slightly higher than those determined in Et_2_O at −110 °C is presumably due to a difference in viscosity (for further discussion, see the Supporting Information, page 28). The CO flip, formally a THI_*E*_ from (*E*_S_*Z*_M_)-**1** to (*E*_M_*Z*_M_)-**1**, has a significantly higher activation barrier of 15.6 kcal mol^–1^ (−85 °C, CD_2_Cl_2_) than those determined for the THI_*E*_ processes from single metastable states to stable states. The metastable configuration of the *Z* rotor thus clearly impacts the CO flip, whereas the stable configurations of the adjacent rotor do not. As a result, the CO flip barrier of activation is only slightly lower than that for the THI_*Z*_ from (*E*_S_*Z*_M_)-**1** to (*E*_S_*Z*_S_)-**1** (Δ^‡^*G*°_THI-*Z*_ (−85 °C, CD_2_Cl_2_) = 16.0 kcal mol^–1^). In theory, two successive PEZ isomerization steps could induce isomerization of both rotors to produce double metastable-state species (e.g., the photochemical formation of (*E*_M_*Z*_M_)-**1**). Photoexcitation of a single metastable state to a double metastable state was however never observed even upon extended irradiation, which is consistent with previous studies.^30^ Independent NMR experiments were performed to substantiate the novel pathway and provided additional kinetic parameters (Supporting Information).

For motor **1**, the photochemical isomerization of one rotor thus induces a conformational change in the other rotor instead of leading to a THI of the isomerized rotor. PEZ isomerization from (*E*_S_*E*_S_)-**1** to (*E*_S_*Z*_M_)-**1** is mainly followed by a thermodynamically downhill CO flip to generate (*E*_M_*Z*_M_)-**1**, which outcompetes the traditional THI_*Z*_ from (*E*_S_*Z*_M_)-**1** to (*E*_S_*Z*_S_)-**1** (Figure 1, bottom right). The CO flip involves the movement of the carbonyl group of the *E* rotor to the other side of the core unit, which changes the helicity and was therefore identified as an alternative THI relaxation pathway of (*E*_S_*Z*_M_)-**1**. While the abovementioned PEZ is part of the rotation cycle of the lower rotor, the CO flip involves motion of the upper rotor. This remarkable observation implies that the rotation cycles of each individual rotor are bridged by the (*E*_M_*Z*_M_)-**1** intermediate and should not be considered independently. The discovery of this intrinsic coupled motion reveals that the rotary motions of the two light-driven rotors are indeed mutually dependent.

The formation of double metastable states is highly dependent on the relative barriers of the two competing THI pathways. During a THI_*E*_, a carbonyl group moves past the methyl groups on the core, whereas a THI_*Z*_ requires sliding of a substantially larger aryl moiety past the bulky core. Relaxation via a CO flip is hence strongly kinetically favored for (*E*_S_*Z*_M_)-**1** because of the considerably lower activation barrier for the CO flip (a THI_*E*_) compared to that for the THI_*Z*_. For the same reason, accessing (*E*_M_*Z*_M_)-**1** from (*E*_M_*Z*_S_)-**1** instead of (*E*_S_*Z*_M_)-**1** requires a THI_*Z*_ to outcompete a THI_*E*_. This THI_*Z*_ pathway is a negligible process based on kinetic considerations. Apart from thermal steps, photochemical processes from (*Z*_M_*Z*_S_)-**1** and (*E*_S_*E*_M_)-**1** provide additional pathways to (*E*_M_*Z*_M_)-**1** (Figure 1, off-loop right and left). (*E*_M_*Z*_M_)-**1** presents a new dimension in the motor operation mechanism that is accessible only via the connected single metastable states. The two other conceivable double metastable state isomers ((*Z*_M_*Z*_M_)-**1** and (*E*_M_*E*_M_)-**1**) were never detected in line with our computational study (vide infra) and hence will not be further considered.^36^

**Figure 5:**
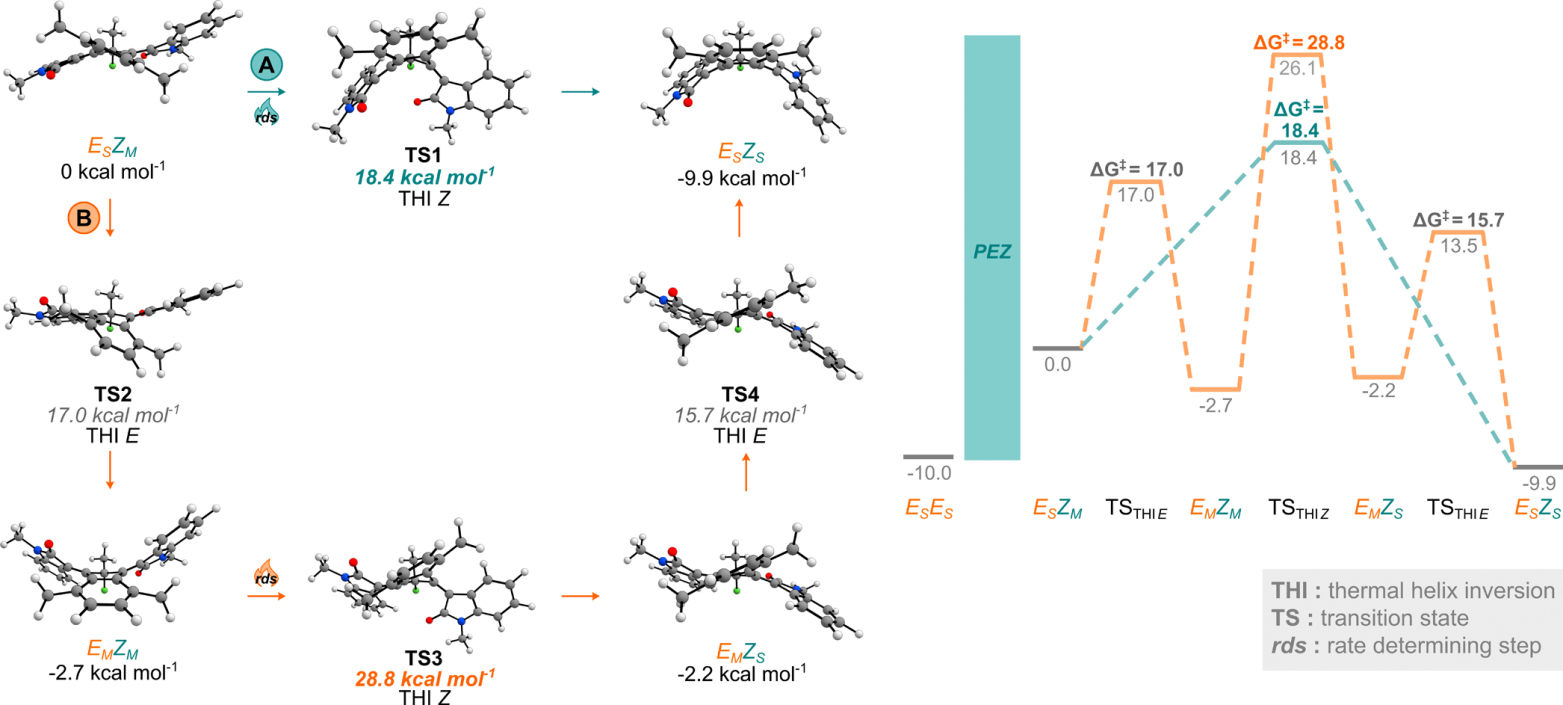
Thermal isomerization processes of (*E*_M_*Z*_M_)-**1**. Calculated thermal conversion of (*E*_S_*Z*_M_)-**1** to (*E*_S_*Z*_S_)-**1** via pathways A (THI_*Z*_ shown in orange) and B (a series of CO flip (THI_*E*_), THI_*Z*_, and THI_*E*_ shown in blue). The energies are referenced to (*E*_S_*Z*_M_)-**1**. For transition states, the activation barriers are relative to the associated initial state. Energies and activation barriers were calculated at the r^2^SCAN-3c CPCM(CH_2_Cl_2_)) level of theory and are shown in kcal mol^–1^.

Computational analysis of motor **1** also clearly presented a dependency of THI activation barriers on adjacent metastable rotors but not on stable rotors (see Section S6 for computational details). The calculated THI_*E*_ barriers of single metastable states appeared unaffected by the stable rotor conformation (15.4–15.7 kcal mol^–1^), whereas the introduction of a metastable rotor led to increased THI_*E*_ barriers (17.0–18.3 kcal mol^–1^). This rotor influence extended to the THI_*Z*_ barriers, which showed an even more pronounced rotor dependency. Double metastable states possess eminently higher THI_*Z*_ barriers (up to 10 kcal mol^–1^) than their single metastable state counterparts. The calculated activation barriers were generally slightly higher than those determined experimentally, but the trends derived from the differences in activation barriers matched closely. Comparison of the calculated relaxation pathways of (*E*_S_*Z*_M_)-**1** to (*E*_S_*Z*_S_)-**1** indeed revealed a kinetic preference for partial relaxation to (*E*_M_*Z*_M_)-**1** via a CO flip (pathway B) over a direct THI_*Z*_ to (*E*_S_*Z*_S_)-**1** (pathway A) (Figure 5). The relatively high THI_*Z*_ barrier associated with (*E*_M_*Z*_M_)-**1** advancing to (*E*_M_*Z*_S_)-**1** causes full relaxation from (*E*_M_*Z*_M_)-**1** to (*E*_S_*Z*_S_)-**1** to essentially proceed exclusively via the sequence of reverse CO-flip and THI_*Z*_ ((*E*_M_*Z*_M_)-**1** → (*E*_S_*Z*_M_)-**1** → (*E*_S_*Z*_S_)-**1**). As expected based on the experimental data, our calculations confirmed that the double metastable states are energetically between their stable and single metastable states. The origin of the unusual stability of double metastable states remains unknown, but possibly, the stabilization arises from their folded structure. Single metastable states adopt twisted geometries with the rotors at opposite sides of the motor core, while the lower energy stable states have more symmetric structures with rotors folded toward the fluorine atom (see Figure 5 and Figure S32). The inverted folded structure of double metastable states with both rotors folded toward the methyl group at the pseudo asymmetric center resembles the folded stable states.

**Figure 6:**
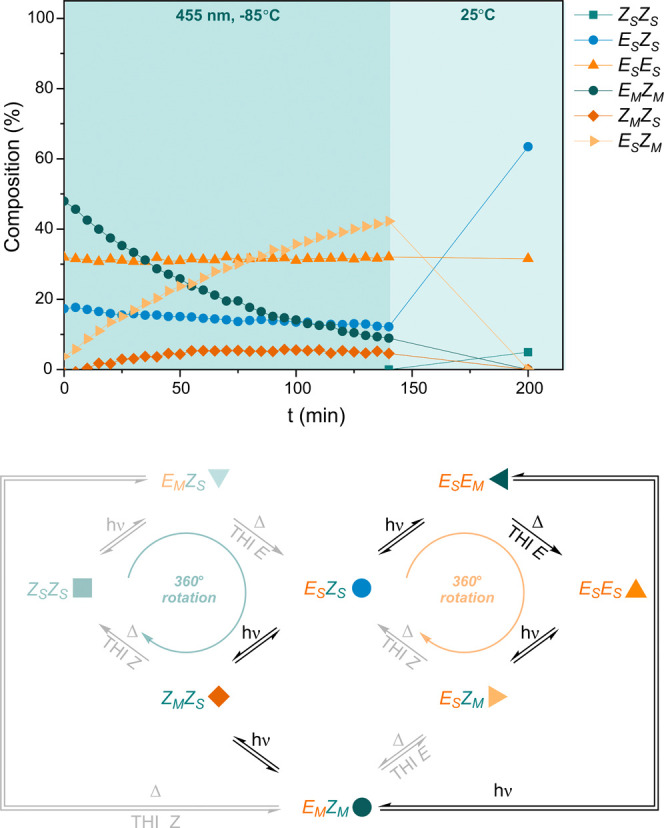
Photochemical isomerization processes of (*E*_M_*Z*_M_)-**1**. A sample enriched with (*E*_M_*Z*_M_)-**1** irradiated with 455 nm light for 140 min at −85 °C, in the dark for 10 min at −85 °C and subsequently warmed up to room temperature. The kinetic traces were followed using ^19^F NMR spectroscopy (470 MHz, 11 mM in CD_2_Cl_2_) (top). Motor operation overview under the illumination of (*E*_M_*Z*_M_)-**1** at −85 °C (bottom). The enrichment of the sample with (*E*_M_*Z*_M_)-**1** was achieved by thermal relaxation of the pre-irradiated PSS mixture (with 455 nm light at −85 °C) at −55 °C.

Next, the photochemical isomerization behavior of (*E*_M_*Z*_M_)-**1** was studied by in situ NMR irradiation at a low temperature to preclude THI_*Z*_ and CO-flip transformations. Irradiation with 455 nm light effectively isomerized (*E*_M_*Z*_M_)-**1**, thereby forming (*Z*_M_*Z*_S_)-**1** and instigating the repopulation of (*E*_S_*Z*_M_)-**1** (Figure 6). Interestingly, the illumination of (*E*_M_*Z*_M_)-**1** proved to be the most efficient way to substantially populate (*Z*_M_*Z*_S_)-**1**. No direct photochemical equilibrium between (*E*_M_*Z*_M_)-**1** and (*E*_S_*Z*_M_)-**1** exists and their thermal interconversion (CO flip) is inaccessible at −85 °C. The rapid repopulation of (*E*_S_*Z*_M_)-**1** can instead be explained by a series of photochemical and thermal steps. (*E*_S_*E*_M_)-**1** is photochemically formed from (*E*_M_*Z*_M_)-**1** either directly or indirectly via (*Z*_M_*Z*_S_)-**1** and (*E*_S_*Z*_S_)-**1** intermediates. The minimal buildup of (*E*_S_*E*_M_)-**1** despite the strong photochemical bias toward this isomer (vide supra) is attributed to the facile THI_*E*_ to (*E*_S_*E*_S_)-**1** at −85 °C. The determined activation barrier of THI_*E*_(*E*_S_*E*_M_*, E*_S_*E*_S_) is in good agreement with the other kinetic data (12.8 kcal mol^–1^ vs 13.0 kcal mol^–1^ both in CD_2_Cl_2_ at −85 °C). Subsequent photoisomerization of (*E*_S_*E*_S_)-**1** to (*E*_S_*Z*_M_)-**1** re-establishes the PSS for 455 nm light at −85 °C. Notably, the observed kinetic profile provides the first evidence for two sequential PEZ isomerization steps of a light-driven molecular motor, namely, (*E*_M_*Z*_M_)-**1** to (*Z*_M_*Z*_S_)-**1** to (*E*_S_*Z*_S_)-**1** and (*E*_M_*Z*_M_)-**1** to (*E*_S_*E*_M_)-**1** to (*E*_S_*Z*_S_)-**1**. These connected photoequilibria appear to be strongly biased to the stable state or the single metastable state, which might explain the unobserved photoexcitation of a stable state to a double metastable state.

## Conclusions

This study introduces coupled rotary motion of light-driven molecular motors and shows how it impacts the motor’s operation. The combined data support the proposed complex rotation mechanism of motor **1** with uncompromised unidirectionality. The involvement of a double metastable state intermediate is key for overcoming a rotational loop consisting of (*E*_S_*Z*_S_)-**1**, (*E*_S_*E*_S_)-**1**, and (*Z*_S_*E*_S_)-**1**. Photoisomerization of (*E*_M_*Z*_M_)-**1** replenishes the rather isolated *Z*_S_*Z*_S_/*E*_S_*Z*_S_ rotational cycle, thereby achieving a more efficient and effective motor operation. We envision that this new dimension to molecular motors operation will be crucial for understanding and designing future sophisticated systems comprising multiple motorized units functioning cooperatively. The entangled motion of intramolecular rotor units is an important fundamental aspect to consider in future molecular machinery designs and offers a completely unexplored tool to control and optimize the precise motor functioning.

## Supporting Information Available

Experimental procedures with characterizations, photochemical and thermal studies with UV/vis absorption and NMR data and associated kinetic analyses, crystal structures, and computational details (PDF, DOC)
XYZ Files of the computational study (ZIP)
