# Supplementary material for: Coupled Rotary Motion in Molecular Motors
Source: J Am Chem Soc. 2024 Feb 13;146(8):5634–42. doi: 10.1021/jacs.3c14430 (PMC10910502; doi:10.1021/jacs.3c14430)
Supplement: Supplementary file 1 — ja3c14430_si_001.pdf [file ja3c14430_si_001.pdf]

Supporting Information

Coupled rotary motion in molecular motors

Carlijn L. F. van Beek, Ben L. Feringa*

Stratingh Institute for Chemistry, Faculty of Science and Engineering,
University of Groningen, Nijenborgh 4, Groningen 9747 AG, The Netherlands

* Corresponding author. E-mail: b.l.feringa@rug.nl

Contents

1. General Information 3
2. Synthetic Procedures 4
3. UV/Vis Absorption Spectra 8
4. NMR Studies and Kinetic Experiments 18

NMR Irradiation Experiments 18

Room temperature experiments 18

Low temperature experiments 20

Investigation of selective rotor activation 26

Kinetic Analysis 28

Relaxation kinetics of E_MZ_S and E_SE_M at -85 °C 29

Relaxation kinetics of E_SZ_M and E_MZ_M at -70 °C 30

Relaxation kinetics of E_SE_M at -85 °C 31

Relaxation kinetics of E_MZ_S at -85 °C 32

Relaxation kinetics of E_MZ_M at -30 °C 33

5. Single Crystal XRD 34
6. Computational Details 38
7. NMR Spectra 42
8. HRMS Spectra 54
9. References 55

1. General Information

All reagents were obtained from commercial sources and used as received without further purification. Dry solvents were obtained from a MBraun solvent purification system. Progress of the reactions was determined by UPLCMS (Waters Acquity Ultra Performance LC system with Acquity UPLC BEH C18, 2.1 x 50 mm 1.7 µm particles column) and TLC: silica gel 60, Merck, 0.25 mm. The TLC plates were visualized with ultraviolet (UV) light (λ = 254 nm or 355 nm). Column chromatography was performed on a Biotage Selekt System. High Resolution Mass Spectra (HMRS) were obtained using an LTQ Orbitrap XL. NMR spectra were recorded on a Varian Mercury Plus (^{1}H: 400 MHz, ^{13}C: 100 MHz, ^{19}F: 376 MHz), an Agilent MR (^{1}H: 400 MHz, ^{19}F: 376 MHz), a Varian Innova (^{1}H: 500 MHz, ^{19}F: 470 MHz) or a Bruker Avance Neo with Cryoprobe Prodigy BBO (^{1}H: 600 MHz, ^{13}C: 150 MHz, ^{19}F: 565 MHz) instrument. Chemical shifts (δ) are in parts per million (ppm) relative to TMS. Chemical shifts are reported in δ-units (ppm) relative to the residual solvent peak of $CDCl_3$ (^{1}H NMR, δ = 7.26 ppm; ^{13}C NMR, δ = 77.16 ppm) or CD_2Cl_2 (^{1}H NMR, δ = 5.32 ppm; ^{13}C NMR, δ = 53.84 ppm). For ^{1}H-NMR spectroscopy, the splitting pattern of peaks is designated as follows: s (singlet), d (doublet), t (triplet), q (quartet), m (multiplet), dd (doublet of doublets), td (triplet of doublets), dq (quartet of doublets), and qt (quartet of triplets). Single-crystal X-ray diffraction measurements were performed on a Bruker-AXS D8 Venture diffractometer. UV/Vis absorption spectra were recorded on a Agilent Cary 8454 spectrophotometer in a 1 cm quartz cuvette. The LEDs were attached via a 1500 µm optical fiber (M93L01). For low temperature (-90 °C or -110 °C) experiments, a Unisoku Cryostat (CoolSpek) was coupled to the spectrophotometer. Solvents used for spectroscopic studies were of spectroscopic grade (UVASOL, Merck). Dichloromethane and Et_2O were degassed with and stored under argon. Irradiation experiments were performed using Thorlabs LEDs (M340F3, M365D2, M395L5, M415L4, M455L4, M530L4). NMR irradiation experiments were performed at the indicated temperature with a fiber-coupled Thorlabs LED and a 1500 µm optical fiber (FT1500UMT) to guide the light into the NMR tube inside the NMR spectrometer. Samples were equilibrated in a pre-cooled Varian Innova 500 spectrometer for 30 min or until the lock signal stabilised.

2. Synthetic Procedures

Synthesis Overview

The core unit was synthesised from *p*-xylene according to modified literature procedures[1]. Methyl malonic acid was converted to 2-methylmalonyl dichloride using thionyl chloride (Figure S1). 2-Methyl malonyl dichloride was immediately used in a double Friedel-Crafts acylation of *p*-xylene to afford bis-ketone **S1**. Fluorination of the acidic position of **S1** with Selectfluor® gave the desired bis-ketone core **S2**.

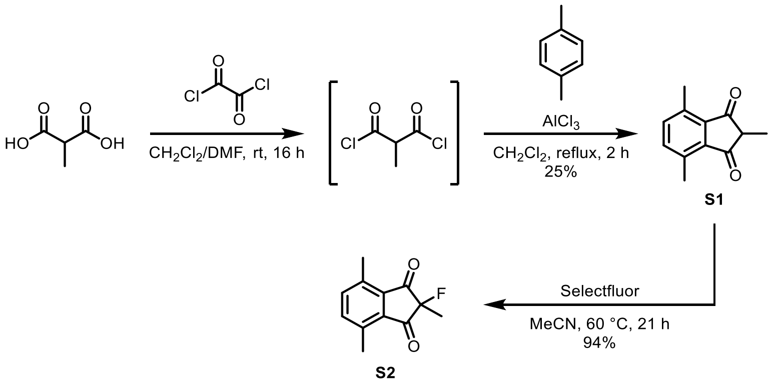

Figure S1. Synthesis of fluorinated motor core **S2**.

A $TiCl_4$-mediated double Knoevenagel condensation of core **S2** with *N*-methyl oxindole **S3** resulted in motor **1** (Figure S2). Motor **1** was only isolated as the stable Z_SZ_S and E_SZ_S isomers. Irradiation of the stable E_SZ_S isomer with 455 nm light to PSS generated a mixture of all three stable isomers, Z_SZ_S, E_SZ_S, and E_SE_S. Separation of the isomers allowed for isolation of the stable E_SE_S isomer.

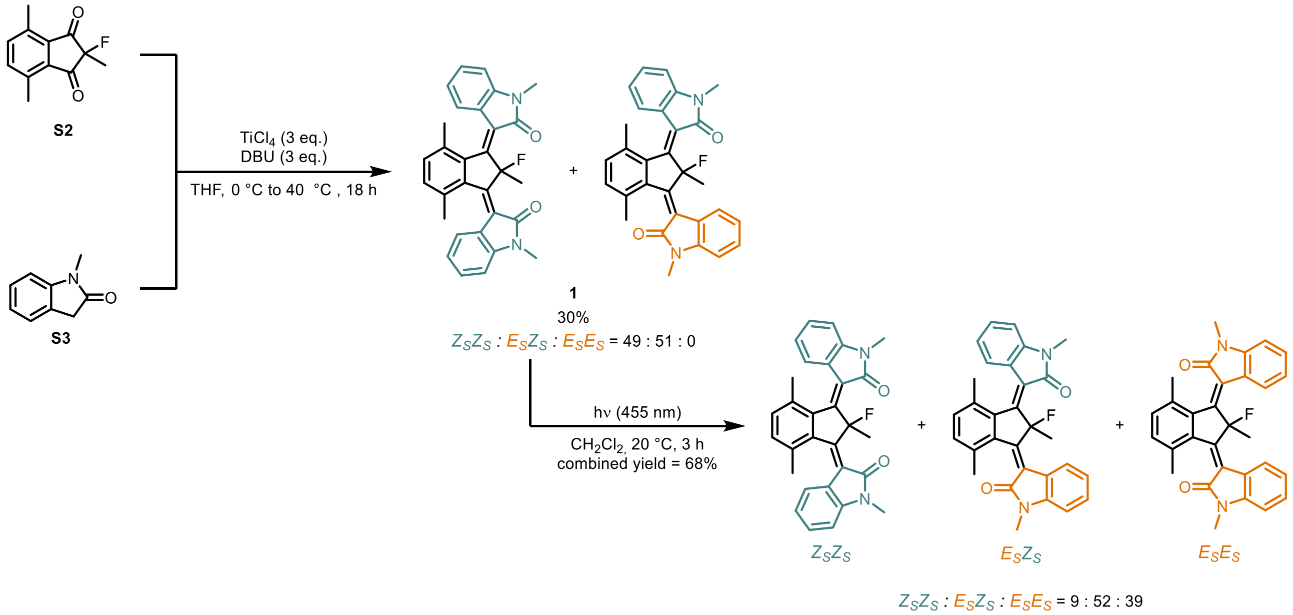

Figure S2. Synthesis of all stable isomers of bridged isoindigo motor **1**.

Synthetic procedures

2,4,7-Trimethyl-1*H*-indene-1,3(2*H*)-dione (S1).[1]

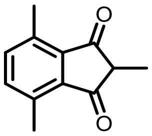

S1

A modified literature procedure[1] was used for the synthesis of **S1**. Oxalyl chloride (25.0 mL, 286 mmol, 4.9 equiv.) was added to a stirred solution of 2-methylmalonic acid (10.0 g, 85.0 mmol, 1.4 equiv.) and few drops of DMF in dry CH_2Cl_2 (150 mL) at room temperature. After stirring for 16 h, the volatiles were removed under reduced pressure. The yellow residue was redissolved in dry CH_2Cl_2 (150 mL) and *p*-xylene (7.3 mL, 59.3 mmol, 1 equiv.) was added. The solution was heated to reflux and solid $AlCl_3$ (23.7 g, 178 mmol, 3.0 equiv.) was added portion wise over 2 h. The reaction was quenched by pouring onto ice (~ 300 g). The organic layer was separated and the water layer was washed with CH_2Cl_2 (2 × 80 mL). The combined organic layers were washed with brine (100 mL) and dried over anhydrous $MgSO_4$. The solvents were evaporated at reduced pressure. The residue was purified by column chromatography (SiO_2, pentane/EtOAc 100:0 to 97:3) followed by two recrystallizations from heptane to give white needles (2.44 g, 12.9 mmol, 22%). Spectroscopic data according to literature[1].

^{1}H NMR (400 MHz, $CDCl_3$) δ 7.43 (s, 2H), 2.99 (q, *J* = 7.7 Hz, 1H), 2.70 (s, 6H), 1.38 (d, *J* = 7.6 Hz, 3H).

^{13}C NMR (101 MHz, $CDCl_3$) δ 202.8, 139.6, 137.4, 136.2, 49.6, 18.7, 10.7.

2-Fluoro-2,4,7-trimethyl-1H-indene-1,3(2*H*)-dione (S2).[1]

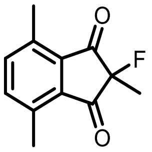

S2

A modified literature procedure[1] was used for the synthesis of **S2**. A solution of **S1** (1.00 g, 5.31 mmol, 1.0 equiv.) and Selectfluor® (2.45 g, 6.91 mmol, 1.3 equiv.) in MeCN (45 mL) was heated at 60 °C for 21 h. The solvent was evaporated under reduced pressure and the residue was partitioned between Et_2O (45 mL) and water (45 mL). The organic layer was separated, washed with water (3 × 30 mL), brine (30 mL) and dried over anhydrous $MgSO_4$. The volatiles were evaporated under reduced pressure. Purification of the crude product by column chromatography (SiO_2, pentane/Et_2O 100:0 to 95:5) yielded product **S2** as white crystals (1.04 g, 5.04 mmol, 95%). Spectroscopic data according to literature[1].

^{1}H NMR (400 MHz, $CDCl_3$) δ 7.54 (s, 2H), 2.72 (s, 6H), 1.63 (d, J = 23.1 Hz, 3H).

^{13}C NMR (101 MHz, $CDCl_3$) δ 196.4 (d, J = 17.2 Hz), 138.7, 137.6 (d, J = 1.3 Hz), 137.5 (d, J = 2.6 Hz), 89.3 (d, J = 194.6 Hz), 18.7, 18.3 (d, J = 26.9 Hz).

^{19}F NMR (376 MHz, $CDCl_3$) δ -166.4 (q, *J* = 23.0 Hz).

3,3'-((1,3)-2-Fluoro-2,4,7-trimethyl-1*H*-indene-1,3(2*H*)-diylidene)bis(1-methylindolin-2-one) (1)

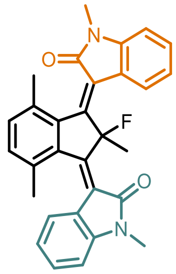

1

An oven-dried crimp top reaction vial under N_2 atmosphere was charged with **S2** (102 mg, 0.495 mmol, 1.0 equiv.) and dry THF (1.5 mL, 0.33 M), and cooled to 0 °C in an ice bath. $TiCl_4$ (0.16 mL, 1.48 mmol, 3.0 equiv.) was added dropwise and the resulting yellow suspension was stirred for 5 min at this temperature. A solution of N-methyl oxindole **S3** (218 mg, 1.48 mmol, 3.0 equiv.) in dry THF (1.0 mL, 1.48 M) was added to the reaction mixture, which was then stirred for 30 min at 0 °C. Subsequently, DBU (0.22 mL, 1.48 mmol, 3.0 equiv.) was added dropwise, and the resulting dark reaction mixture was stirred and heated at 40 °C for 18 h. The reaction was quenched with 1 M aqueous HCl solution and the mixture was extracted with EtOAc (3 x 10 mL). The combined organic layers were washed with brine (15 mL) and dried over $MgSO_4$. The volatiles were removed in vacuo to give a brown oil. The crude product was purified by flash column chromatography (SiO_2, pentane:EtOAc 100:0 to 50:50). The product was obtained as pure (Z_SZ_S)-**1** and (E_SZ_S)-**1** isomers (Z_SZ_S:E_SZ_S 49:51, 68 mg, 0.146 mmol, 30%). TLC: R_f (pentane:EtOAc 50:50) = 0.67 ((E_SZ_S)-**1**), 0.31 ((Z_SZ_S)-**1**).

A solution of (E_SZ_S)-**1** (17 mg, 37 µmol) in degassed CH_2Cl_2 (4.0 mL) was irradiated with 455 nm light at 20 °C for 3 h (analysis with UV/Vis absorption spectroscopy indicated that the PSS was reached). The reaction mixture was concentrated under reduced pressure. The crude product was subjected to flash column chromatography (SiO_2, pentane:EtOAc 100:0 to 50:50), affording the pure stable isomers of product **1** as orange solids (Z_SZ_S:E_SZ_S:E_SE_S 9:52:39, 12 mg, 35 µmol, 68%). TLC: R_f (pentane:EtOAc 50:50) = 0.67 ((E_SZ_S)-**1**), 0.49 ((E_SE_S)-**1**), 0.31 ((Z_SZ_S)-**1**).

(Z_SZ_S)-1

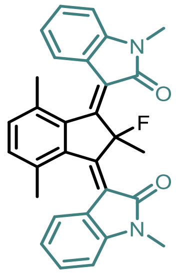

(Z_SZ_S)-**1**

^{1}H NMR (400 MHz, $CDCl_3$) δ 7.39 (s, 2H), 7.23 (td, *J* = 7.7, 1.2 Hz, 2H), 7.01 (dd, *J* = 7.7, 1.2 Hz, 2H), 6.87 (td, *J* = 7.6, 1.1 Hz, 2H), 6.77 (d, *J* = 7.7 Hz, 2H), 3.25 (s, 6H), 2.31 (d, *J* = 17.9 Hz, 3H), 2.26 (s, 6H).

^{13}C NMR (101 MHz, $CDCl_3$) δ 166.0, 154.8 (d, *J* = 18.9 Hz), 143.1, 141.4 (d, *J* = 4.1 Hz), 135.7 (d, *J* = 1.9 Hz), 134.7, 129.6, 124.11, 123.4 (d, *J* = 1.2 Hz), 122.6, 121.2, 109.8 (d, *J* = 209.4 Hz), 107.9, 26.3, 21.7, 20.4 (d, *J* = 26.0 Hz).

^{19}F NMR (376 MHz, $CDCl_3$) δ -131.2 (q, *J* = 17.9 Hz).

HRMS (ESI^+) calcd for $C_{30}H_{26}FN_2O_2^+$ $[M+H]^+$ 465.1973 found 465.1964.

(E_SZ_S)-**1**

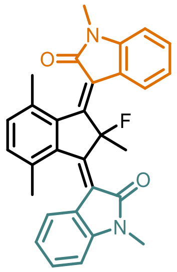

(E_SZ_S)-**1**

^{1}H NMR (400 MHz, $CDCl_3$) δ 7.90 (dd, J = 7.6, 5.4 Hz, 1H), 7.39 (d, J = 8.0 Hz, 1H), 7.32 (d, J = 8.0 Hz, 1H), 7.26 (t, J = 7.6 Hz, 1H), 7.22 (dd, J = 7.7, 1.2 Hz, 1H), 7.05 (dd, J = 7.6, 1.0 Hz, 1H), 7.03 (td, J = 7.7, 1.0 Hz, 1H), 6.89 (td, J = 7.6, 1.0 Hz, 1H), 6.79 (d, J = 5.7 Hz, 1H), 6.77 (d, J = 5.5 Hz, 1H), 3.27 (s, 3H), 3.24 (s, 3H), 2.39 (s, 3H), 2.22 (s, 3H), 2.18 (d, J = 17.9 Hz, 3H).

^{13}C NMR (101 MHz, $CDCl_3$) δ 166.5, 166.2, 153.7 (d, J = 12.2 Hz), 153.5 (d, J = 12.9 Hz), 143.5, 143.5, 141.0 (d, J = 4.7 Hz), 140.6 (d, J = 4.1 Hz), 138.4 (d, J = 1.9 Hz), 134.6 (d, J = 1.8 Hz), 134.3, 134.1, 129.5, 129.1, 127.2 (d, J = 14.0 Hz), 123.4 (d, J = 0.7 Hz), 123.4 (d, J = 0.7 Hz), 123.2, 122.4, 122.0 (d, J = 2.1 Hz), 121.8 (d, J = 1.2 Hz), 121.6, 108.52 (d, J = 208.4 Hz), 107.9, 107.7, 26.3, 26.0, 21.8, 21.5 (d, J = 25.3 Hz), 21.3.

^{19}F NMR (376 MHz, $CDCl_3$) δ -135.37 (qd, J = 17.9, 5.4 Hz).

HRMS (ESI$^+$) calcd for $C_{30}H_{26}FN_2O_2^+$ [M+H]$^+$ 465.1973 found 465.1966.

(E_SE_S)-**1**

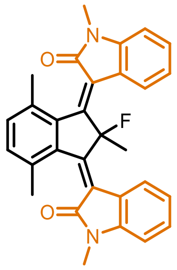

(E_SE_S)-**1**

^{1}H NMR (400 MHz, $CDCl_3$) δ 7.78 (app t, J = 7.7, 5.7 Hz, 2H), 7.32 (s, 2H), 7.26 (t, J = 7.6 Hz, 2H), 7.06 (t, J = 7.6 Hz, 2H), 6.79 (d, J = 7.8 Hz, 2H), 3.23 (s, 6H), 2.35 (s, 6H), 2.05 (d, J = 17.9 Hz, 3H).

^{13}C NMR (101 MHz, $CDCl_3$) δ 165.9, 152.3 (d, J = 19.2 Hz), 143.7, 140.4 (d, J = 4.6 Hz), 137.2 (d, J = 1.8 Hz), 133.6, 128.9, 126.4 (d, J = 13.9 Hz), 121.9 (d, J = 1.1 Hz), 121.8 (d, J = 17.6 Hz), 121.6 (d, J = 2.2 Hz), 107.8, 107.4 (d, J = 206.9 Hz), 26.3, 22.9 (d, J = 24.6 Hz), 21.3.

^{19}F NMR (376 MHz, $CDCl_3$) δ -137.8 (qt, J = 17.9, 5.7 Hz).

3. UV/Vis Absorption Spectra

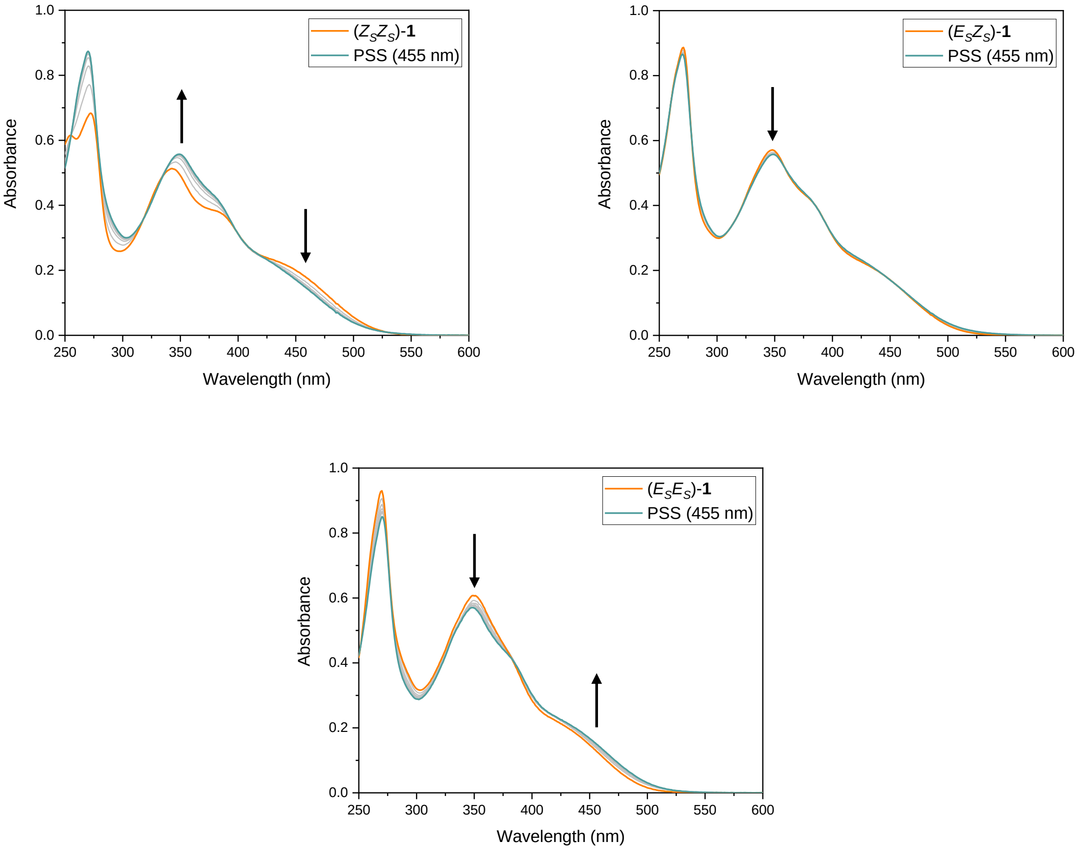

Figure S3. UV/Vis absorption spectra of (Z_SZ_S)-**1** (top left), (E_SZ_S)-**1** (top right) and (E_SE_S)-**1** (bottom) in CH_2Cl_2 (3×10^{-5} M, 20 °C) upon irradiation with 455 nm light. The pure isomer is shown in orange and the obtained PSS in blue.

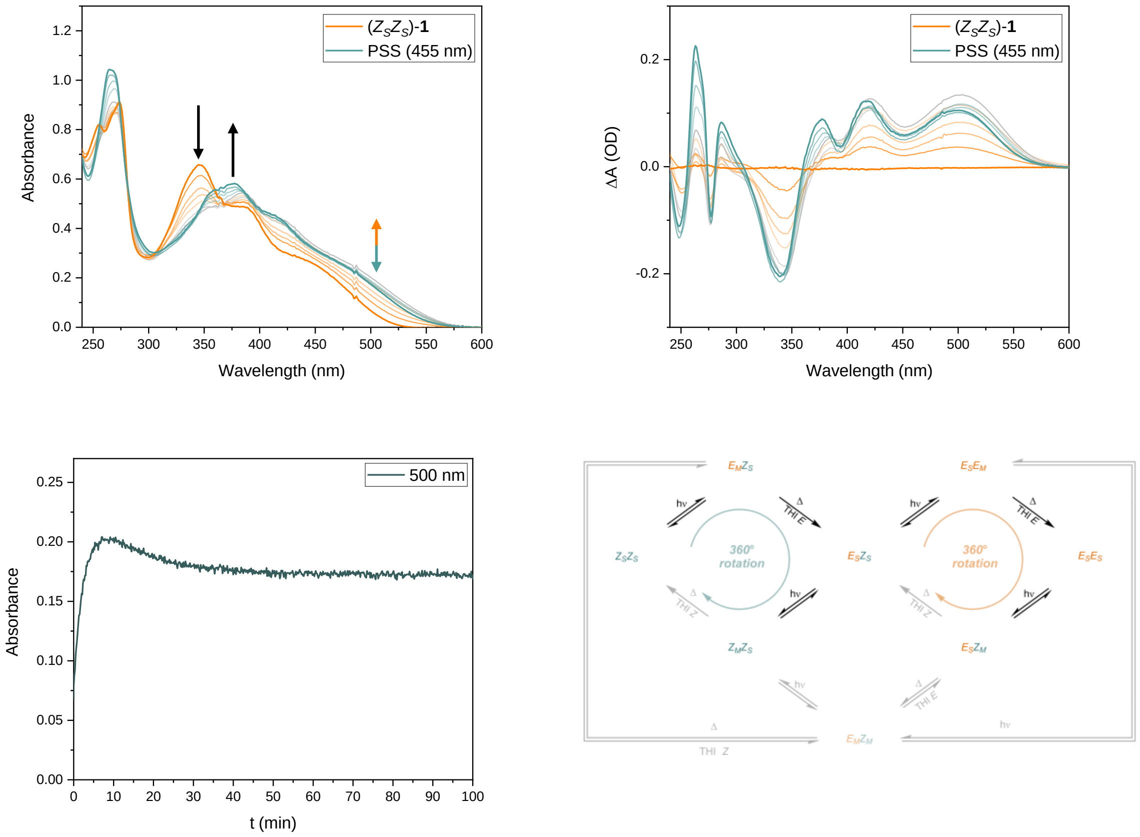

Figure S4. UV/Vis absorption spectra (top left) and difference spectra (top right) of (Z_SZ_S)-**1** in CH_2Cl_2 (4×10^{-5} M, -90 °C) upon irradiation with 455 nm light. The pure isomer is shown in orange and the obtained PSS in blue. The absorbance at 500 nm over time upon irradiation with 455 nm light (bottom left). Motor operation overview under these conditions (bottom right).

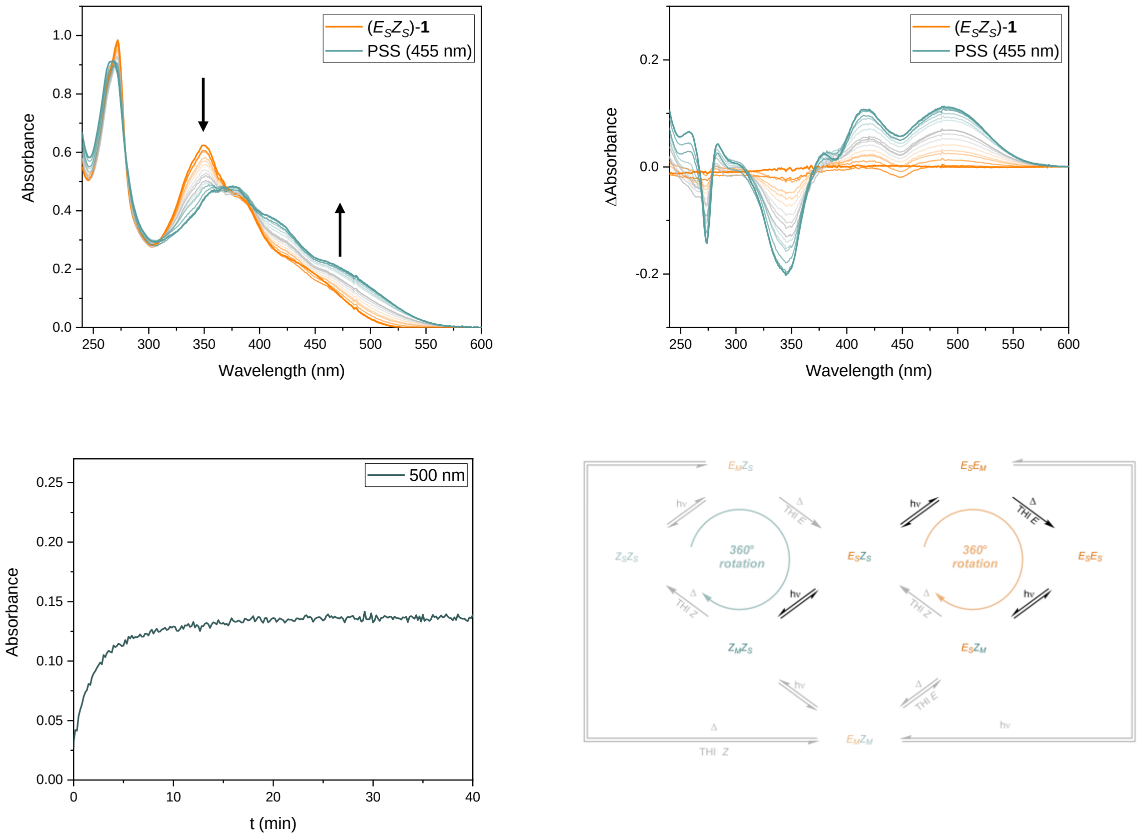

Figure S5. UV/Vis absorption spectra (top left) and difference spectra (top right) of (E_SZ_S)-**1** in CH_2Cl_2 (4×10^{-5} M, -90 °C) upon irradiation with 455 nm light. The pure isomer is shown in orange and the obtained PSS in blue. The absorbance at 500 nm over time upon irradiation with 455 nm light (bottom left). Motor operation overview under these conditions (bottom right).

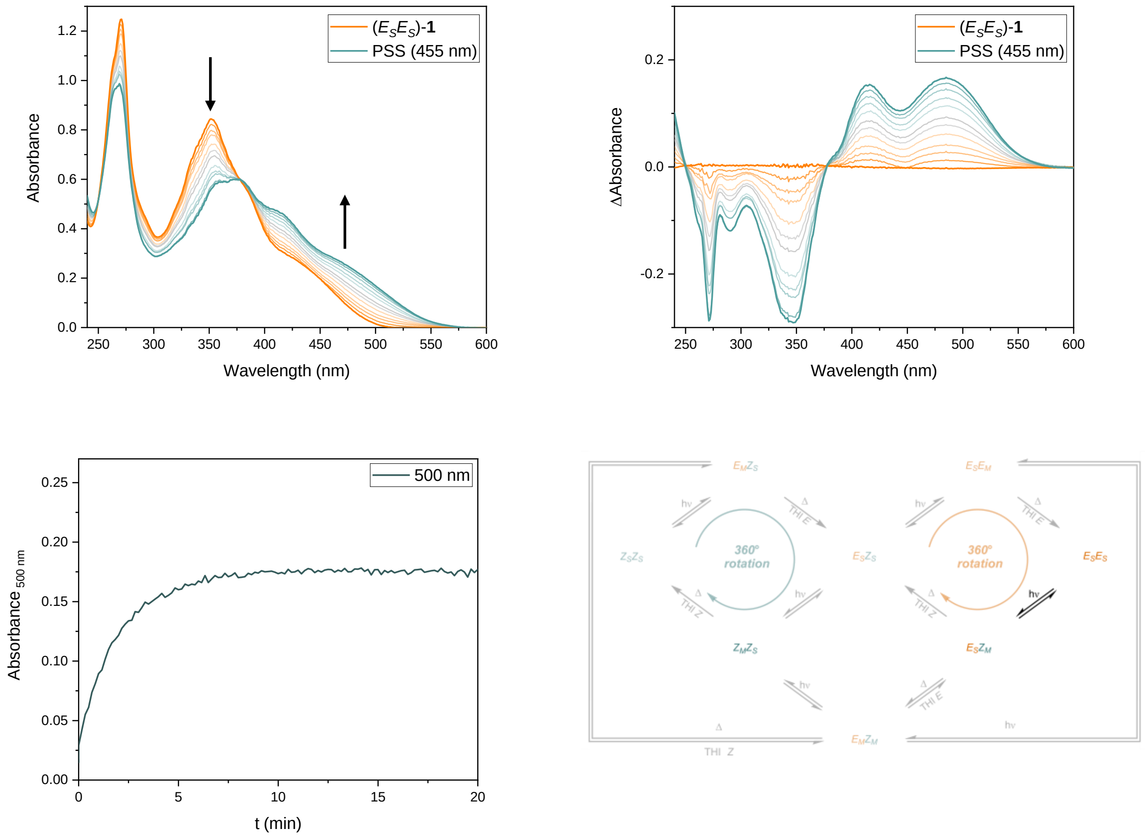

Figure S6. UV/Vis absorption spectra (top left) and difference spectra (top right) of (E_SE_S)-**1** in CH_2Cl_2 (4×10^{-5} M, -90 °C) upon irradiation with 455 nm light. The pure isomer is shown in orange and the obtained PSS in blue. An isosbestic point is maintained at 378 nm. The absorbance at 500 nm over time upon irradiation with 455 nm light (bottom left). Motor operation overview under these conditions (bottom right).

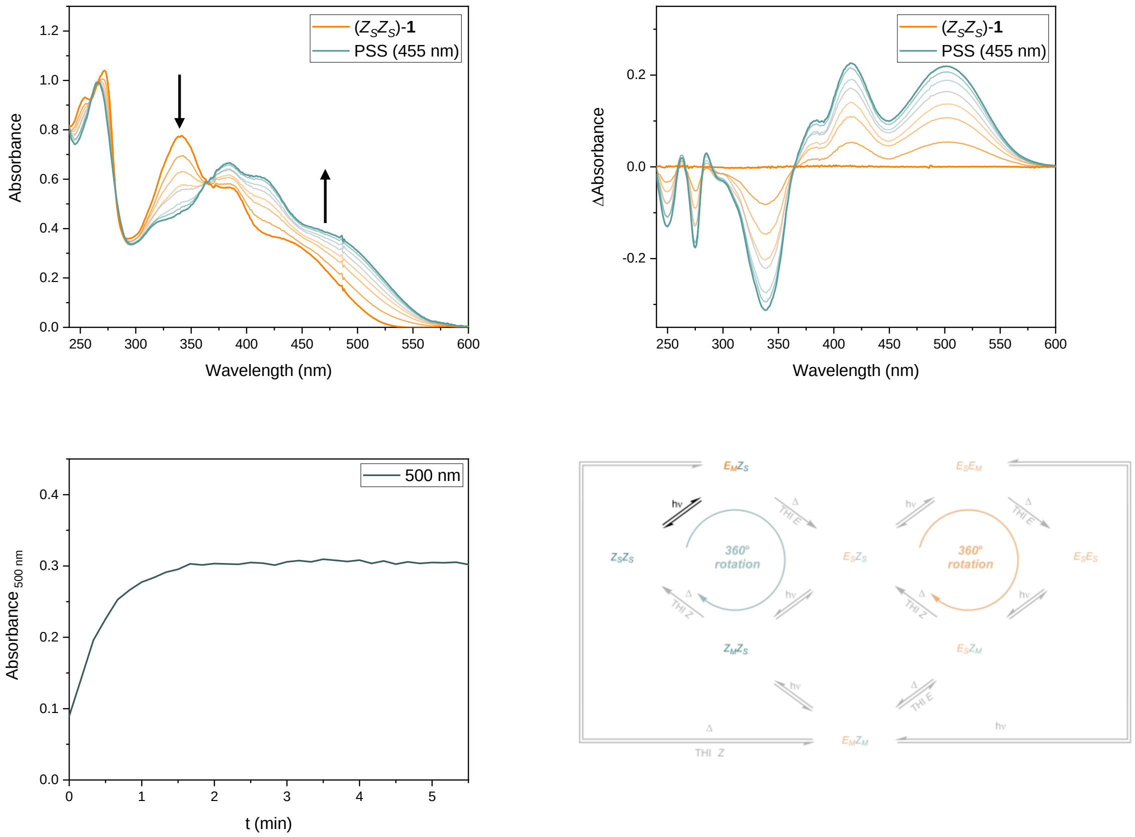

Figure S7. UV/Vis absorption spectra (top left) and difference spectra (top right) of (Z_SZ_S)-**1** in Et_2O (4×10^{-5} M, -110 °C) upon irradiation with 455 nm light. The pure isomer is shown in orange and the obtained PSS in blue. An isosbestic point is maintained at 365 nm. The absorbance at 500 nm over time upon irradiation with 455 nm light (bottom left). Motor operation overview under these conditions (bottom right).

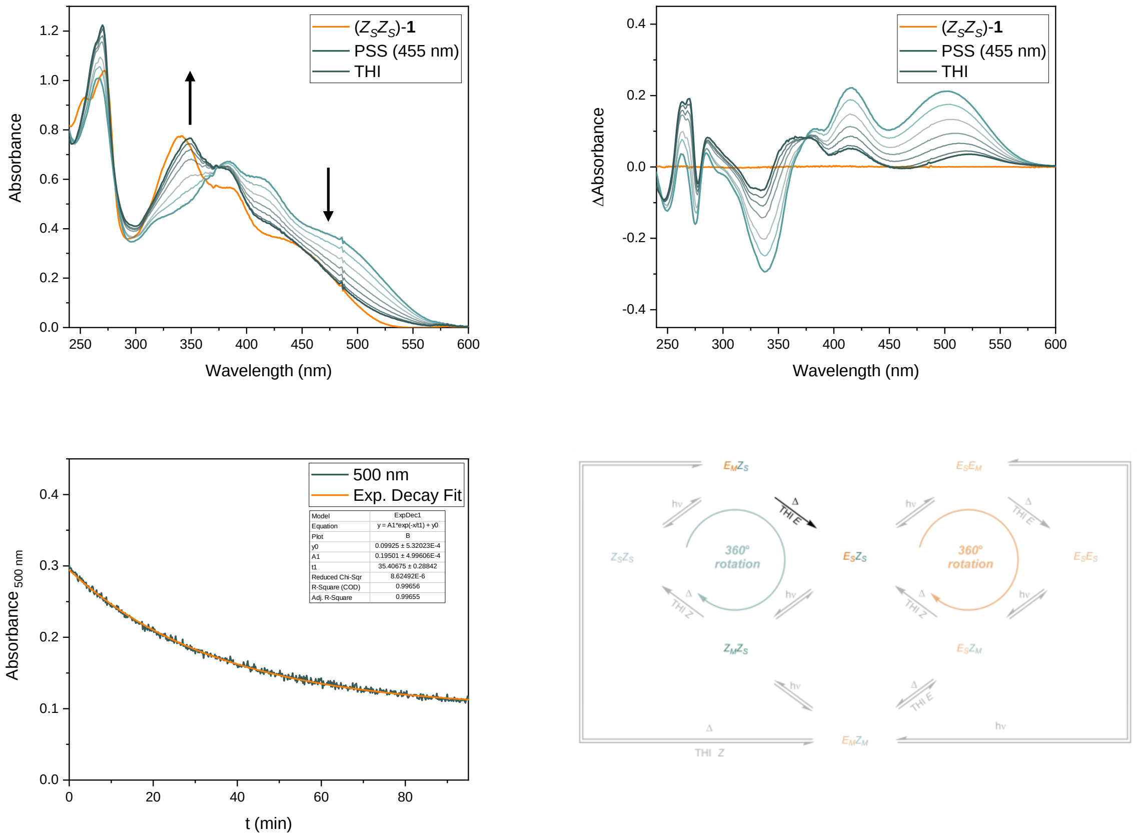

Figure S8. UV/Vis absorption spectra (top left) and difference spectra (top right) of the $PSS_{455\,nm}$ mixture starting from (Z_SZ_S)-**1** in Et_2O (4×10^{-5} M, -110 °C) upon thermal relaxation in the dark. The initial isomer (Z_SZ_S)-**1** is shown in orange, the obtained PSS_{455nm} in blue, and after THI in dark green. An isosbestic point is maintained at 375 nm. The absorbance at 500 nm over time upon irradiation with 455 nm light. The half-life ($t_{1/2}$) was obtained by fitting the data to the mono-exponential decay equation: $A = y_0 + A_1e^{-t/(t1/2)}$ using Origin software (bottom left). Motor operation overview under these conditions (bottom right).

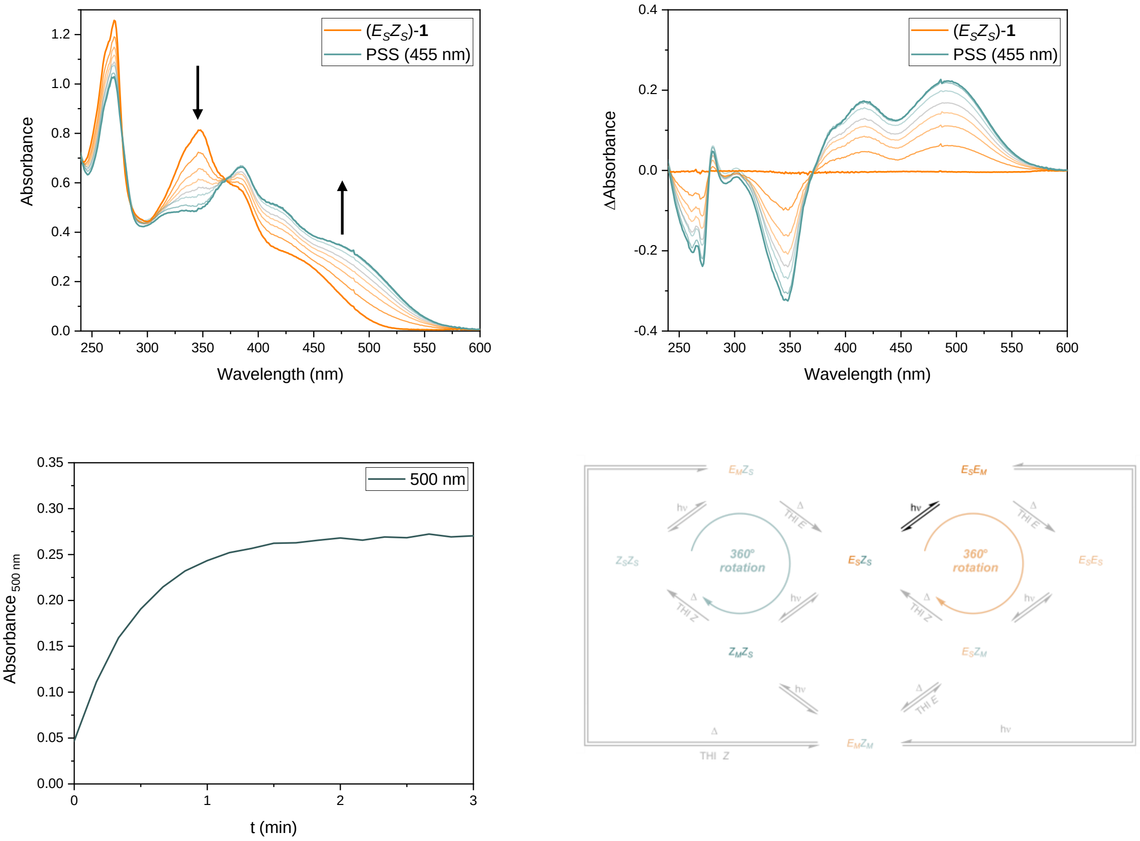

Figure S9. UV/Vis absorption spectra (top left) and difference spectra (top right) of (E_SZ_S)-**1** in Et_2O (4×10^{-5} M, -110 °C) upon irradiation with 455 nm light. The pure isomer is shown in orange and the obtained PSS in blue. An isosbestic point is maintained at 371 nm. The absorbance at 500 nm over time upon irradiation with 455 nm light (bottom left). Motor operation overview under these conditions (bottom right).

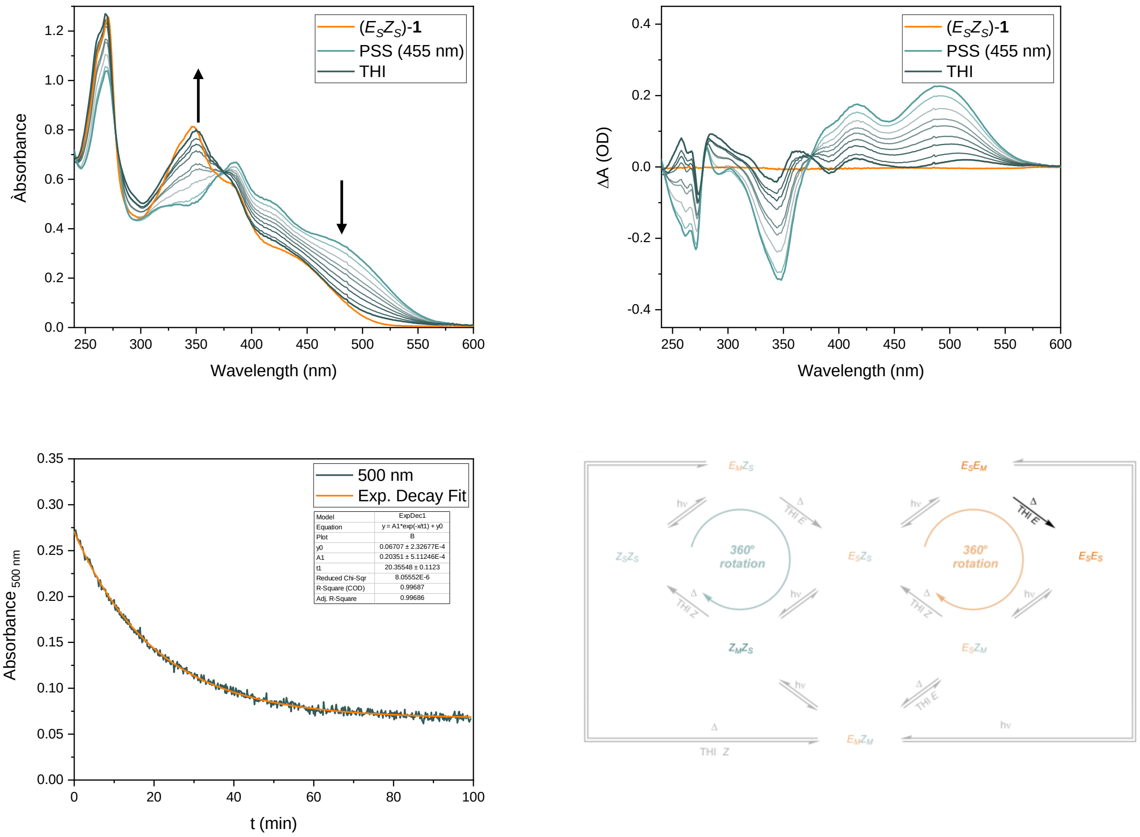

Figure S10. UV/Vis absorption spectra (top left) and difference spectra (top right) of the $PSS_{455\ nm}$ mixture starting from (E_SZ_S)-**1** in Et_2O (4×10^{-5} M, -110 °C) upon thermal relaxation in the dark. The initial isomer (E_SZ_S)-**1** is shown in orange, the obtained PSS_{455nm} in blue, and after THI in dark green. An isosbestic point is maintained at 375 nm. The absorbance at 500 nm over time upon irradiation with 455 nm light. The half-life ($t_{1/2}$) was obtained by fitting the data to the mono-exponential decay equation: $A = y_0 + A_1e^{-t/(t1/2)}$ using Origin software (bottom left). Motor operation overview under these conditions (bottom right).

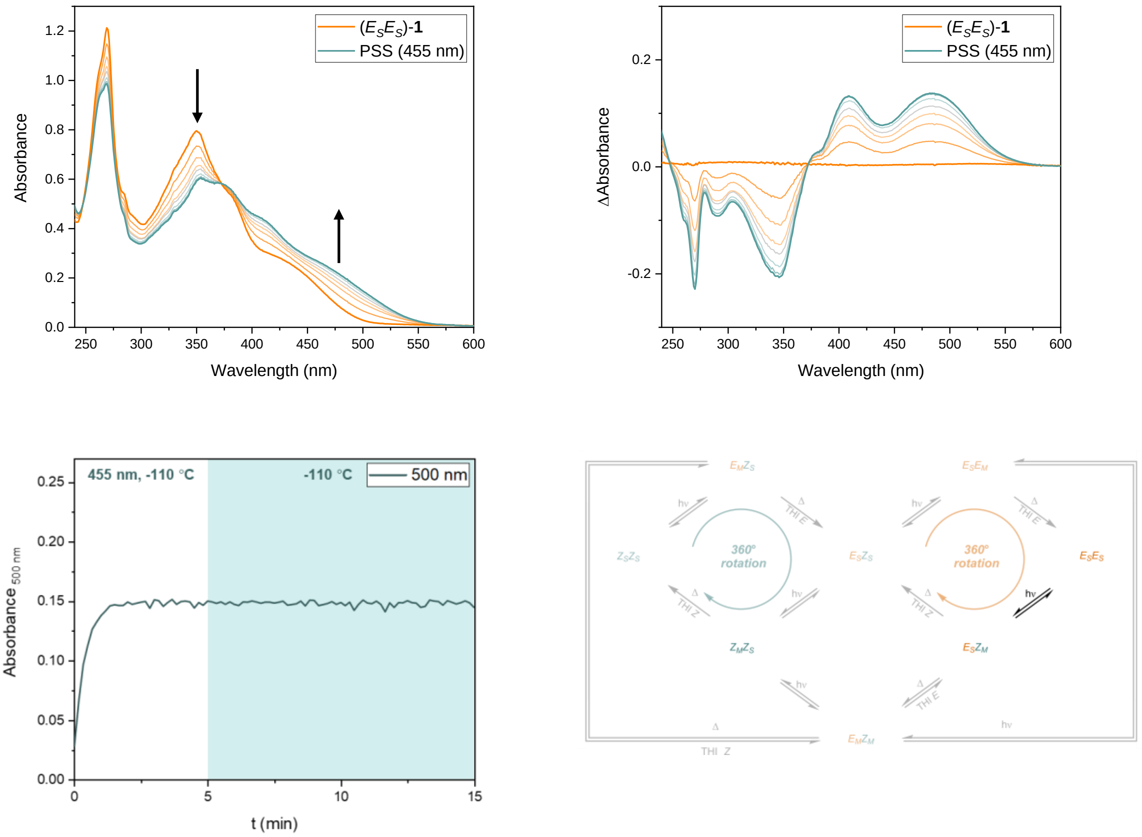

Figure S11. UV/Vis absorption spectra (top left) and difference spectra (top right) of (E_SE_S)-**1** in Et_2O (4×10^{-5} M, -110 °C) upon irradiation with 455 nm light. The pure isomer is shown in orange and the obtained PSS in blue. An isosbestic point is maintained at 372 nm. The absorbance at 500 nm over time upon irradiation with 455 nm light (5 min) and in the dark (10 min) (bottom left). Motor operation overview under these conditions (bottom right).

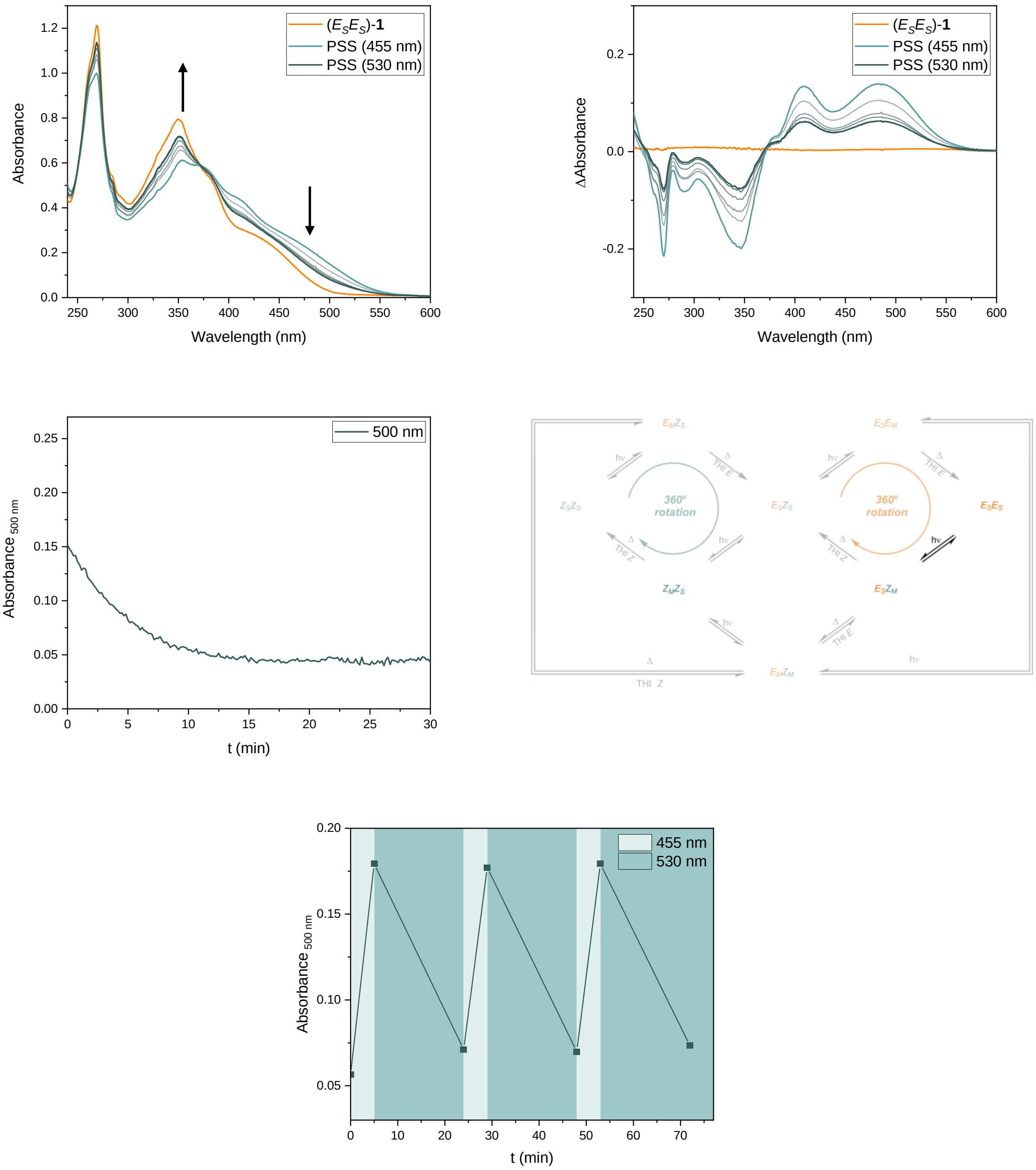

Figure S12. UV/Vis absorption spectra (top left) and difference spectra (top right) of the $PSS_{455\ nm}$ mixture starting from (E_SE_S)-**1** in Et_2O (4×10^{-5} M, -110 °C) upon irradiation with 530 nm light. The initial isomer (E_SE_S)-**1** is shown in orange, the obtained PSS_{455nm} in blue, and the obtained PSS_{530nm} dark green. An isosbestic point is maintained at 372 nm. Absorbance at 500 nm over time upon irradiation with 530 nm light (middle left). Motor operation overview under these conditions (middle right). Fatigue study of (E_SE_S)-**1**, three cycles of 455 nm light (5 min) followed by 530 nm light (19 min) (bottom).

4. NMR Studies and Kinetic Experiments

NMR Irradiation Experiments

Room temperature experiments

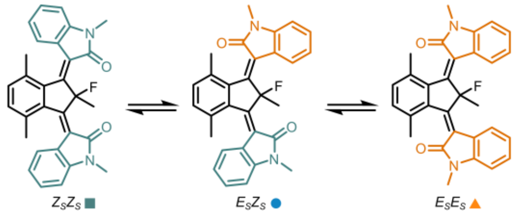

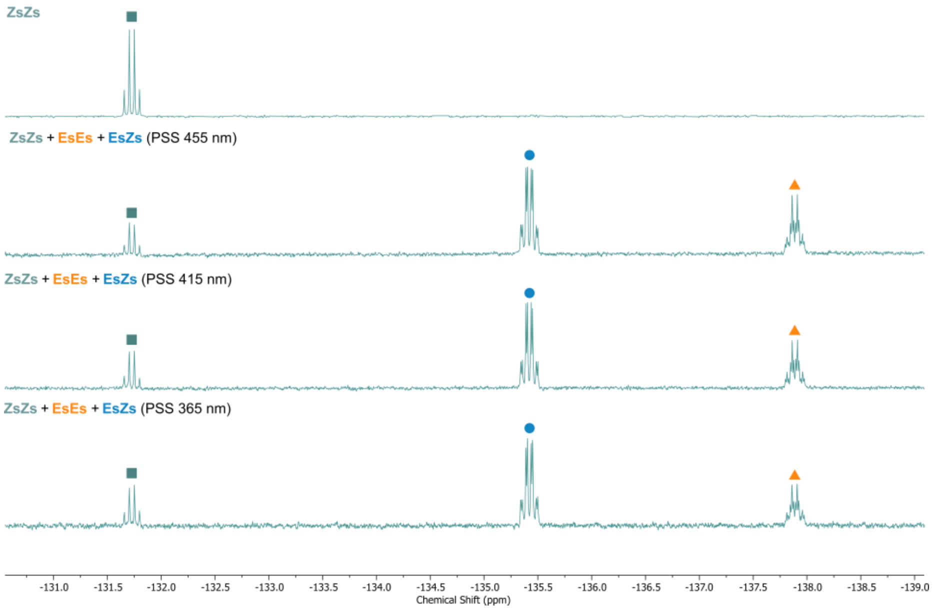

λ (nm)	PSS<br>*ZsZs : EsZs : EsEs*
455	9 : 54 : 37
415	12 : 55 : 33
365	14 : 57 : 30

Figure S13. Determination of the PSS ratios achieved upon irradiation with 455 nm, 415 nm and 365 nm starting from of (Z_SZ_S)-**1** in CD_2Cl_2 (20 °C) using ^{19}F NMR spectroscopy (376 MHz). The same PSS ratios were obtained when starting from (E_SZ_S)-**1** instead of (Z_SZ_S)-**1**.

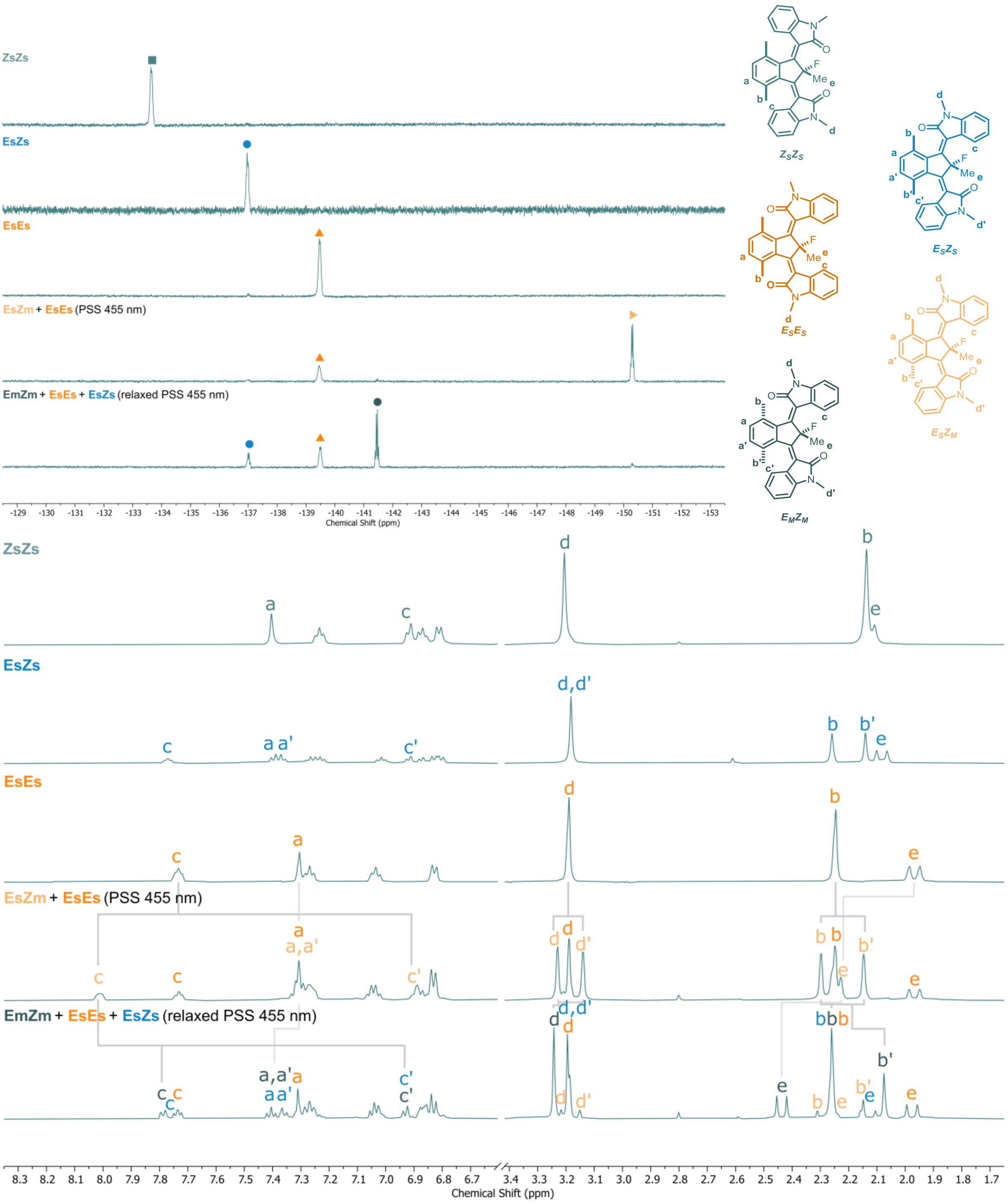

Figure S14. Top: ^{19}F NMR spectra (470 MHz, -85 °C, CD_2Cl_2) of (*Z*s*Z*s)-**1**, (*E*s*Z*s)-**1**, (*E*s*E*s)-**1**, a sample enriched with (*E*s*Z*M)-**1**, and a sample enriched with (*E*M*Z*M)-**1**. Bottom: ^{1}H NMR spectra (500 MHz, -85 °C, CD_2Cl_2) of (*Z*s*Z*s)-**1**, (*E*s*Z*s)-**1**, (*E*s*E*s)-**1**, a sample enriched with (*E*s*Z*M)-**1**, and a sample enriched with (*E*M*Z*M)-**1**. Key signals are assigned with letters.

Low temperature experiments

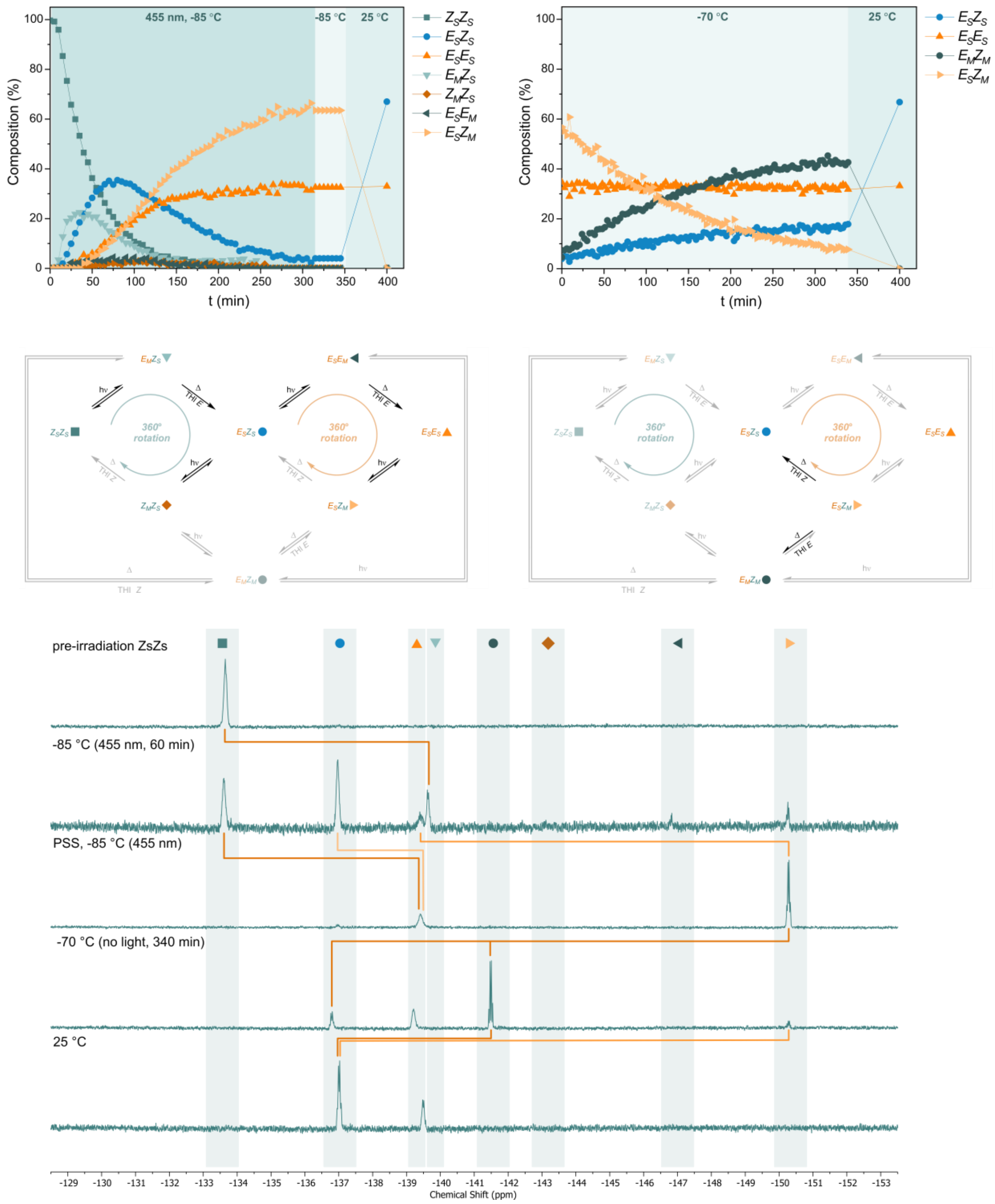

Figure S15. Top left: Sample of (Z_SZ_S)-**1** irradiated to PSS with 455 nm at -85 °C. The sample was subsequently kept at -85 °C for 45 min and warmed up to room temperature. All measurements were performed at -85 °C. Top right: Thermal decay of pre-irradiated (Z_SZ_S)-**1** (PSS with 455 nm at -85 °C) at -70 °C. The sample was subsequently warmed up to room temperature. The measurements were performed at -70 °C. Middle: Motor operation overview under these conditions. Bottom: The corresponding ^{19}F NMR spectra (470 MHz, 5 mM in CD_2Cl_2) of (Z_SZ_S)-**1** recorded at -85 °C except the spectrum after partial and full relaxation (two spectra from the bottom), which was recorded at -70 °C and -25 °C, respectively.

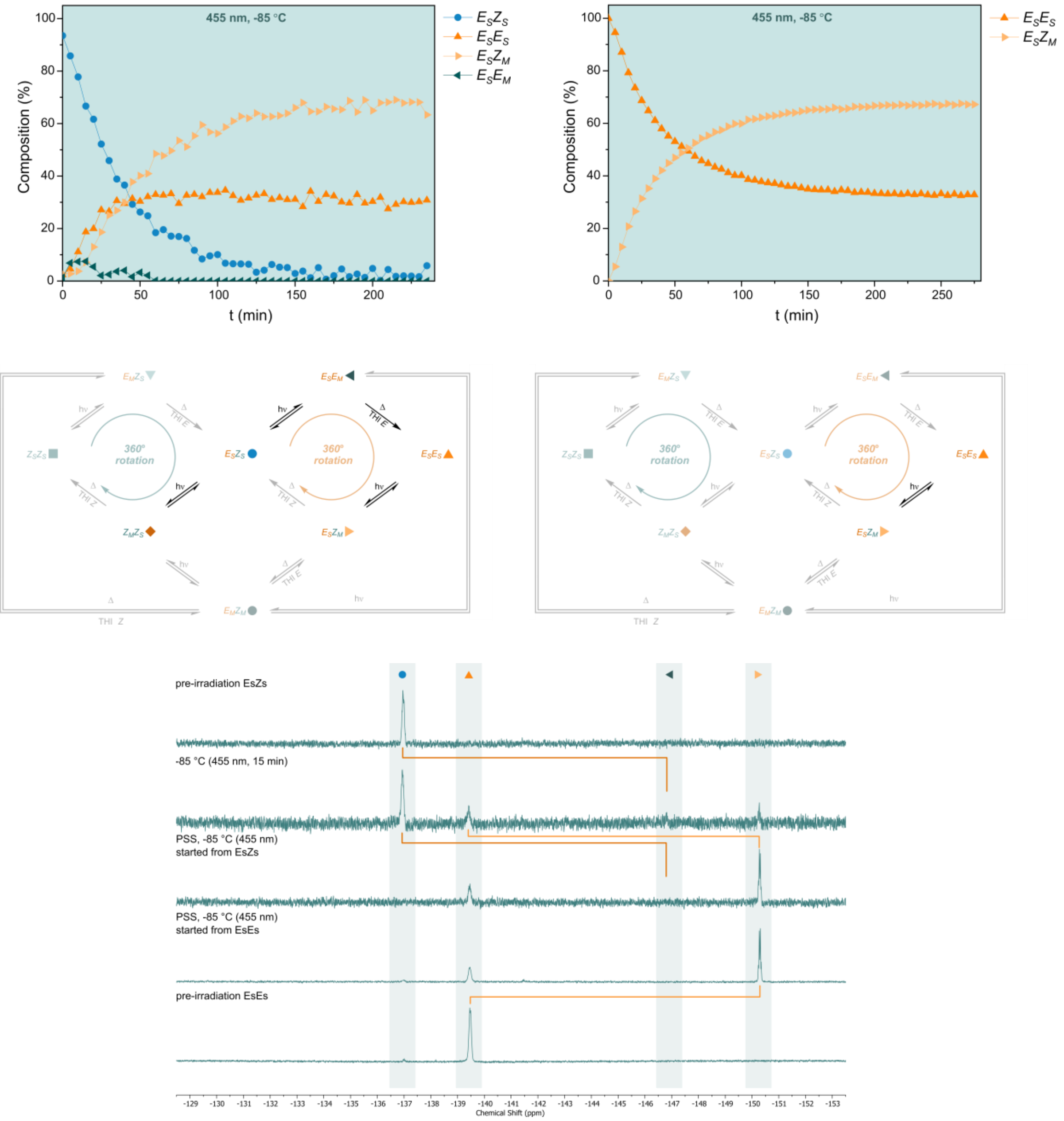

Figure S16. Top left: Sample of (E_SZ_S)-**1** irradiated to PSS with 455 nm at -85 °C. Top right: Sample of (E_SE_S)-**1** irradiated to PSS with 455 nm at -85 °C. Middle: Motor operation overview under these conditions. Bottom: The corresponding ^{19}F NMR spectra (470 MHz, -85 °C) of (E_SZ_S)-**1** (5 mM in CD_2Cl_2) and (E_SE_S)-**1** (11 mM in CD_2Cl_2).

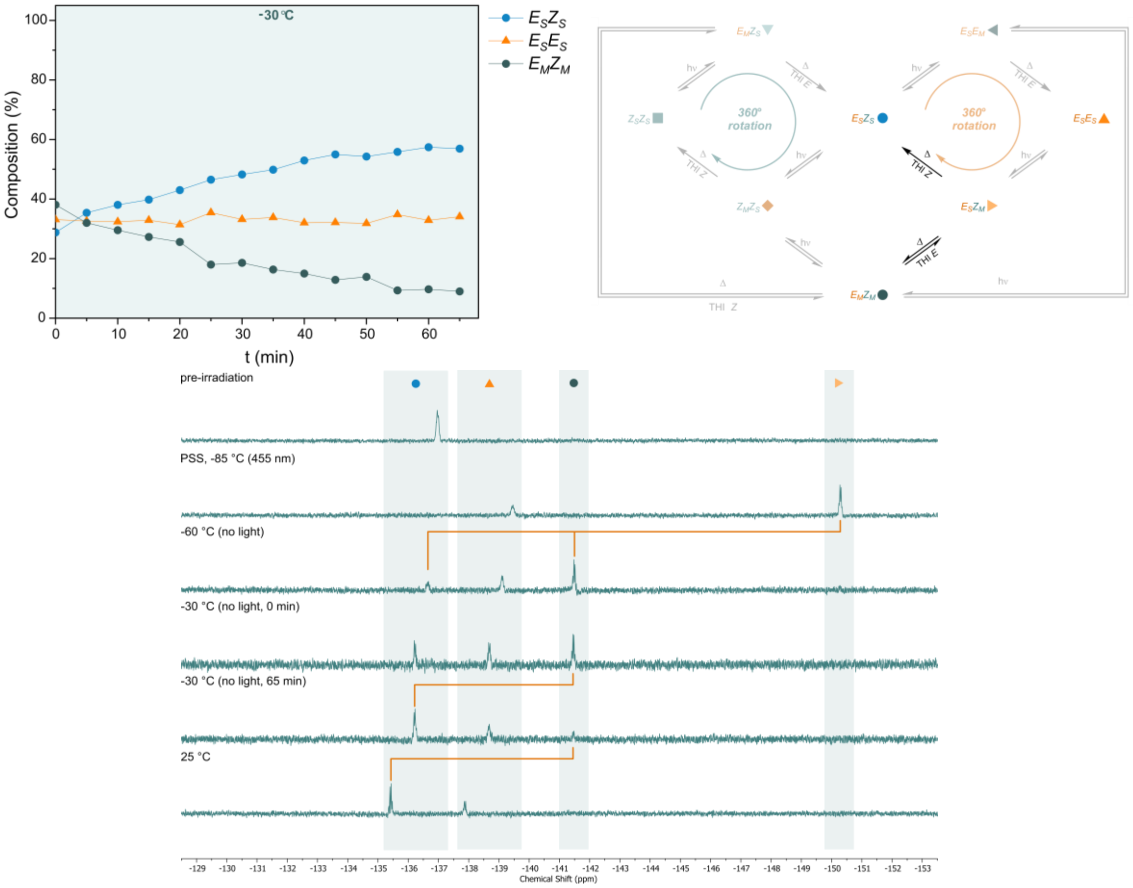

Figure S17. Left: Thermal decay of pre-irradiated (Z_SE_S)-**1** (PSS with 455 nm at -85 °C) at -30 °C (65 min). Right: Motor operation overview under these conditions. Bottom: The corresponding ^{19}F NMR spectra (470 MHz, 5 mM in CD_2Cl_2) of **1** recorded at the labelled temperatures.

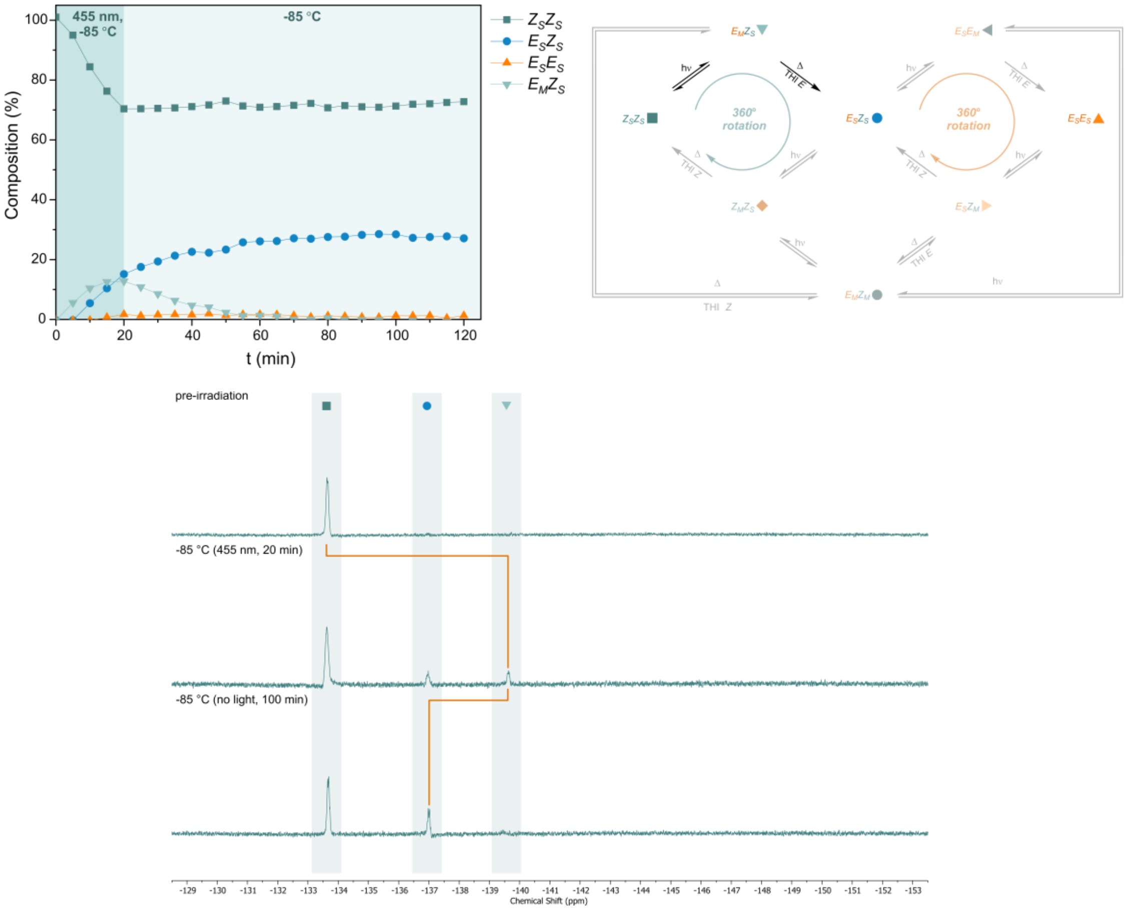

Figure S18. Left: Thermal decay of pre-irradiated (Z_SZ_S)-**1** (455 nm for 20 min at -85 °C) at -85 °C. All measurements were performed at -85 °C. Right: Motor operation overview under these conditions. Bottom: The corresponding ^{19}F NMR spectra (470 MHz, -85 °C, 5 mM in CD_2Cl_2) of **1**.

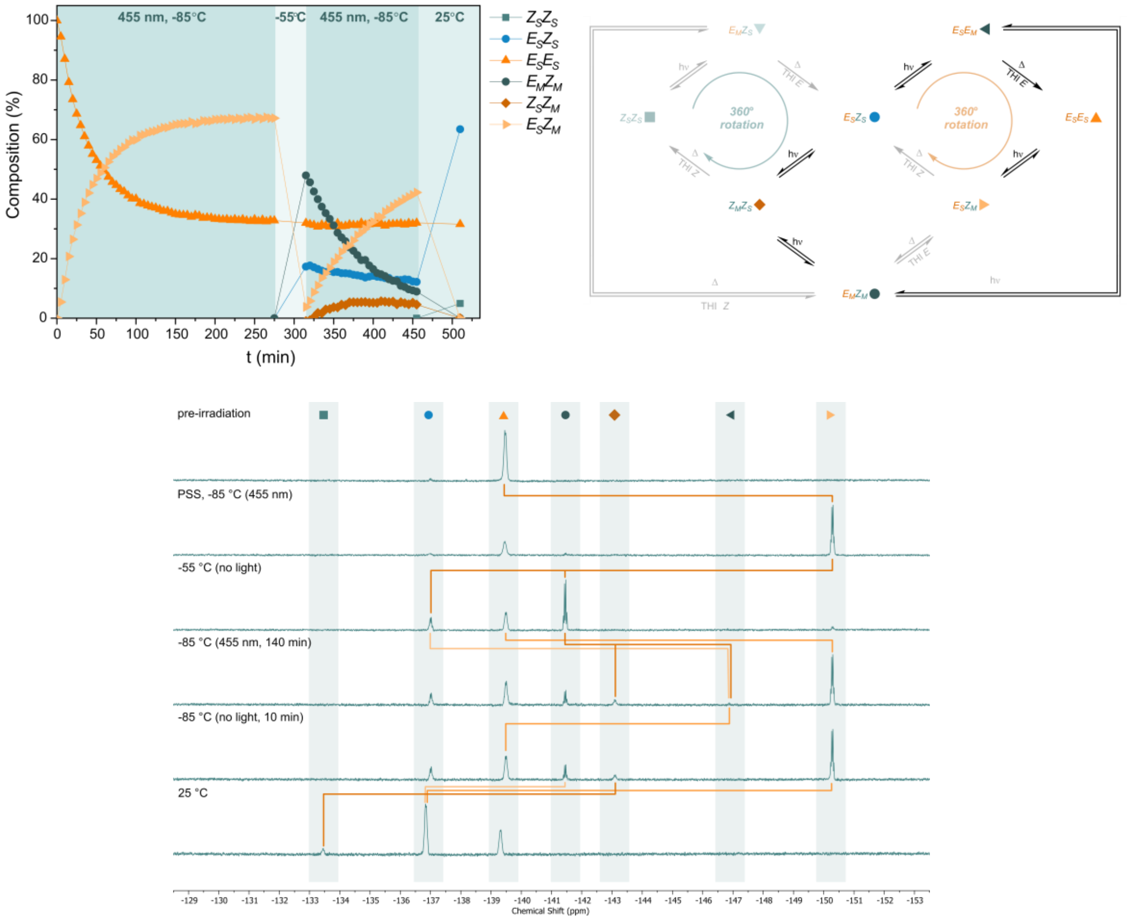

Figure S19. Left top: A sample enriched with (E_MZ_M)-**1** (prepared by thermal relaxation of pre-irradiated (E_SE_S)-**1** (PSS with 455 nm at -85 °C) at -55 °C) irradiated with 455 nm for 140 min at -85 °C, in the dark for 10 min at -85 °C and subsequently warmed up to room temperature. The measurements were performed at the labelled temperatures. Right top: Motor operation overview under these conditions (t = 320 – 460 min) . Bottom: The corresponding ^{19}F NMR spectra (470 MHz, -85 °C, 11 mM in CD_2Cl_2) of **1**.

To further support the presence of (E_MZ_M)-**1**, we provide a zoom in of the stacked NMR spectra for the second irradiation process shown in Figure S19 (irradiation of the sample enriched with (E_MZ_M)-**1**). This is a zoom in of the data set shown in Figure S26.

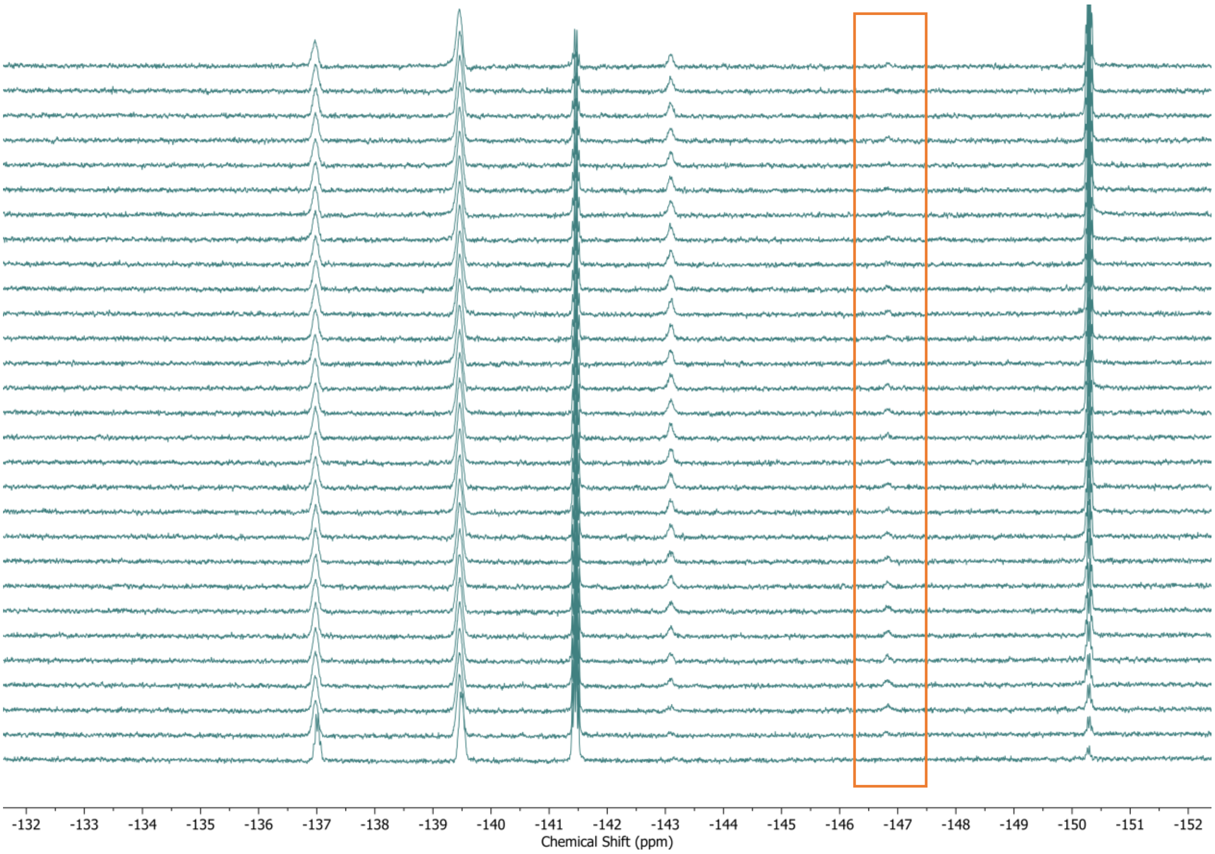

Figure S20. Zoom in of ^{19}F NMR data of a sample enriched with (E_MZ_M)-**1** upon irradiation to PSS with 455 nm light at -85 °C in CD_2Cl_2 (spectra taken every 5 min.). The initial spectrum (bottom spectrum) contains a mixture of (E_MZ_M)-**1**, (E_SZ_S)-**1** and (E_SE_S)-**1**. The orange box highlights the changes of the signal at ± -146.8 ppm (the signal of (E_SE_M)-**1**).

Investigation of selective rotor activation

E/Z photoisomerisation of (E_SZ_S)-**1** can in principle occur at either the *E* rotor or the *Z* rotor, resulting in (E_SE_M)-**1** or (Z_MZ_S)-**1**, respectively (Figure S21). Low temperature UV/Vis absorption studies of (E_SZ_S)-**1** suggested a strong preference for photoisomerisation of the *Z* rotor (Figure S9 and Figure S10). To further investigate the rotor activation selectivity, the initial kinetics of (E_SZ_S)-**1** upon irradiation with different wavelengths were analysed. The obtained distribution of the PSS ratios obtained by irradiating (E_SZ_S)-**1** with light at room temperature are a result of all steps in the motor operation (Figure S13). It can be assumed that all thermal steps are accessible under these conditions, but the analysis of the two photoisomerisation steps of interest is still obstructed by the other concurrent photochemical steps. The initial irradiation kinetics on the other hand can provide insight in the two (E_SZ_S)-**1** photoisomerisation paths and thus in the potentially preferential rotor activation of (E_SZ_S)-**1**. Irradiation of (E_SZ_S)-**1** at 20 °C yields the PSS mixtures within several minutes (Figure S22). As a consequence, analysis by NMR spectroscopy proved to be unable to provide additional insights regarding selective rotor activation.

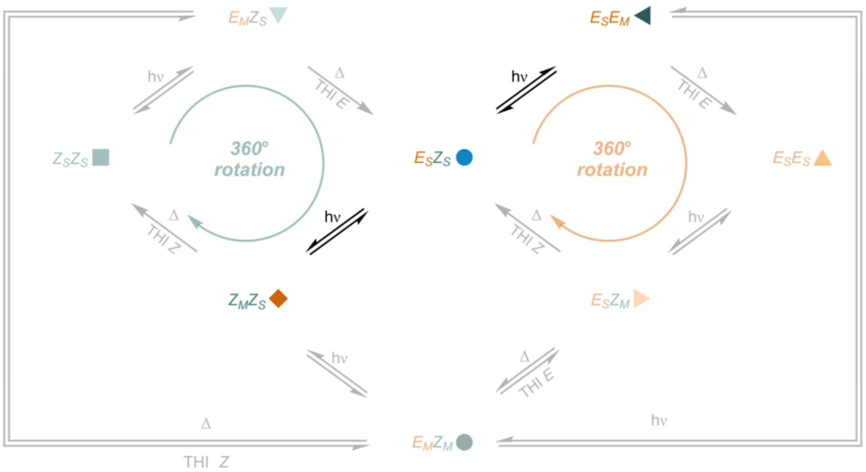

Figure S21. Motor operation overview upon *E/Z* photoisomerisation of (E_SZ_S)-**1** in the absence of thermal relaxation of the metastable states (E_SE_M)-**1** and (Z_MZ_S)-**1**.

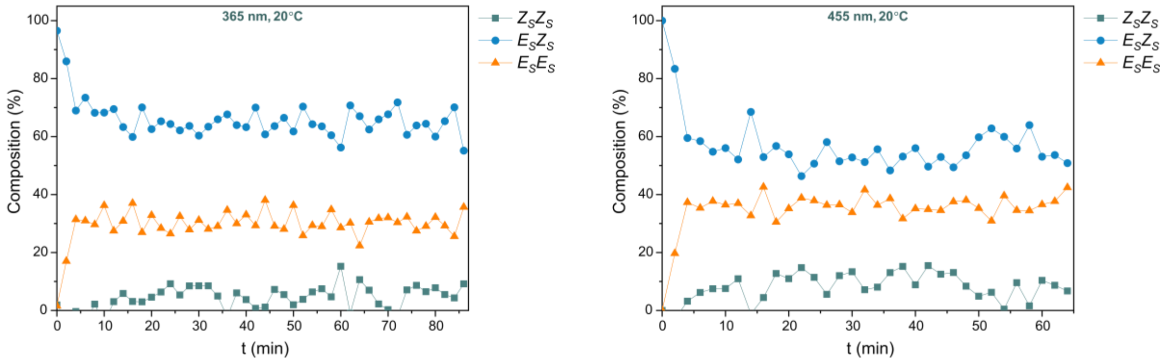

Figure S22. Composition over time of a sample of (E_SZ_S)-**1** in CD_2Cl_2 irradiated to PSS with 365 nm (left) or 455 nm (right) at 20 °C followed by ^{19}F NMR spectroscopy (470 MHz).

To isolate the photoisomerisation processes from the thermal steps, the irradiation experiments of (E_SZ_S)-**1** were repeated at – 110 °C. This temperature minimises thermal relaxation during the irradiation time required to reach the PSS. UV/Vis absorption spectroscopy was used to confirm reaching the PSS. The PSS sample was warmed up to room temperature in the dark, concentrated and analysed by NMR spectroscopy. Assuming no thermal relaxation occurred during irradiation of the sample, the determined PSS ratio of (E_SZ_S)-**1**, (E_SE_S)-**1** and (Z_SZ_S)-**1** after relaxation should be identical to PSS ratio of (E_SZ_S)-**1**, (E_SE_M)-**1** and (Z_MZ_S)-**1** at low temperature (before relaxation). These experiments showed that the photo-equilibria of (E_SZ_S)-1 lie strongly towards (E_SE_M)-1 (

Figure S23). The preference for activation of the *Z* rotor of (E_SZ_S)-**1** is present for all tested irradiation wavelengths. The reason for the observed selectivity for rotation of the *Z* rotor remains unknown. The net bias towards (E_SE_M)-**1** could originate from 1) relatively efficient back switching from (Z_MZ_S)-**1** to (E_SZ_S)-**1** compared to the back switching from (E_SE_M)-**1** to (E_SZ_S)-**1** and/or 2) efficient forward switching from (E_SZ_S)-**1** to (E_SE_M)-**1** relative to (E_SZ_S)-**1** to (Z_MZ_S)-**1**.

Changing the irradiation wavelength can be used to modulate the PSS mixture, but in no case considerable formation of (Z_SZ_S)-**1** (and thus (Z_MZ_S)-**1**) was observed. The necessity to use low sample concentration required for short irradiation times in combination and the unfavoured conversion from (E_SZ_S)-**1** to (Z_MZ_S)-**1** could provide an explanation for the absence of (Z_SZ_S)-**1** in the NMR spectra.

The use of a more blue-shifted wavelength was experimentally not feasible. For irradiation with 340 nm light, photoisomerisation of E_SZ_S could not be isolated as extended irradiation times were required due to the lower power output of the 340 nm LED. Hence for this experiment, considerable thermal relaxation of E_SE_M to E_SE_S occurred before the PSS was reached.

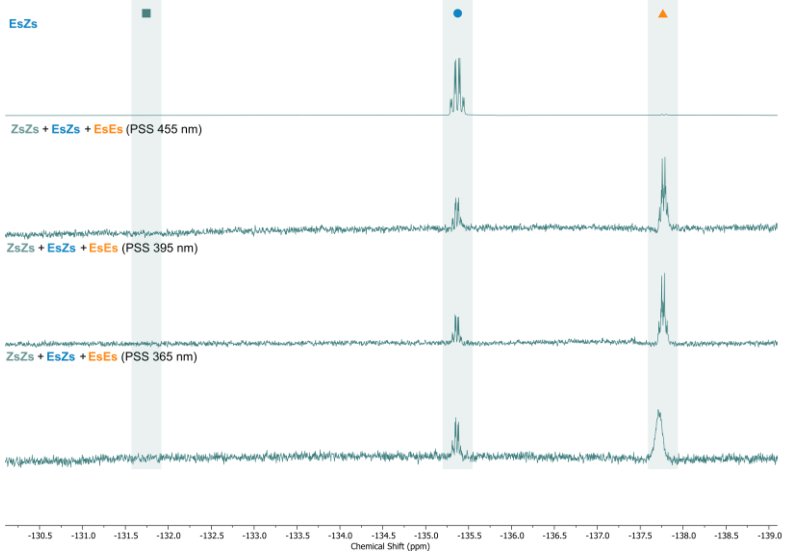

λ (nm)	PSS[a] ZsZs : EsZs : EsEs	PSS[b] ZsZs : EsZs : EsEs
455	0 : 30 : 70	0 : 27 : 73
395	0 : 25 : 75	0 : 22 : 78
365	0 : 19 : 82	0 : 16 : 84

[a] Ratio determined by ^{19}F NMR spectroscopy
[b] Ratio determined by ^{1}H NMR spectroscopy

Figure S23. Determination of the PSS ratios achieved upon irradiation of (E_SZ_S)-**1** with 455 nm, 395 nm and 365 nm in Et_2O (-110 °C) and subsequent warming up in the dark to 20 °C. The composition of the relaxed sample was determined using ^{1}H and ^{19}F NMR spectroscopies ($CDCl_3$, 20 °C). ^{19}F NMR spectra of (E_SZ_S)-**1** and relaxed PSS mixtures of different irradiation wavelengths (top). Summary of the obtained PSS ratios with different irradiation wavelengths (bottom).

Kinetic Analysis

The experimental data was fitted using Origin (for one to one processes) and COPASI[2] (for multi-step processes). The used time unit was minutes. Fitting of the data using Origin was done with the default mono-exponential decay equation: $A = y_0 + A_1e^{-t/(t1/2)}$, which provided the half-life ($t_{1/2}$) of interest. Fitting of the data using COPASI was performed using the default Levenberg-Marquardt algorithm with a tolerance of $1\cdot10^{-6}$ and an run iteration limit of 3000. The initial guesses of the rate constants for the kinetic parameter estimation were 1) that all species are in equilibrium with each other and 2) initial values of the kinetic constants were random values. Visual inspection of the value and associated error of each kinetic constant provided an indication of the relevance of each reaction. Kinetic constants with absolute values lower than 10^{-6} min^{-1} were approximated to 0. The respective reaction was deleted in the next iteration. This iterative process of fitting, inspection and model adaptation was repeated until the parameter estimation results no longer showed values indicative for further improvements to the model.

The kinetics constants were determined using various NMR experiments performed in CD_2Cl_2. The results are summarised in Table S1. Two kinetic constants, for the processes THI_E (E_MZ_S,E_SZ_S) and THI_E (E_ME_S,E_SE_S), were determined using three different types of experiments. For the experiments at -110 °C, Et_2O was used instead of CD_2Cl_2 or CH_2Cl_2 as dichloromethane would freeze at this temperature. The difference of 1.5-2.0 kcal mol^{-1} between the barriers determined in Et_2O and CD_2Cl_2 is hypothesised to be a viscosity effect. Motors operation in solvents with a lower viscosity typically have a lower activation barrier for their THI steps.[3] In our experience Et_2O at -110 °C appeared less viscous than CD_2Cl_2 and CH_2Cl_2 at -85 °C. For details on the calculated barriers, the reader is referred to the Computational Details section on page 38. Notably, the CO-flip irreversibly forms (E_MZ_M)-**1** from (E_SZ_M)-**1** at -70 °C while full relaxation of (E_MZ_M)-**1** to (E_SZ_S)-**1** can only be achieved at temperatures above -45 °C. The latter two-step process proceeded without observing (E_SZ_M)-**1** which together with comparison of calculated and experimental barriers provided insufficient evidence for assigning the rate-determining step (see also p. 33). The absence of (E_SZ_M)-**1** could suggest that the reverse CO-flip is rate-limiting but could equally arise from a THI_Z rate determining step in combination with a (E_MZ_M)-**1**/(E_SZ_M)-**1** thermal equilibrium essentially shifted completely towards (E_MZ_M)-**1**.

Table S1. *Overview of experimentally determined kinetic constants of the various THI steps under different conditions. Experiments marked with * were performed in Et_2O instead of CD_2Cl_2.*

	T (°C)	k (min^{-1})	$t_{1/2}$ (min)	$\Delta^‡G$ (kcal mol^{-1})	$\Delta^‡G_{calc}$ (kcal mol^{-1})	Details
THI_E (E_MZ_S,E_SZ_S)						
Shock *in situ* NMR irradiation of Z_SZ_S	-85	$2.9\cdot10^{-2}$	24.1	13.7	15.5	Figure S27
In situ NMR irradiation of Z_SZ_S	-85	$4.8\cdot10^{-2}$	14.4	13.5	15.5	Figure S24
UV/Vis absorption relaxation trace E_MZ_S*	-110	$2.0\cdot10^{-2}$	35.4	12.0	15.4	Figure S7
THI_E (E_ME_S,E_SE_S)						
In situ NMR irradiation of E_MZ_M	-85	$3.4\cdot10^{-1}$	2.0	12.8	15.1	Figure S26
In situ NMR irradiation of Z_SZ_S	-85	$1.9\cdot10^{-1}$	3.6	13.0	15.1	Figure S24
UV/Vis absorption relaxation trace E_ME_S*	-110	$3.4\cdot10^{-2}$	20.4	11.8	15.0	Figure S10
THI_Z (E_SZ_M,E_SZ_S)						
NMR relaxation of E_SZ_M	-70	$1.5\cdot10^{-3}$	448	16.0	18.2	Figure S25
CO-flip = THI_E (E_SZ_M,E_MZ_M)						
NMR relaxation of E_SZ_M	-70	$4.7\cdot10^{-3}$	148	15.6	16.7	Figure S25
THI_Z (E_MZ_M,E_SZ_S)						
NMR relaxation of E_MZ_M	-30	$1.8\cdot10^{-2}$	37.8	18.0	20.0[a]<br>21.0[b]	Figure S28

[a] reverse CO-flip (THI_E (E_MZ_M, E_SZ_M)) as the rate-determining step, see p. 33 for a detailed discussion.

[b] THI_Z (E_SZ_M, E_SZ_S) as the rate-determining step, see p. 33 for a detailed discussion.

Relaxation kinetics of E_MZ_S and E_SE_M at -85 °C

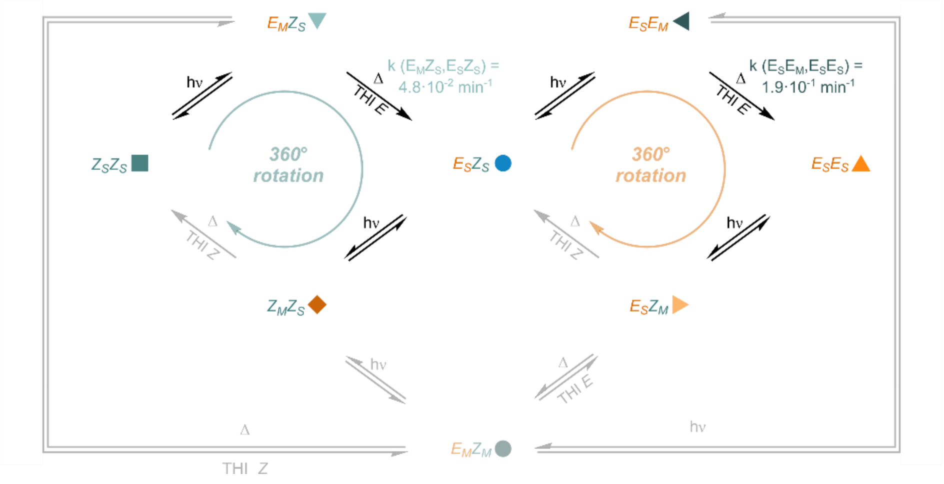

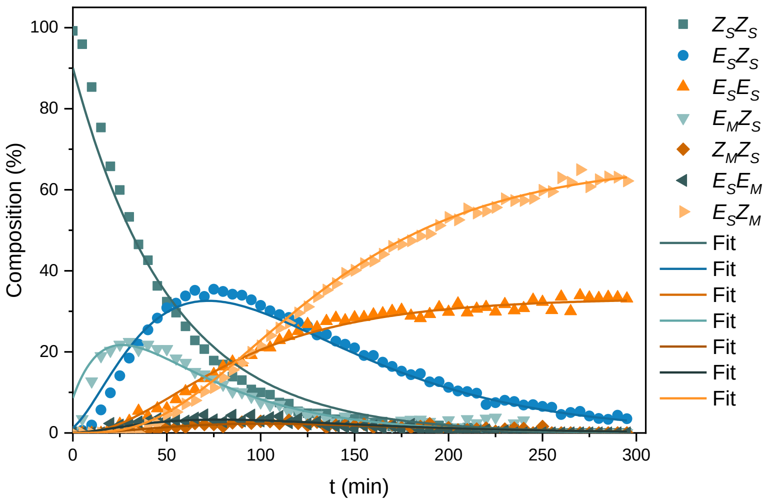

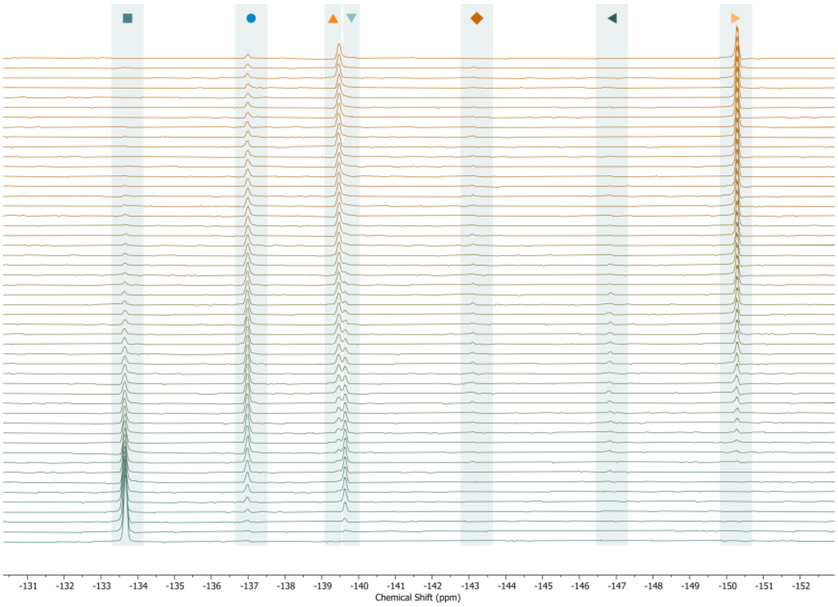

Figure S24. Proposed mechanism with rate constants (top). Kinetic model fitting of (*Z_SZ_S*)-**1** upon irradiation to PSS with 455 nm light at -85 °C with COPASI (middle). The fit is based on the data from the NMR irradiation experiment (Figure S15, top left). Stacked NMR data (bottom).

Relaxation kinetics of E_SZ_M and E_MZ_M at -70 °C

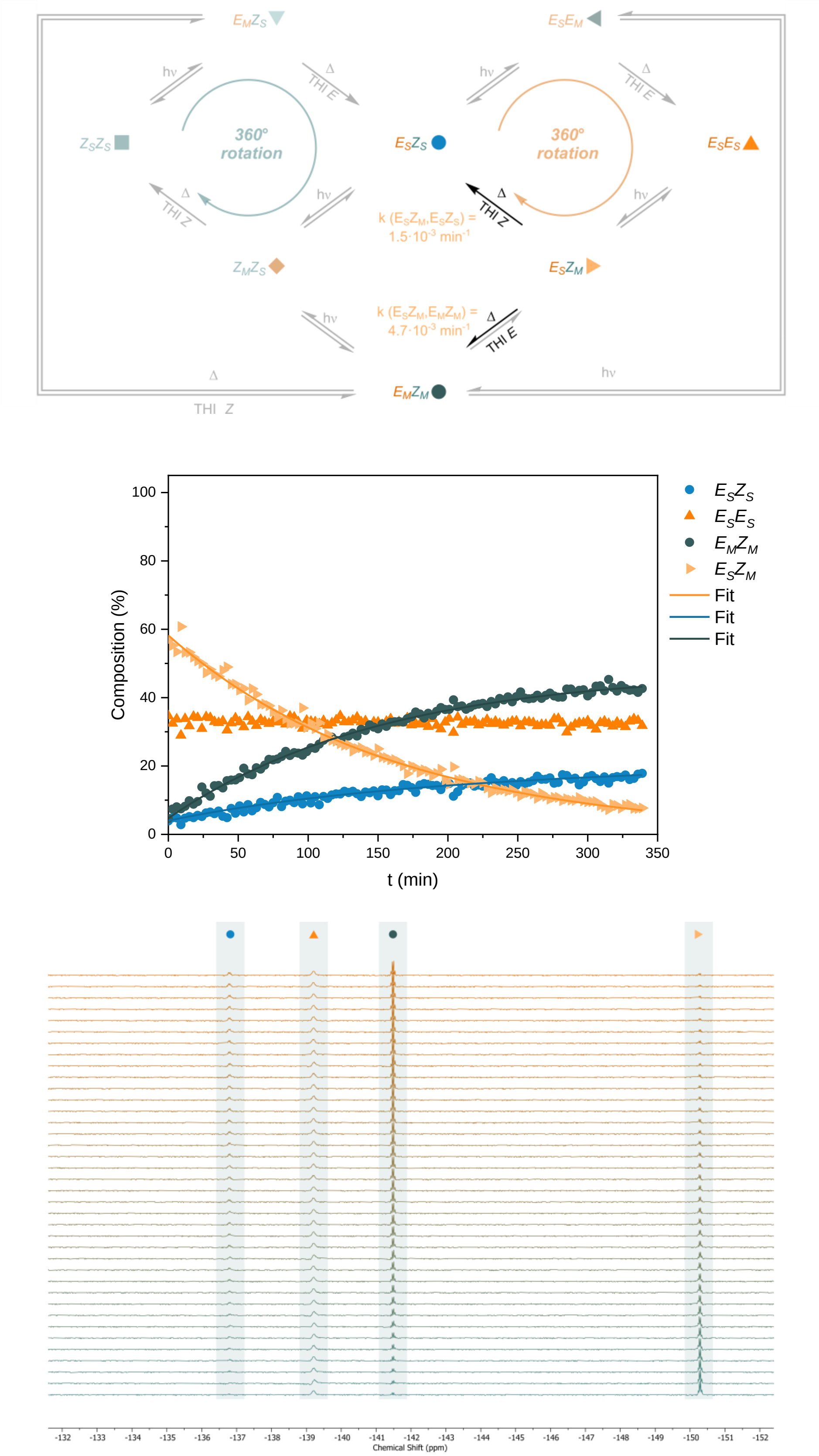

Figure S25. Proposed mechanism with rate constants (top). Kinetic model fitting of thermal relaxation pre-irradiated (Z_SZ_S)-**1** (PSS with 455 nm at -85 °C) at -70 °C with COPASI (middle). The fit is based on the data from the NMR thermal relaxation experiment (Figure S15, top right). Stacked NMR data (bottom).

Relaxation kinetics of E_SE_M at -85 °C

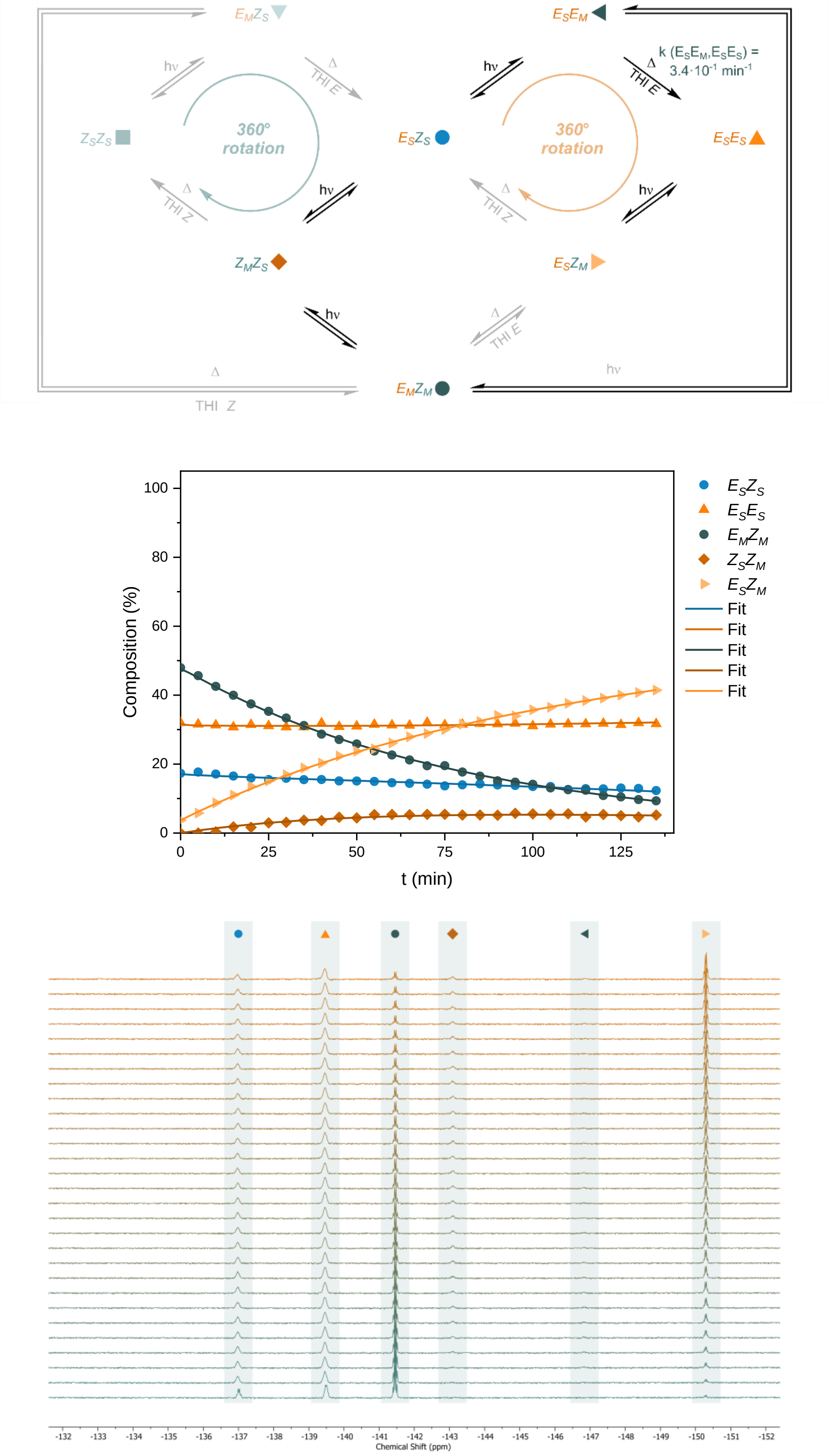

Figure S26. Proposed mechanism with rate constants (top). Kinetic model fitting of a sample enriched with (E_MZ_M)-**1** upon irradiation to PSS with 455 nm light at -85 °C with COPASI (middle). The fit is based on the data from the NMR irradiation experiment (Figure S19). Stacked NMR data (bottom).

Relaxation kinetics of E_MZ_S at -85 °C

The kinetics of THI_E (E_MZ_S,E_SZ_S) were analysed using the traces of (E_MZ_S)-**1** and (E_SZ_S)-**1** in the thermal decay of pre-irradiated (Z_SZ_S)-**1** (455 nm for 20 min at -85 °C) at -85 °C (Figure S18). These results are summarised in Figure S27.

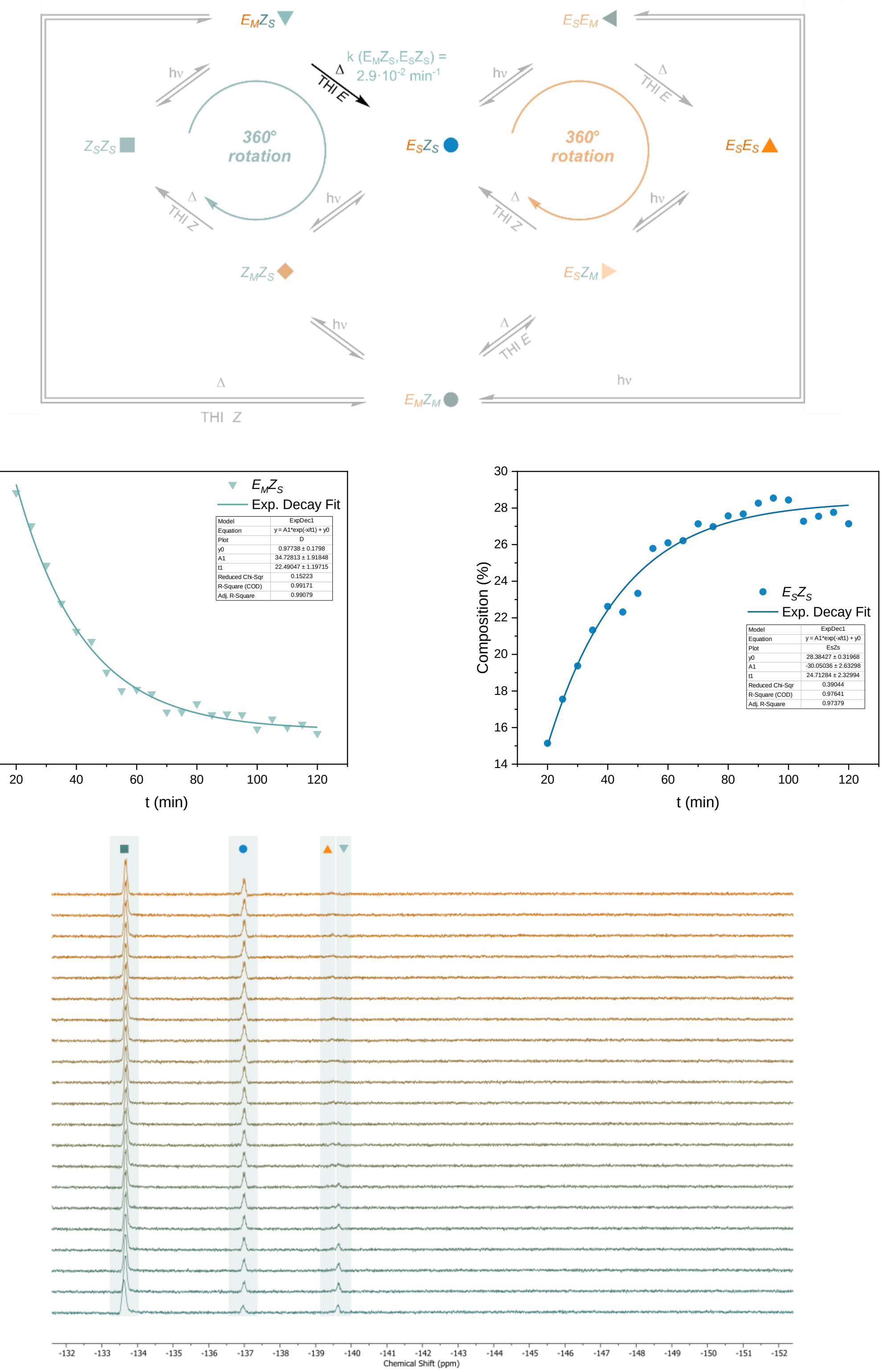

Figure S27. Proposed mechanism with rate constants (top). The traces of (E_MZ_S)-**1** (left middle) and (E_SZ_S)-**1** (right middle) in the thermal decay of pre-irradiated (Z_SZ_S)-**1** (455 nm for 20 min at -85 °C) at -85 °C versus time. The half-life ($t_{1/2}$) was obtained by fitting the data to the mono-exponential decay equation: $A = y_0 + A_1e^{-t/(t1/2)}$ using Origin software. Stacked NMR data (bottom).

Relaxation kinetics of E_MZ_M at -30 °C

The kinetics of $E_MZ_M \rightarrow E_SZ_S$ were analysed using the traces of (E_MZ_M)-**1** and (E_SZ_S)-**1** in the thermal decay of pre-irradiated (Z_SE_S)-**1** (PSS with 455 nm at -85 °C) at -30 °C (Figure S17). These results are summarised in Figure S28. Under the experimental conditions, no re-population of (E_SZ_M)-**1** was observed. This could be explained by the fact that the equilibrium between (E_MZ_M)-**1** and (E_SZ_M)-**1** is strongly favoured towards (E_MZ_M)-**1**. Hence, (E_SZ_M)-**1** appears to be stable up to -45 °C despite the interconversion of (E_MZ_M)-**1** and (E_SZ_M)-**1** being already kinetically accessible at lower temperatures (see the calculated barriers in Figure S33). Alternatively, the reverse CO-flip is the rate-determining step and hence the faster kinetics of the subsequent THI_Z explain the absence of (E_SZ_M)-**1** during the full relaxation process from (E_MZ_M)-**1** to (E_SZ_S)-**1**.

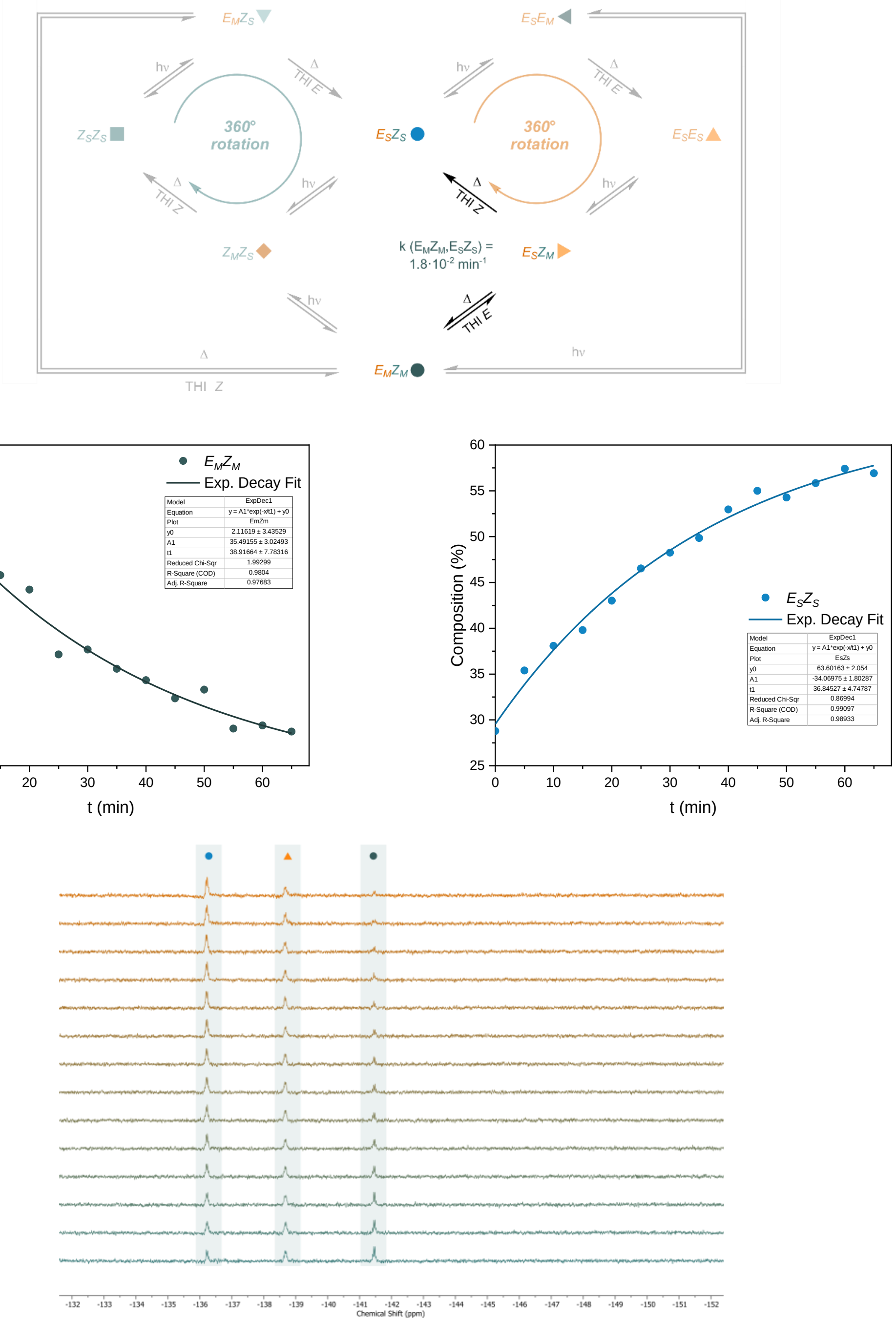

Figure S28. Proposed mechanism with rate constants (top). The trace of (E_MZ_M)-**1** (middle left) and (E_SZ_S)-**1** (middle right) in the thermal decay of pre-irradiated (Z_SE_S)-**1** (PSS with 455 nm at -85 °C) at -30 °C versus time. The half-life ($t_{1/2}$) was obtained by fitting the data to the mono-exponential decay equation: $A = y_0 + A_1e^{-t/(t1/2)}$ using Origin software. Stacked NMR data (bottom).

5. Single Crystal XRD

Motors ($Z_S Z_S$)-**1** and ($E_S Z_S$)-**1** were crystallised by vapor diffusion of MeOH to a solution of the dissolved compound in $CHCl_3$. A single crystal was mounted on a cryoloop and analysed on a Bruker-AXS D8 Venture diffractometer. The crystal was kept under ambient conditions during data collection. The Bruker APEX4 software suite was used for data collection and processing and the structure was solved using SHELXT.[4] Refinement was performed using SHELXL[5] in the OLEX2 software package.[6] No A- or B-level alerts were raised by CheckCIF for the fully refined structure

Third-generation motors can possess two nondegenerate isomers depending on the substation pattern of the pseudo-asymmetric centre. In line with previous results[1,7] and our NMR data, motor **1** was obtained with a single configuration at the pseudo-asymmetric centre. This can be explained by the sufficient size difference between the methyl and fluorine substituents. The larger methyl group prefers a pseudo-axial orientation, which avoids the pinching rotor moieties associated to the pseudo-equatorial position. This conformation with a pseudo-equatorial positioned fluorine atom can be assigned as (*r*)-**1**.

Z_SZ_S *isomer*

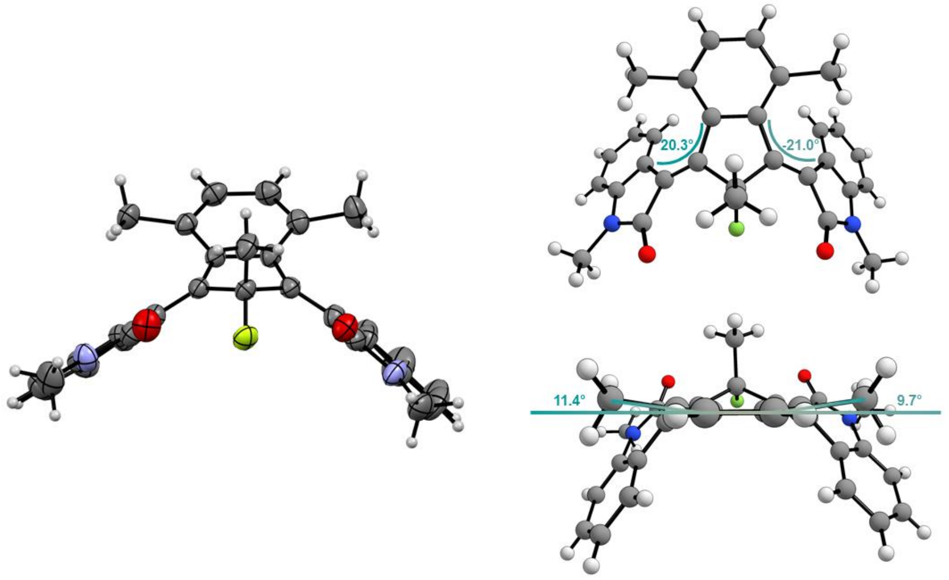

Figure S29. X-ray structure of (Z_SZ_S)-**1**, front view (left). Ellipsoids are set at 50% probability. RMSD = 0.34 Å (Model // r^{2}SCAN-3c CPCM(CH_2Cl_2)). Top view (right top) with the measured dihedral angles around the central C-C double bonds indicated. Rear view (right bottom) with the deviation of the methyl groups from the core's planarity indicated.

E_SZ_S *isomer*

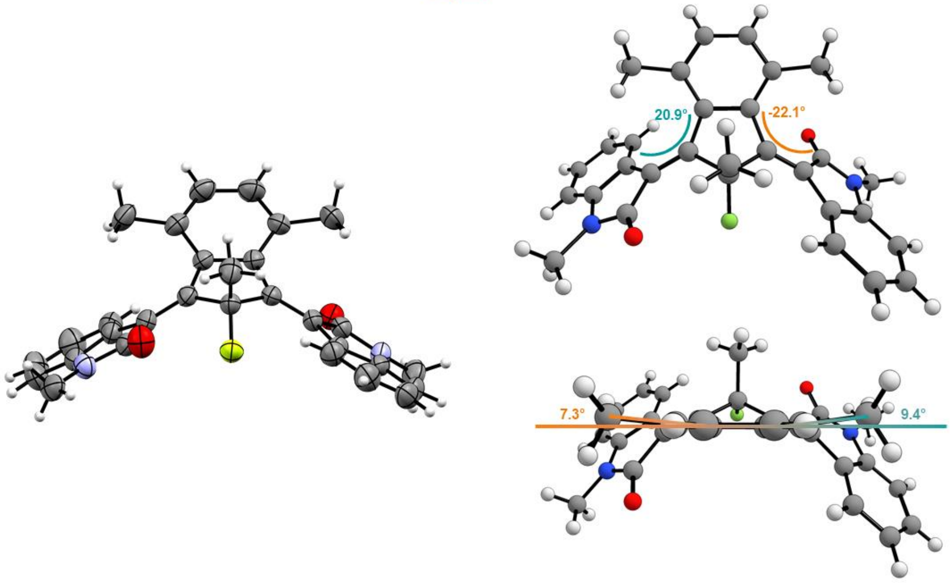

Figure S30. X-ray structure of (E_SZ_S)-**1**, front view (left). Ellipsoids are set at 50% probability. RMSD = 0.19 Å (Model // r^{2}SCAN-3c CPCM(CH_2Cl_2)). Top view (right top) with the measured dihedral angles around the central C-C double bonds indicated. Rear view (right bottom) with the deviation of the methyl groups from the core's planarity indicated.

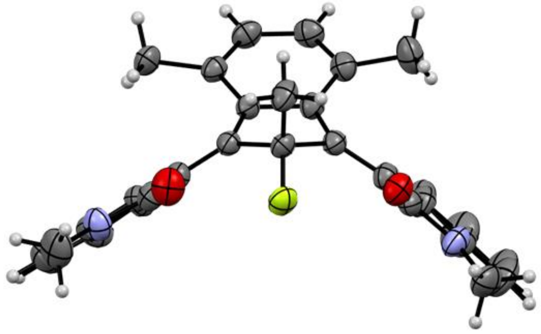

*Table S2. Crystal data and structure refinement for (Z_SZ_S)-**1**.*

Identification code	mo_CNS_CLFB_24_0ma_a
Empirical formula	$C_{30}H_{25}FN_2O_2$
Formula weight	464.52
Temperature/K	293
Crystal system	monoclinic
Space group	$P2_1/n$
a/Å	11.9901(13)
b/Å	12.4565(13)
c/Å	16.0759(16)
α/°	90
β/°	92.363(5)
γ/°	90
Volume/Å^3	2399.0(4)
Z	4
ρ_{calc}g/cm^3	1.286
μ/mm^{-1}	0.086
F(000)	976.0
Crystal size/mm^3	0.384 × 0.281 × 0.079
Radiation	MoKα (λ = 0.71073)
2Θ range for data collection/°	5.29 to 60.108
Index ranges	$-16 \leq h \leq 16$, $-17 \leq k \leq 17$, $-22 \leq l \leq 22$
Reflections collected	144876
Independent reflections	7007 [R_{int} = 0.1462, R_{sigma} = 0.0447]
Data/restraints/parameters	7007/0/321
Goodness-of-fit on F^2	1.045
Final R indexes [I>=2σ (I)]	R_1 = 0.0630, wR_2 = 0.1225
Final R indexes [all data]	R_1 = 0.1089, wR_2 = 0.1489
Largest diff. peak/hole / e Å^{-3}	0.26/-0.21

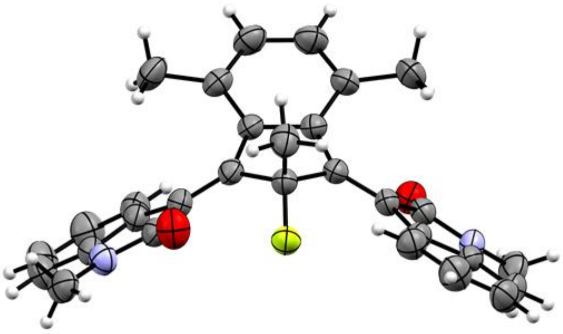

*Table S3. Crystal data and structure refinement for (E_SZ_S)-**1**.*

Identification code	mo_CNS_CLFB_0m_a
Empirical formula	$C_{30}H_{25}FN_2O_2$
Formula weight	464.52
Temperature/K	293
Crystal system	triclinic
Space group	P-1
a/Å	8.2988(10)
b/Å	12.1964(16)
c/Å	12.3517(13)
α/°	86.163(5)
β/°	77.055(5)
γ/°	73.904(6)
Volume/Å^3	1170.6(2)
Z	2
ρ_{calc}g/cm^3	1.318
μ/mm^{-1}	0.088
F(000)	488.0
Crystal size/mm^3	0.92 × 0.21 × 0.186
Radiation	MoKα (λ = 0.71073)
2Θ range for data collection/°	4.864 to 59.15
Index ranges	$-11 \leq h \leq 11$, $-16 \leq k \leq 16$, $-17 \leq l \leq 17$
Reflections collected	76653
Independent reflections	6562 [R_{int} = 0.1362, R_{sigma} = 0.0609]
Data/restraints/parameters	6562/0/321
Goodness-of-fit on F^2	1.062
Final R indexes [I>=2σ (I)]	R_1 = 0.0801, wR_2 = 0.1879
Final R indexes [all data]	R_1 = 0.1587, wR_2 = 0.2468
Largest diff. peak/hole / e Å^{-3}	0.56/-0.26

6. Computational Details

Computational analysis was used to investigate the thermal steps of the complex motor operation mechanism. All calculations were done using the ORCA 5.0.4 software package.[8] Geometry optimisations were performed at the r^{2}SCAN-3c level of theory.[9] Solvation was included in all calculations using the CPCM[10] models with parameters for CH_2Cl_2 as the solvent. The stationary points were confirmed using frequency calculations and evaluation of the number of imaginary frequencies (0 for minima and 1 for transition states). The XYZ coordinates of all optimised structures can be found in an additional file.

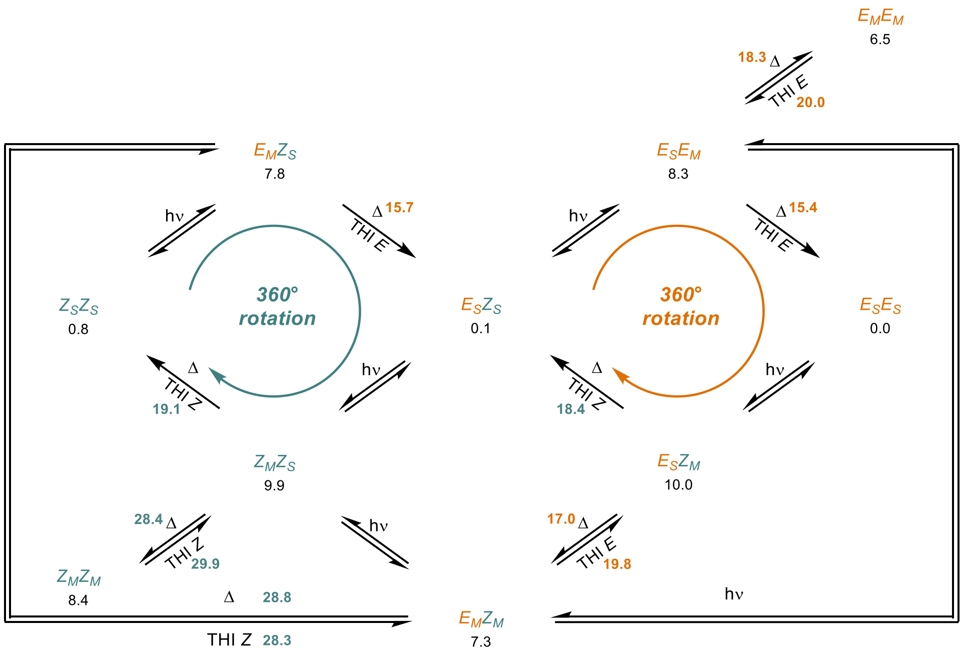

Figure S31. Rotation mechanism of **1** with calculated energies (shown in black) and activation barriers (shown in bold and colour: orange for THI$_E$ and blue for THI$_Z$). For transition states, the energy barriers are referenced to the associated initial state. For all intermediates, the energies are referenced to (E_SE_S)-**1**. Energies and activation barriers were calculated at the r^{2}SCAN-3c CPCM(CH_2Cl_2) level of theory and are shown in kcal mol^{-1}.

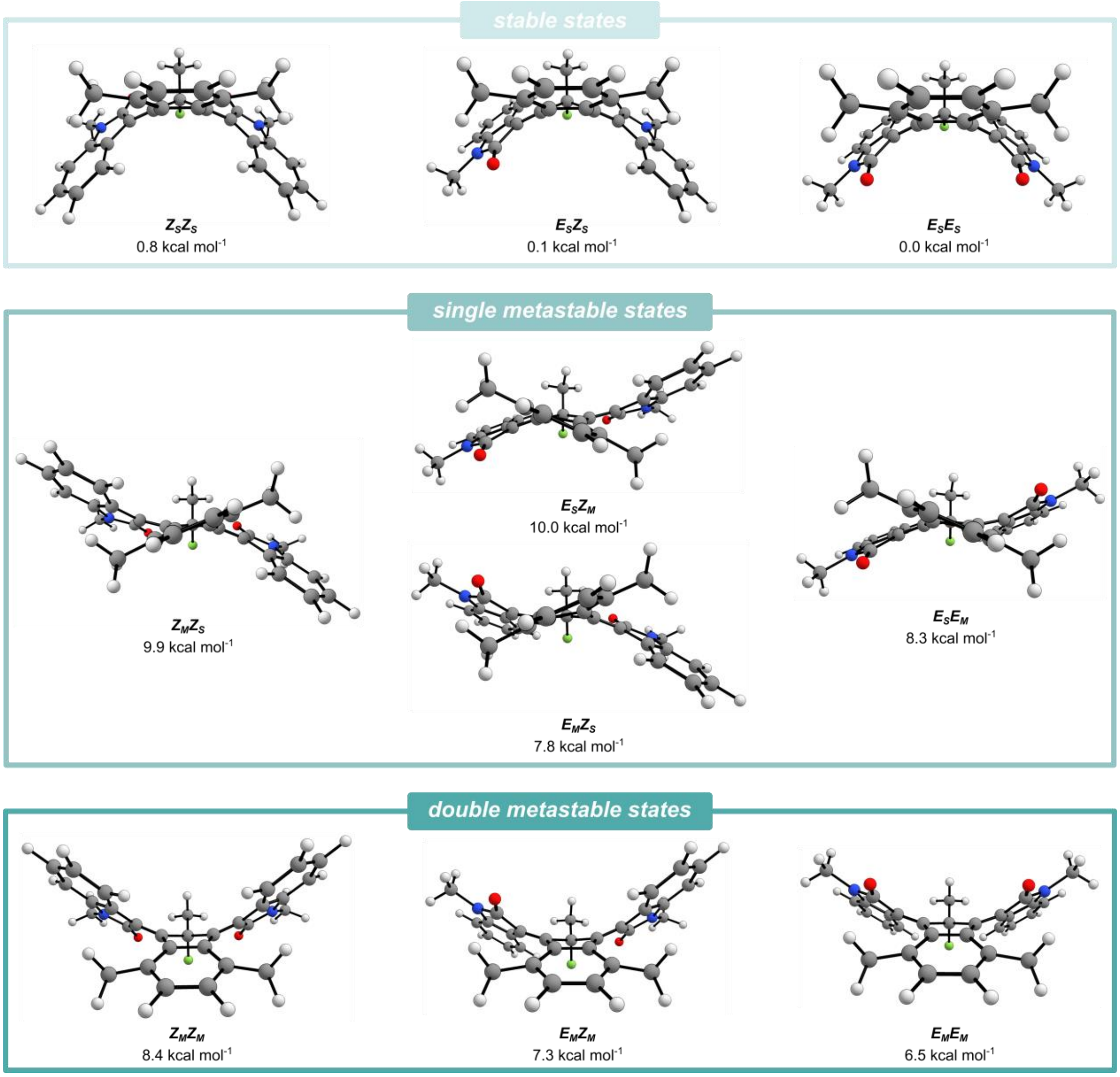

Figure S32. Optimised structures of all intermediates categorised by number of rotors in a metastable geometry (stable = zero, single metastable = one, double metastable = two). The energies were calculated at the r^{2}SCAN-3c CPCM(CH_2Cl_2)) level of theory and are referenced to (E_SE_S)-**1**.

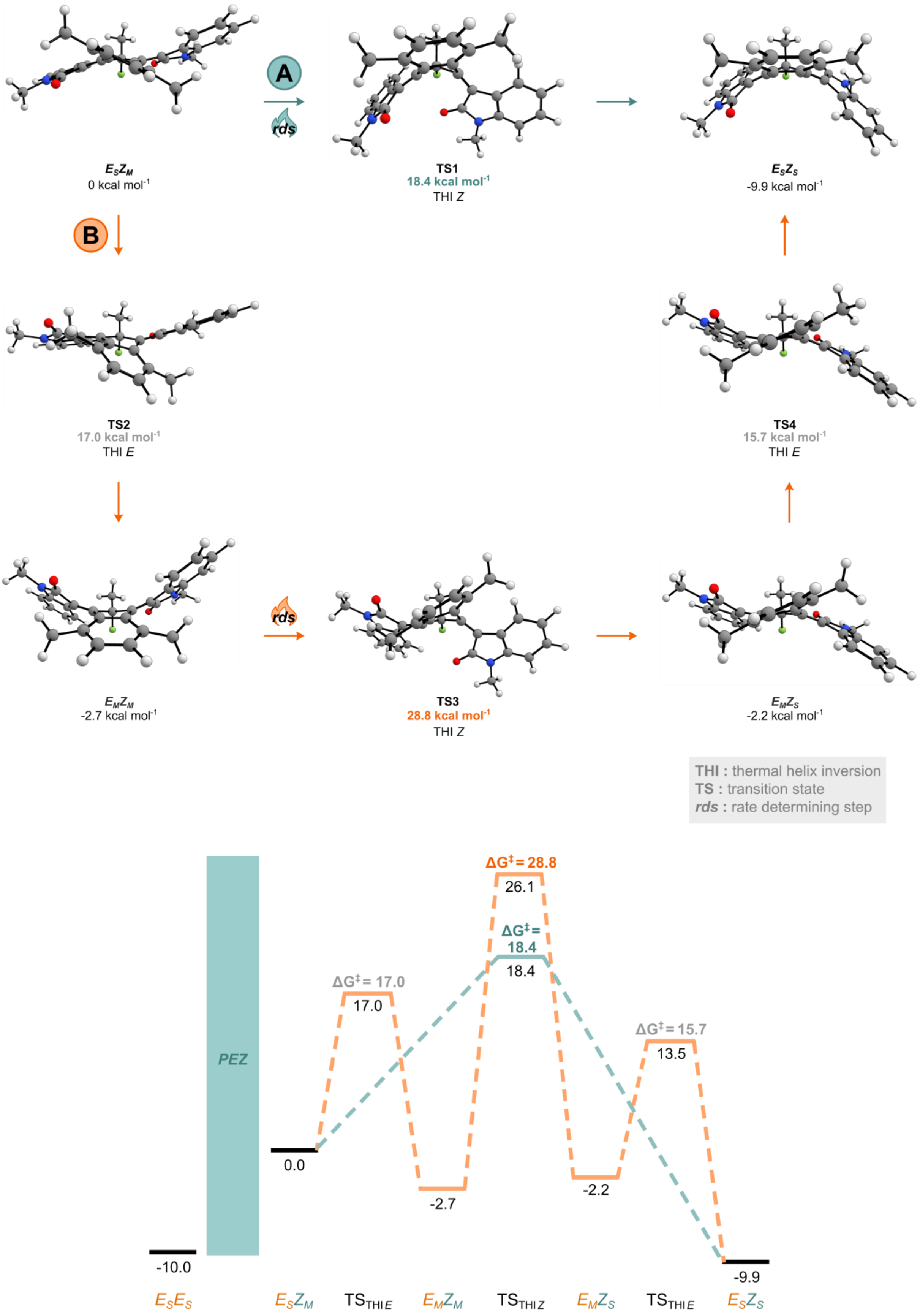

Figure S33. Thermal conversion of (E_SZ_M)-**1** to (E_SZ_S)-**1** via pathways A (THI_Z shown in orange) and B (a series of CO-flip (THI_E) and THI_Z and THI_E shown in blue). For transition states, the activation barriers are referenced to the associated initial state. Energies (black) and activation barriers (bold coloured) were calculated at the r^{2}SCAN-3c CPCM(CH_2Cl_2)) level of theory and are shown in kcal mol^{-1}.

Table S4. Relative energies of motor **1** calculated at the r^{2}SCAN-3c CPCM(CH_2Cl_2)) level of theory. The energies are referenced to (E_SE_S)-**1**.

T (K)<br>*T (°C)*<br>Motor isomer	298.15<br>*25*	243.15<br>*-30*	218.15<br>*-55*	203.15<br>*-70*	188.15<br>*-85*	183.15<br>*-90*	163.15 -<br>*110*
	Relative energy (kcal mol^{-1})						
E_SE_S	0.0	0.0	0.0	0.0	0.0	0.0	0.0
E_SZ_S	0.1	0.1	0.1	0.1	0.1	0.1	0.1
Z_SZ_S	0.8	0.8	0.8	0.9	0.9	0.9	0.9
E_SE_M	8.3	8.3	8.3	8.3	8.3	8.3	8.3
E_MZ_S	7.8	7.8	7.8	7.8	7.8	7.8	7.8
E_SZ_M	10.0	10.0	10.0	10.0	10.0	10.0	10.0
Z_MZ_S	9.9	9.9	9.9	9.9	10.0	10.0	10.0
E_ME_M	6.5	6.5	6.4	6.4	6.4	6.4	6.4
E_MZ_M	7.3	7.2	7.2	7.2	7.2	7.2	7.2
Z_MZ_M	8.4	8.4	8.4	8.4	8.4	8.4	8.4
**TS1**<br>THI_Z<br>(E_SZ_M,E_SZ_S)	28.5	28.3	28.3	28.2	28.2	28.2	28.1
**TS2**<br>THI_E<br>(E_SZ_M,E_MZ_M)	27.1	26.9	26.8	26.7	26.7	26.7	26.6
**TS3**<br>THI_Z<br>(E_MZ_M,E_MZ_S)	36.1	36.0	36.0	36.0	35.9	35.9	35.9
**TS4**<br>THI_E<br>(E_MZ_S,E_SZ_S)	23.5	23.3	23.2	23.2	23.2	23.2	23.1
**TS5**<br>THI_E<br>(E_ME_S,E_SE_S)	23.5	23.5	23.4	23.4	23.3	23.3	23.3
**TS6**<br>THI_Z<br>(Z_SZ_M,Z_SZ_S)	29.0	28.9	28.9	28.9	28.9	28.9	28.8
**TS7**<br>THI_E<br>(E_ME_S,E_ME_M)	26.5	26.3	26.3	26.2	26.2	26.2	26.1
**TS8**<br>THI_Z<br>(Z_SZ_M,Z_MZ_M)	38.3	38.3	38.3	38.2	38.2	38.2	38.2

7. NMR Spectra

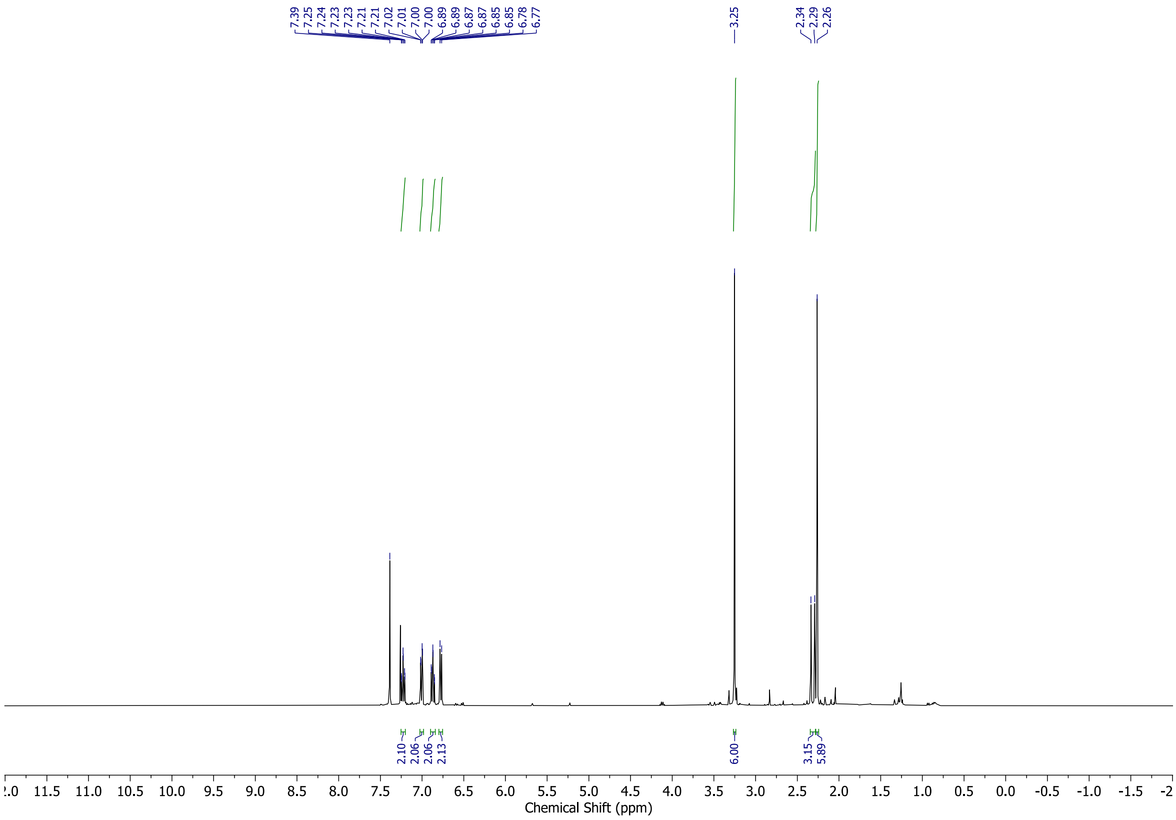

Figure S34. ¹H NMR spectrum of (Z_SZ_S)-**1** ($CDCl_3$, 20 °C).

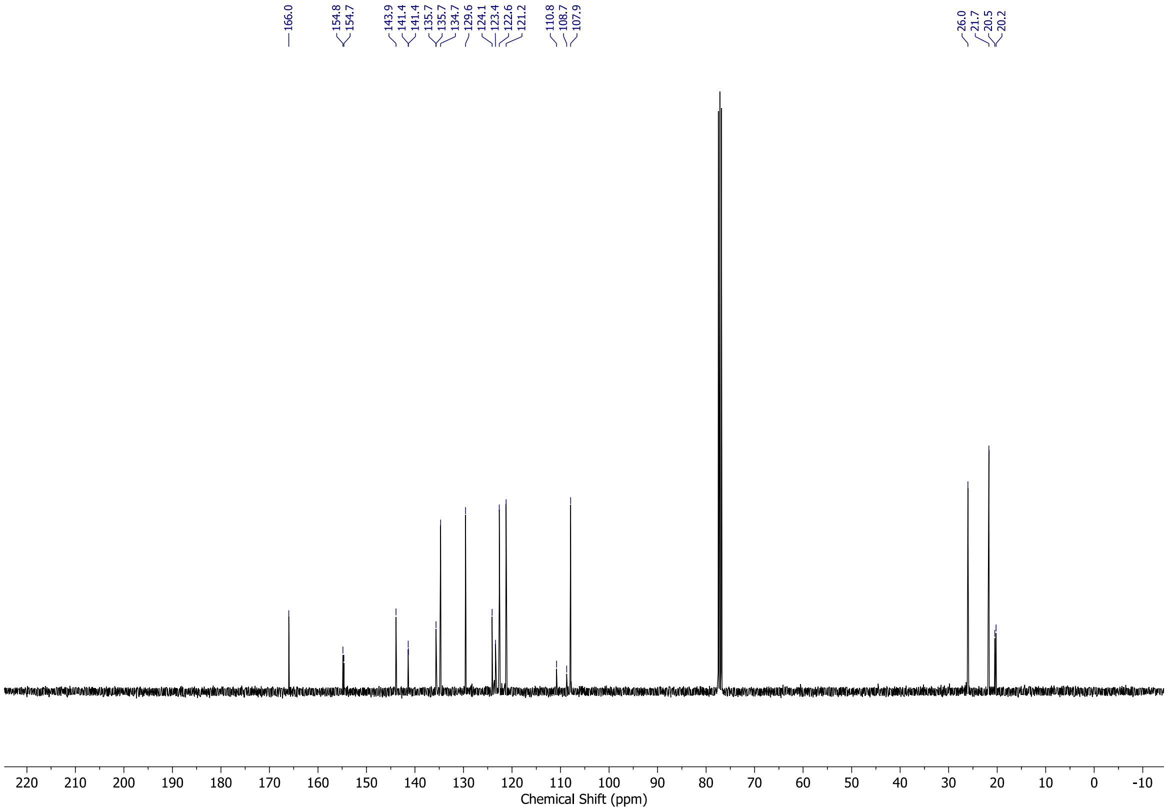

Figure S35. ^{13}C NMR spectrum of (Z_SZ_S)-**1** ($CDCl_3$, 20 °C).

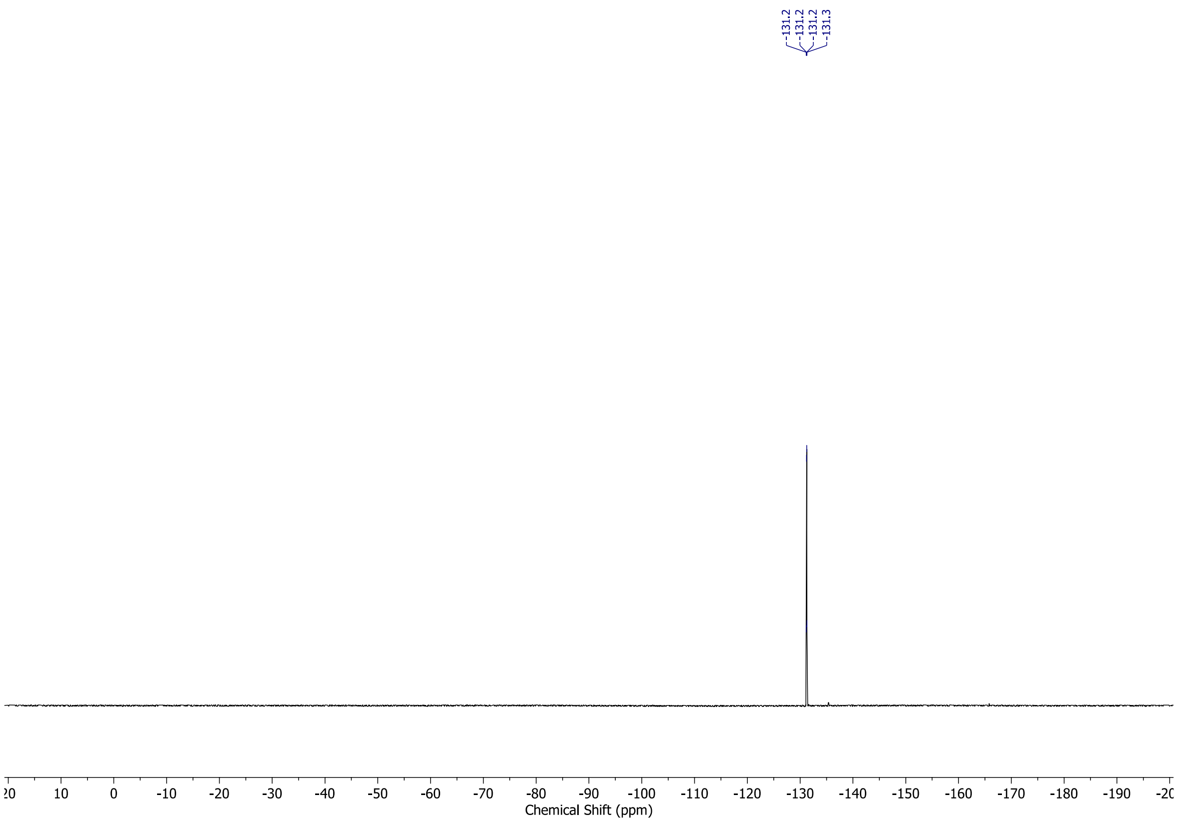

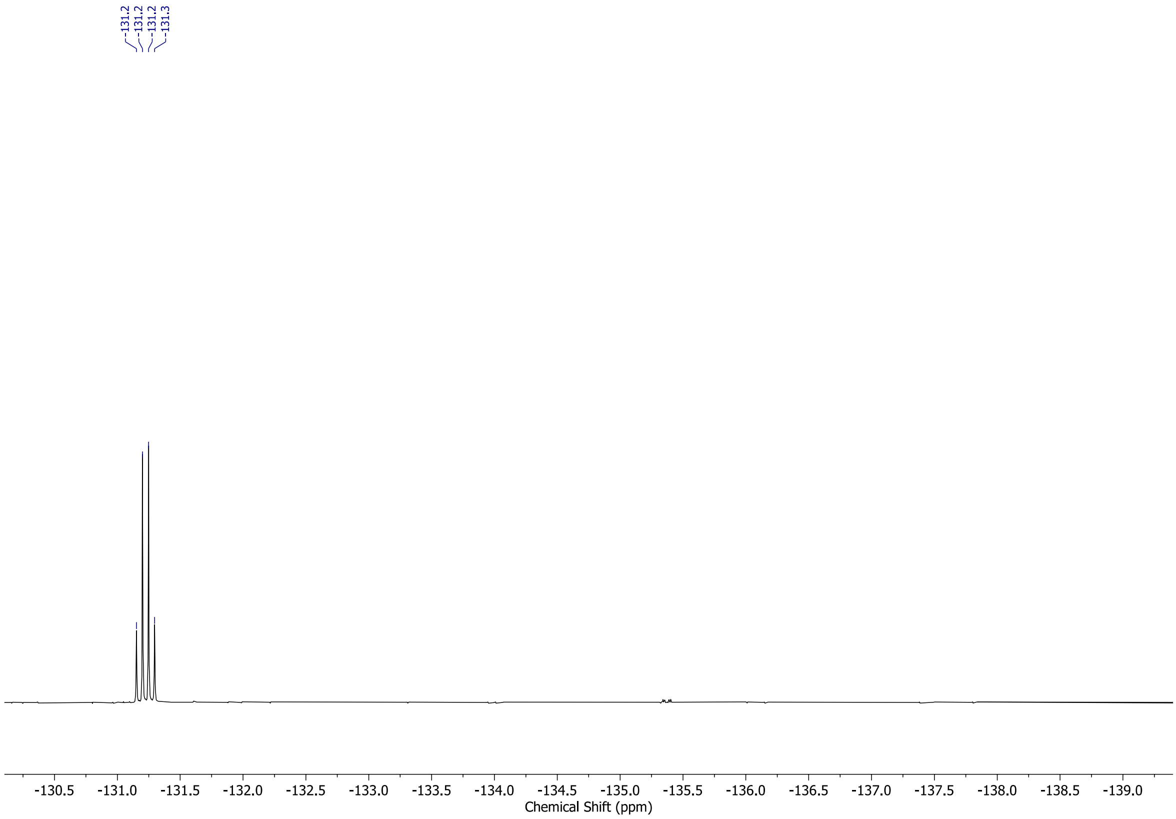

Figure S36. ^{19}F NMR spectrum of (Z_SZ_S)-**1** ($CDCl_3$, 20 °C, full (top) and zoomed (bottom)).

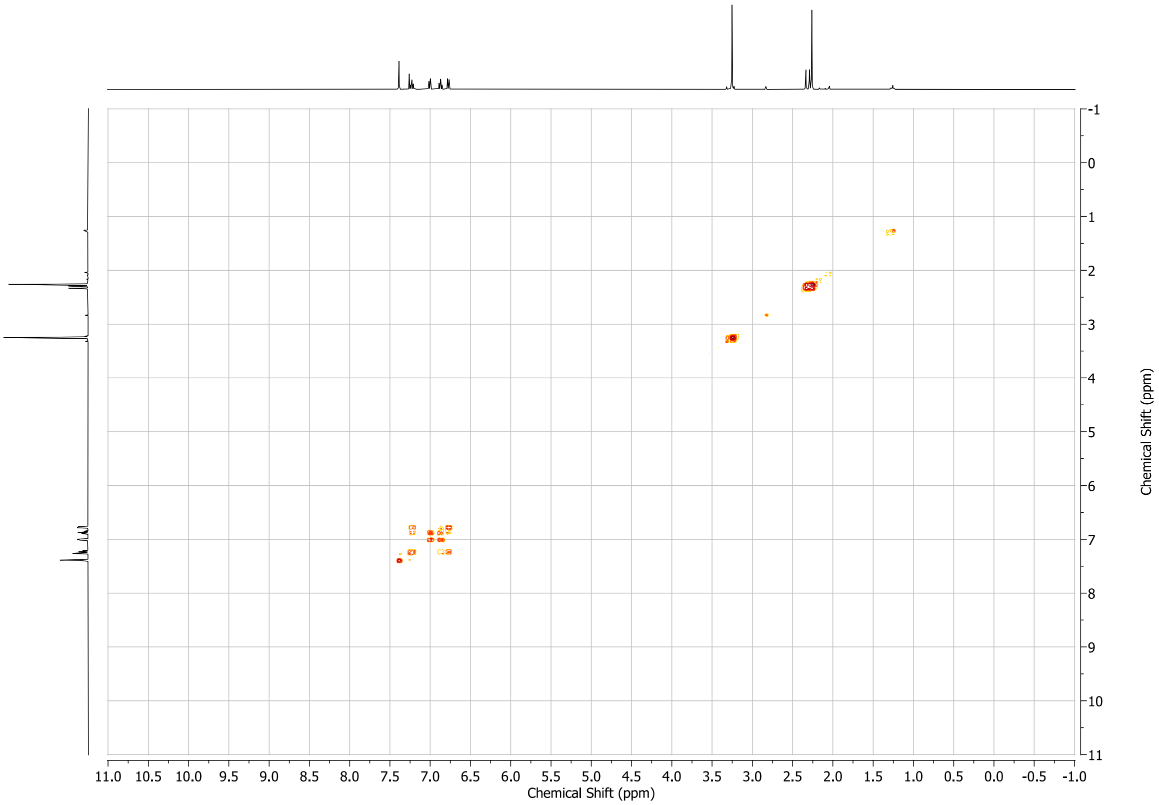

Figure S37. COSY NMR spectrum of (*Z*$_S$*Z*$_S$)-**1** ($CDCl_3$, 20 °C).

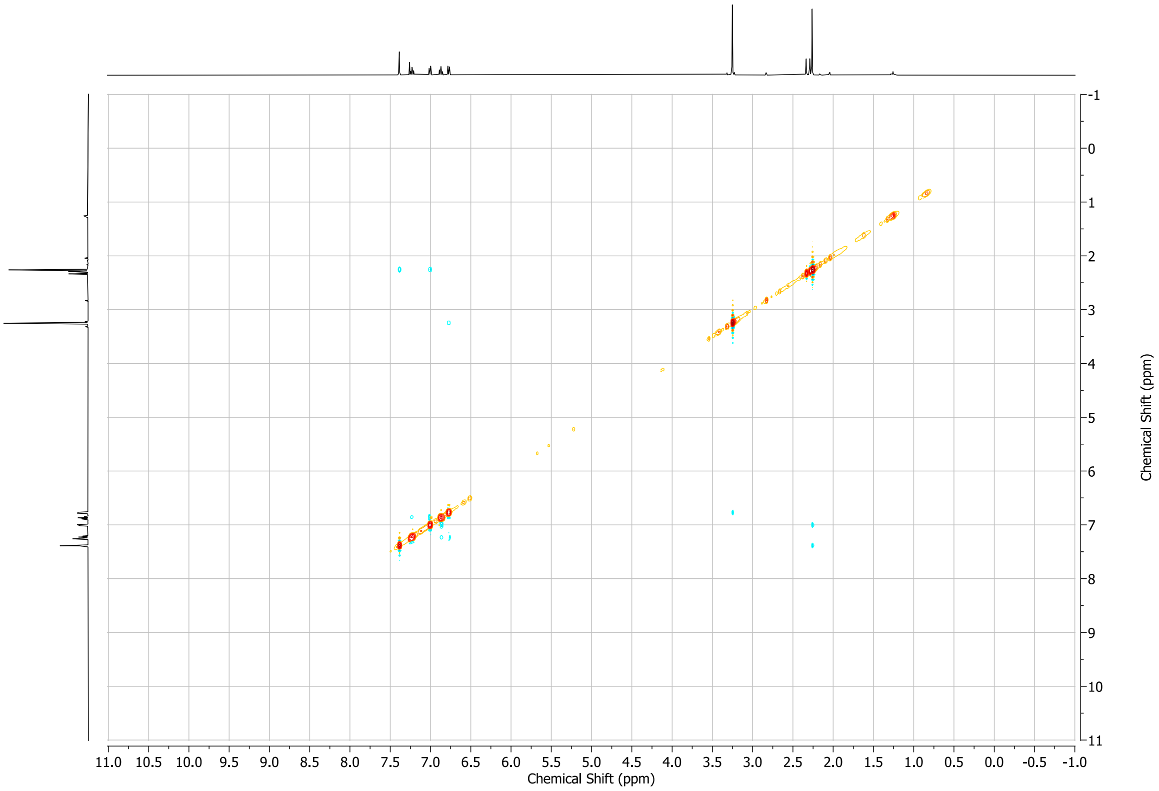

Figure S38. NOESY NMR spectrum of (*Z*$_S$*Z*$_S$)-**1** ($CDCl_3$, 20 °C).

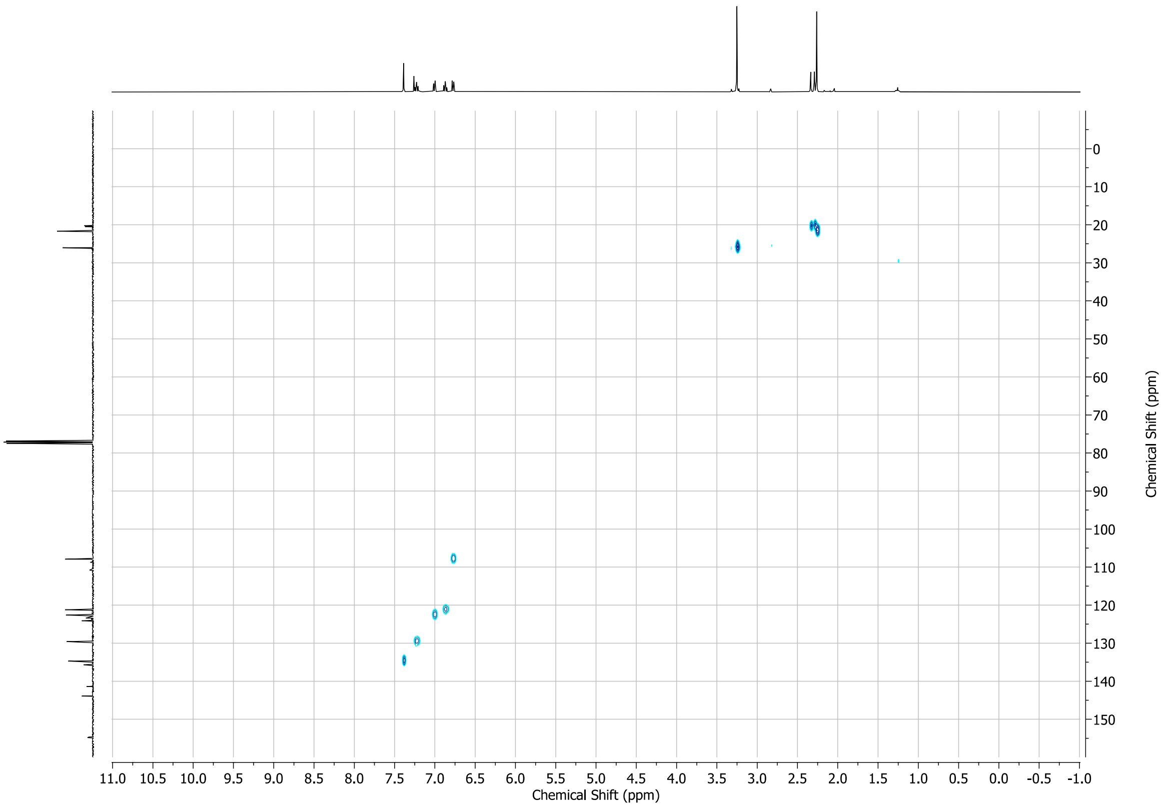

Figure S39. HSQC NMR spectrum of (Z_SZ_S)-**1** ($CDCl_3$, 20 °C).

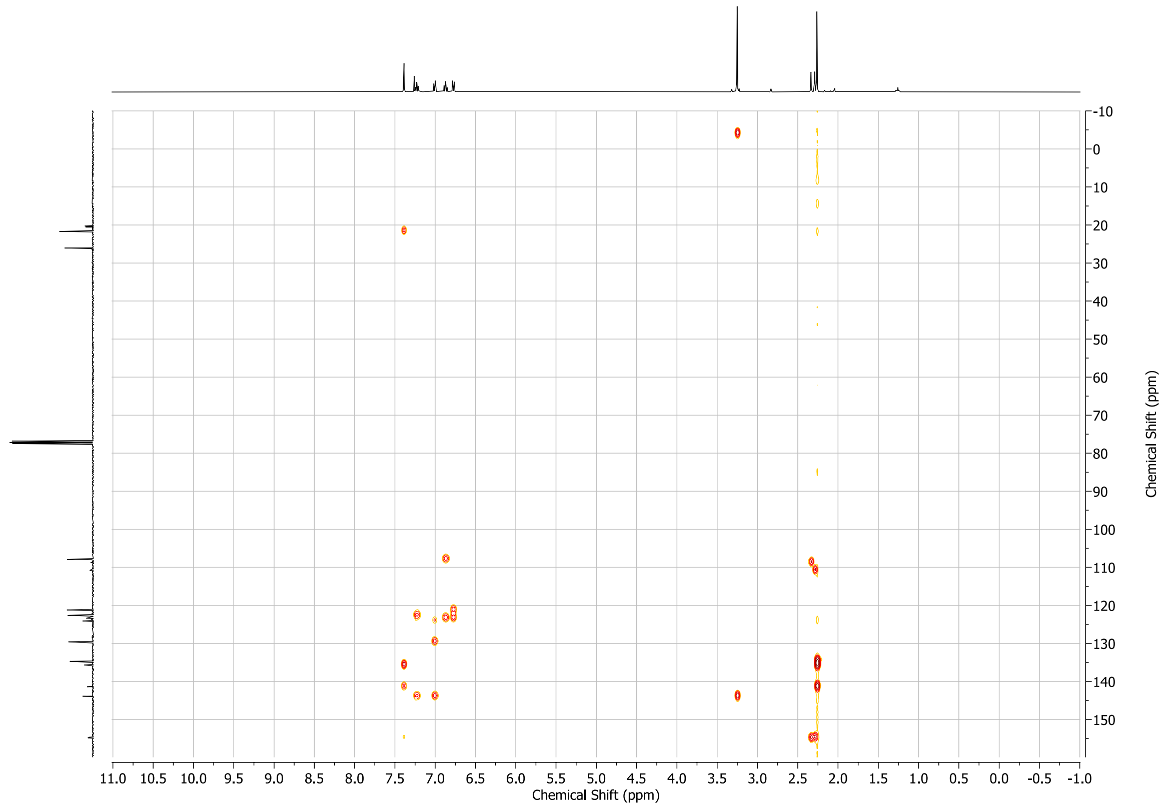

Figure S40. HMBC NMR spectrum of (Z_SZ_S)-**1** ($CDCl_3$, 20 °C).

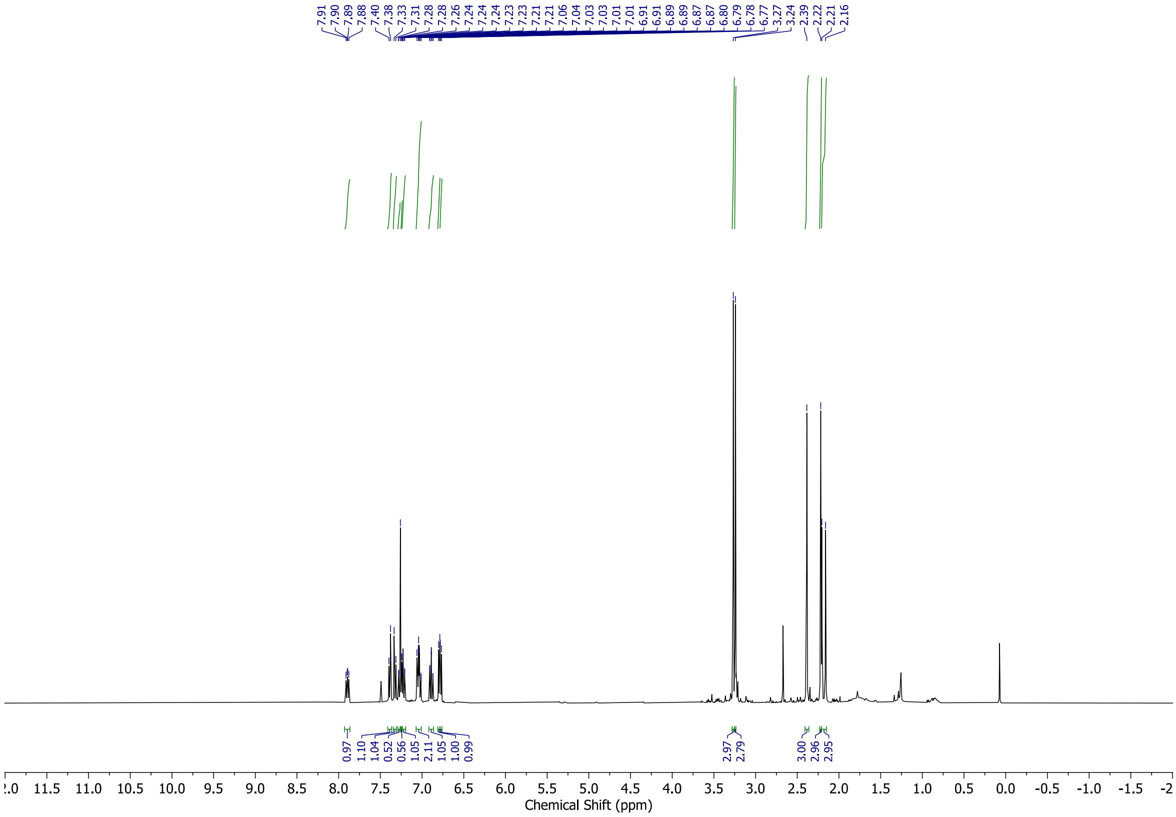

Figure S41. ^{1}H NMR spectrum of (E_SZ_S)-**1** ($CDCl_3$, 20 °C).

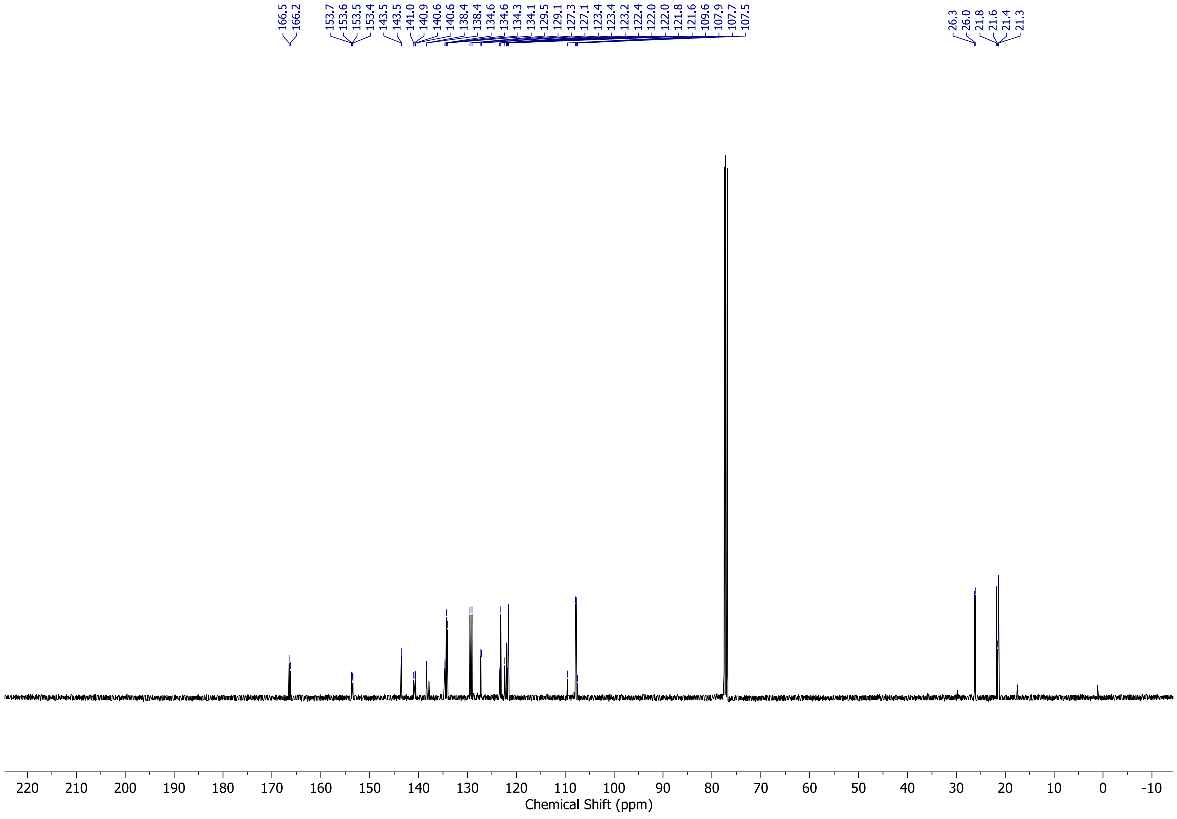

Figure S42. ^{13}C NMR spectrum of (E_SZ_S)-**1** ($CDCl_3$, 20 °C).

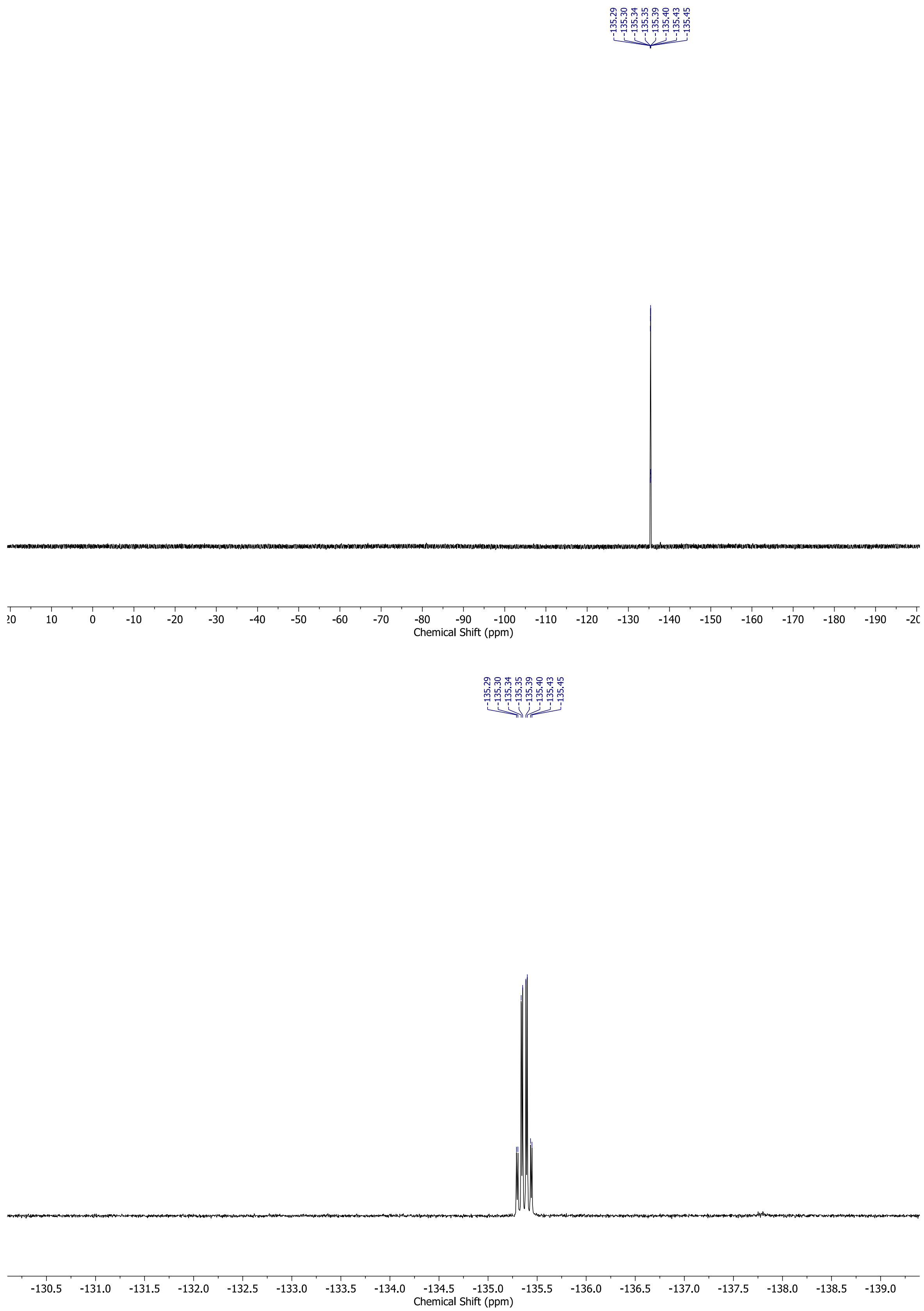

Figure S43. ^{19}F NMR spectrum of (E_SZ_S)-**1** ($CDCl_3$, 20 °C, full (top) and zoomed (bottom)).

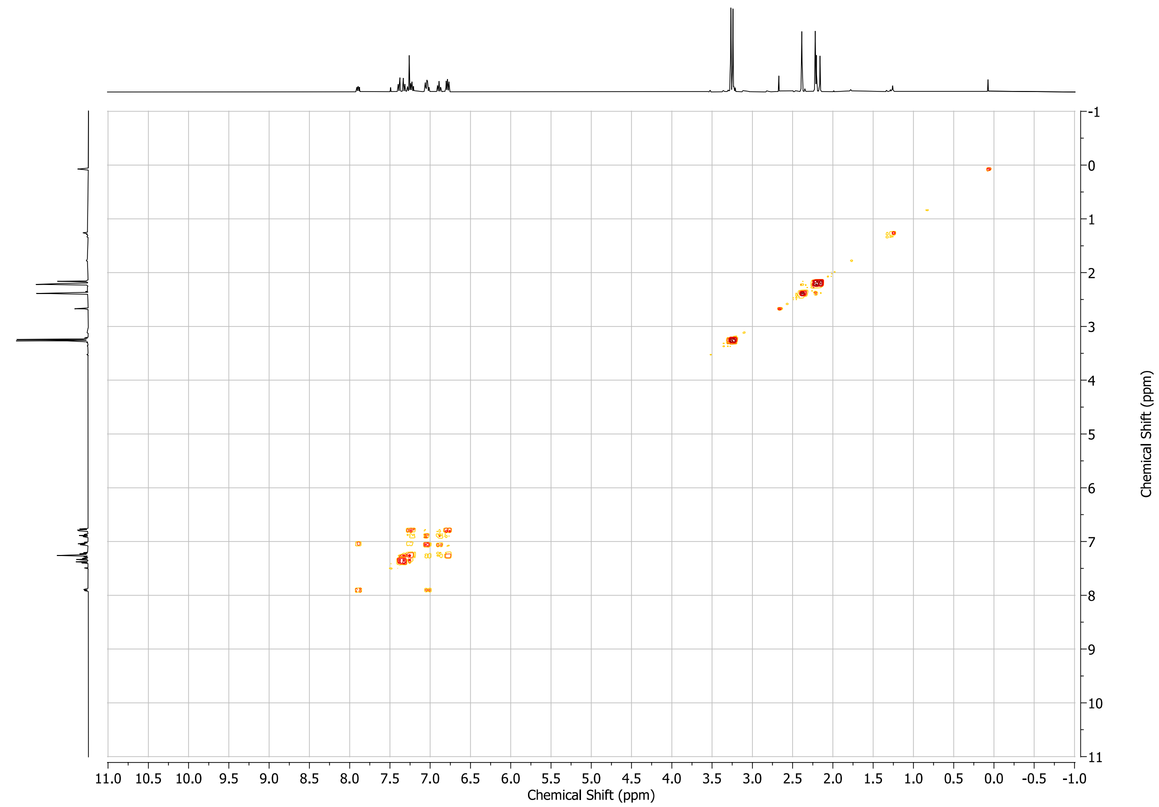

Figure S44. COSY NMR spectrum of (E_SZ_S)-**1** ($CDCl_3$, 20 °C).

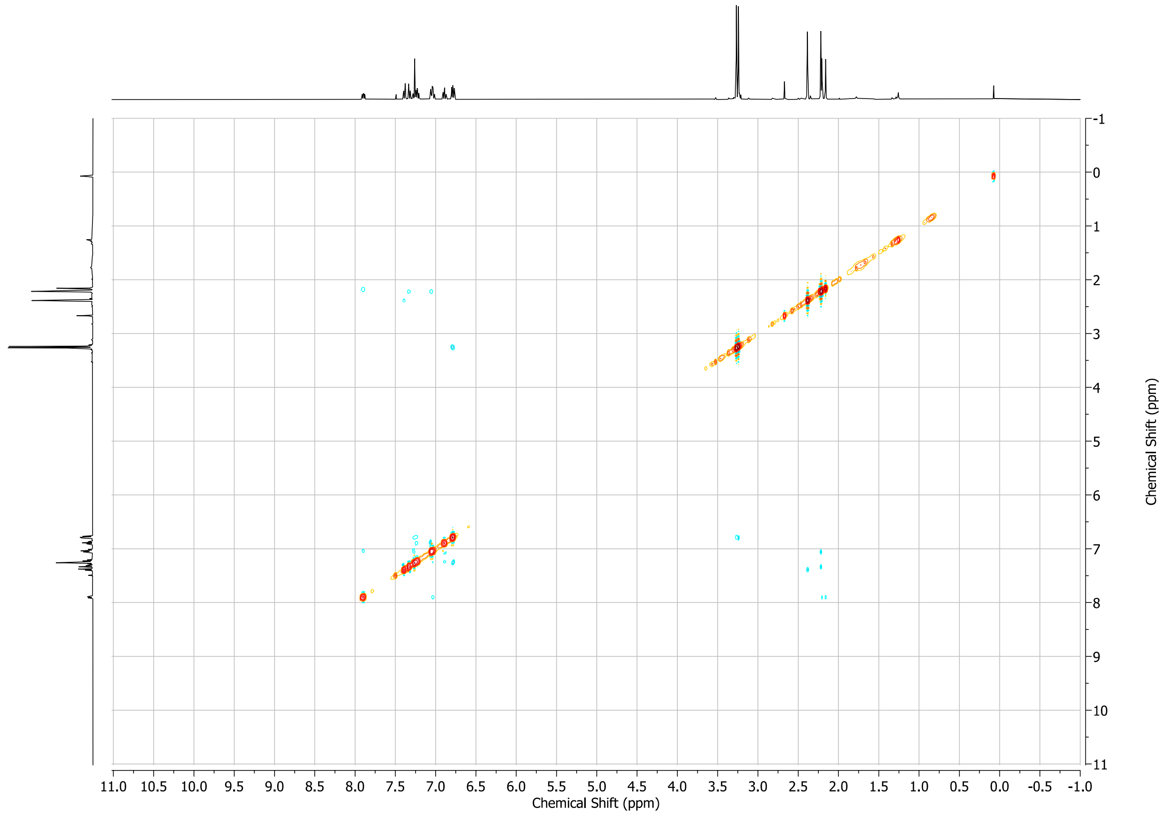

Figure S45. NOESY NMR spectrum of (E_SZ_S)-**1** ($CDCl_3$, 20 °C).

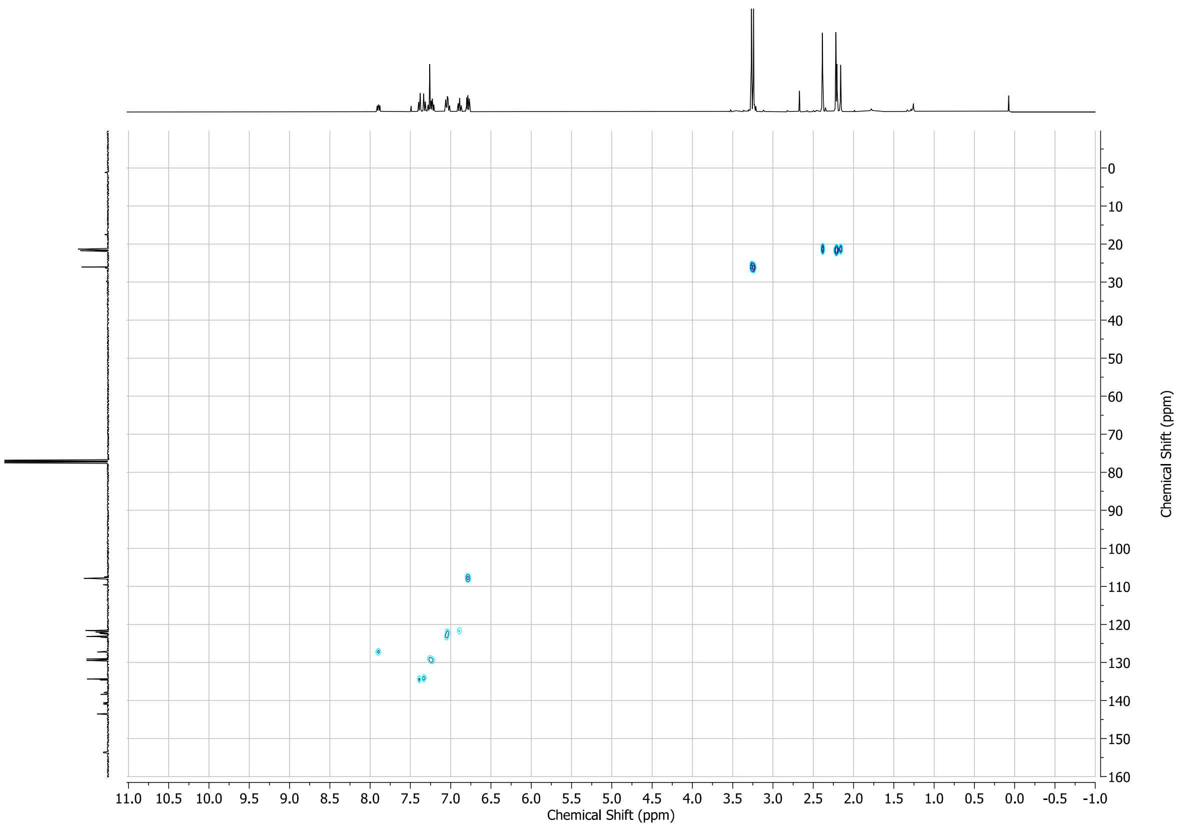

Figure S46. HSQC NMR spectrum of (E_SZ_S)-**1** ($CDCl_3$, 20 °C).

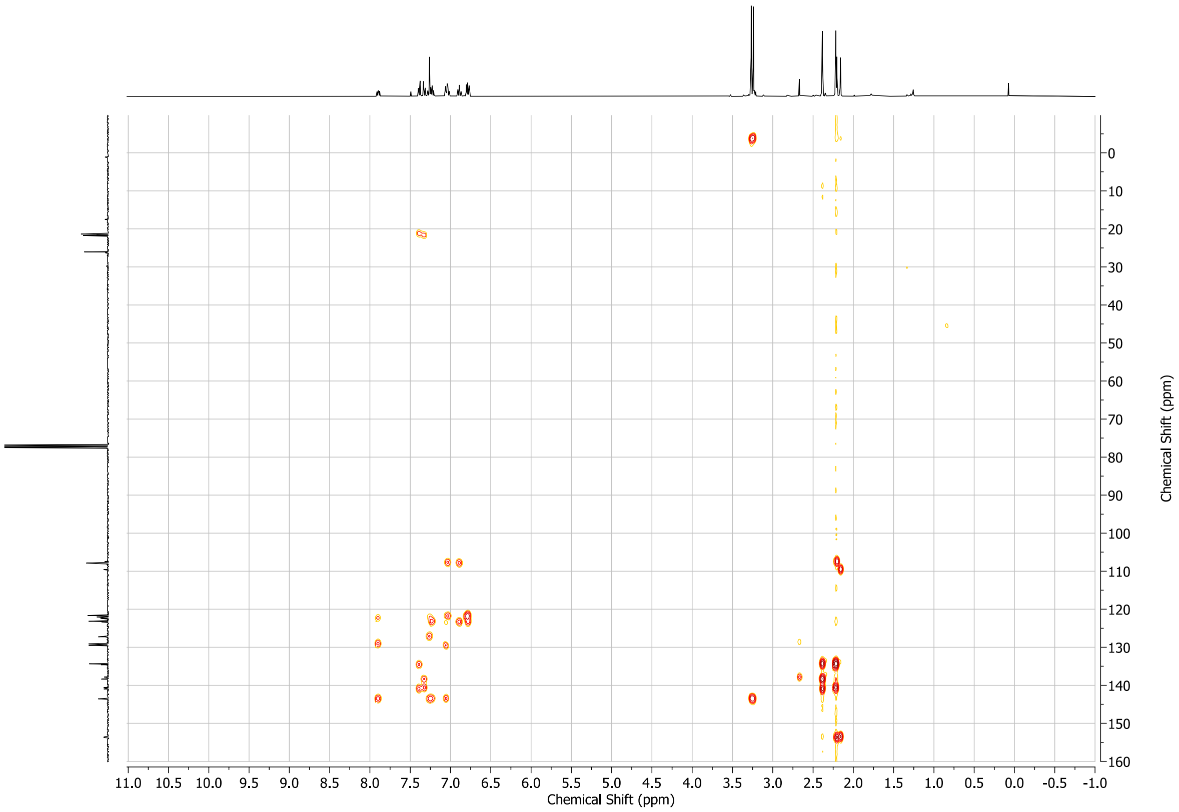

Figure S47. HMBC NMR spectrum of (E_SZ_S)-**1** ($CDCl_3$, 20 °C).

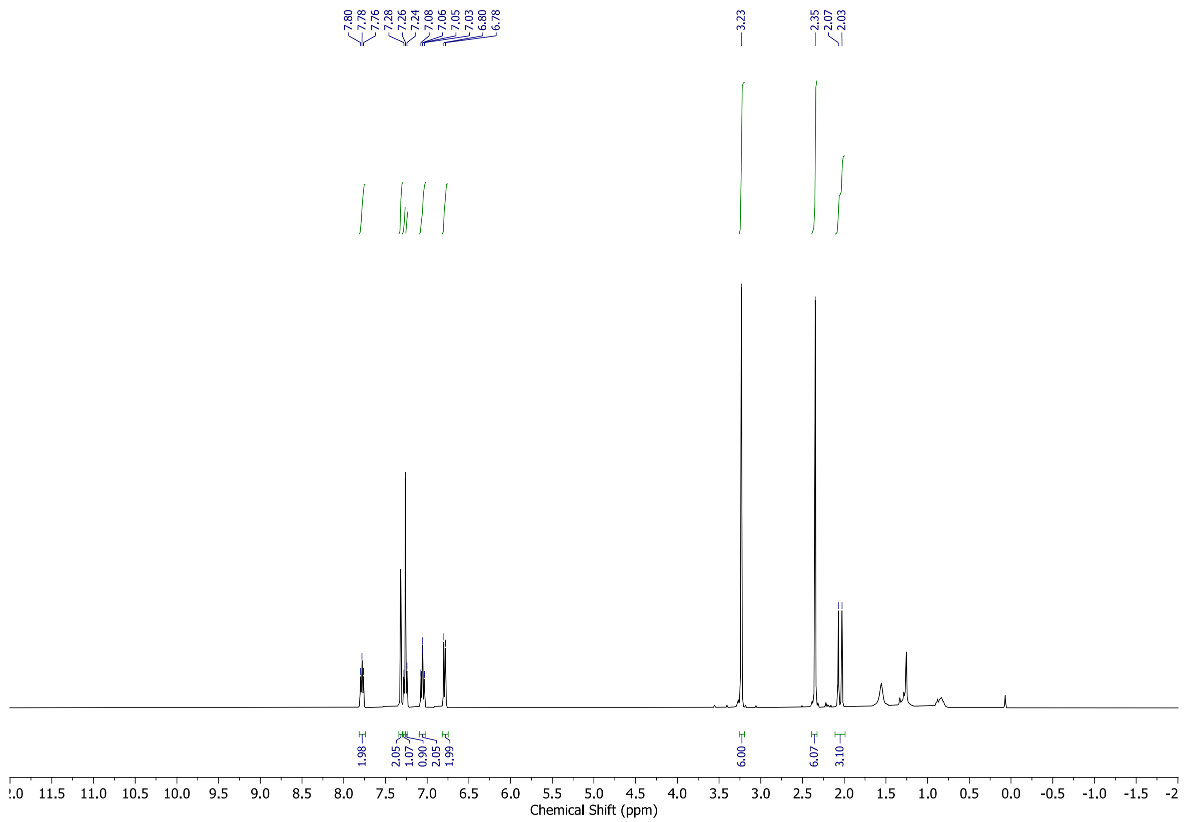

Figure S48. ^{1}H NMR spectrum of (E_SE_S)-**1** ($CDCl_3$, 20 °C).

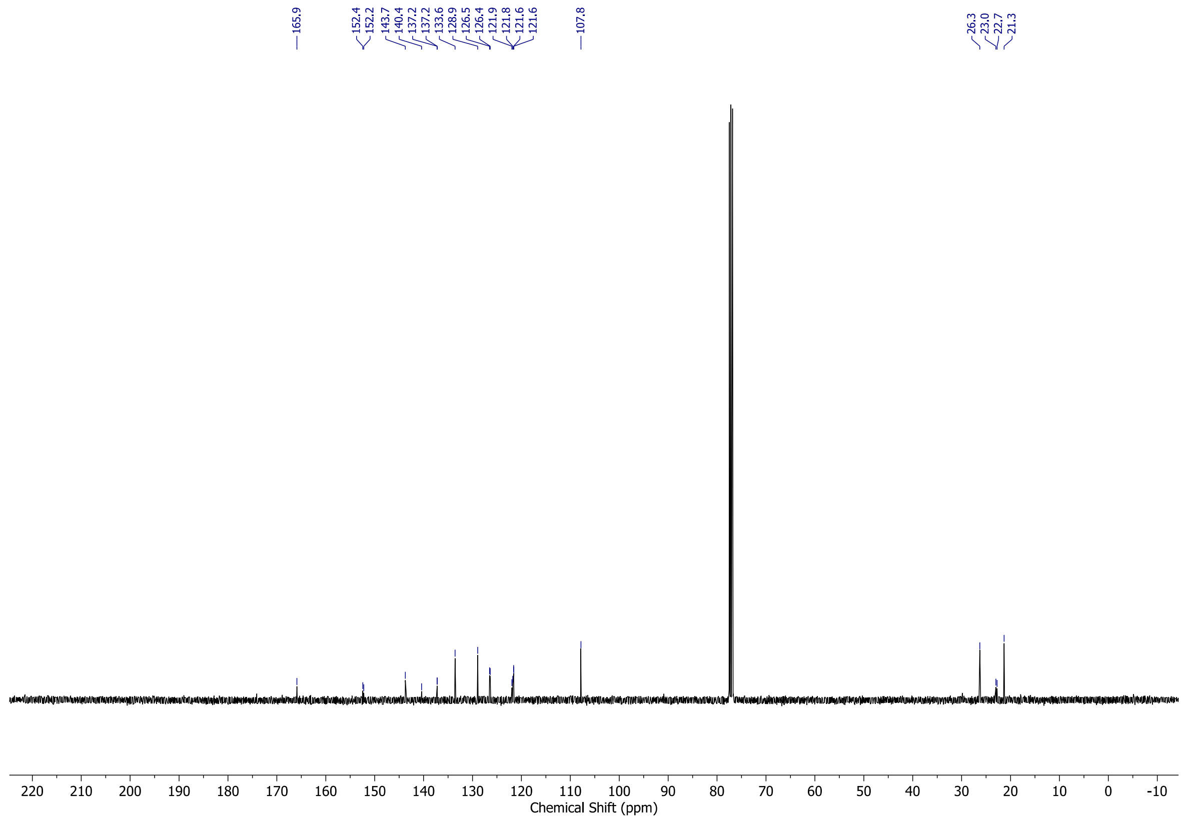

Figure S49. ^{13}C NMR spectrum of (E_SE_S)-**1** ($CDCl_3$, 20 °C).

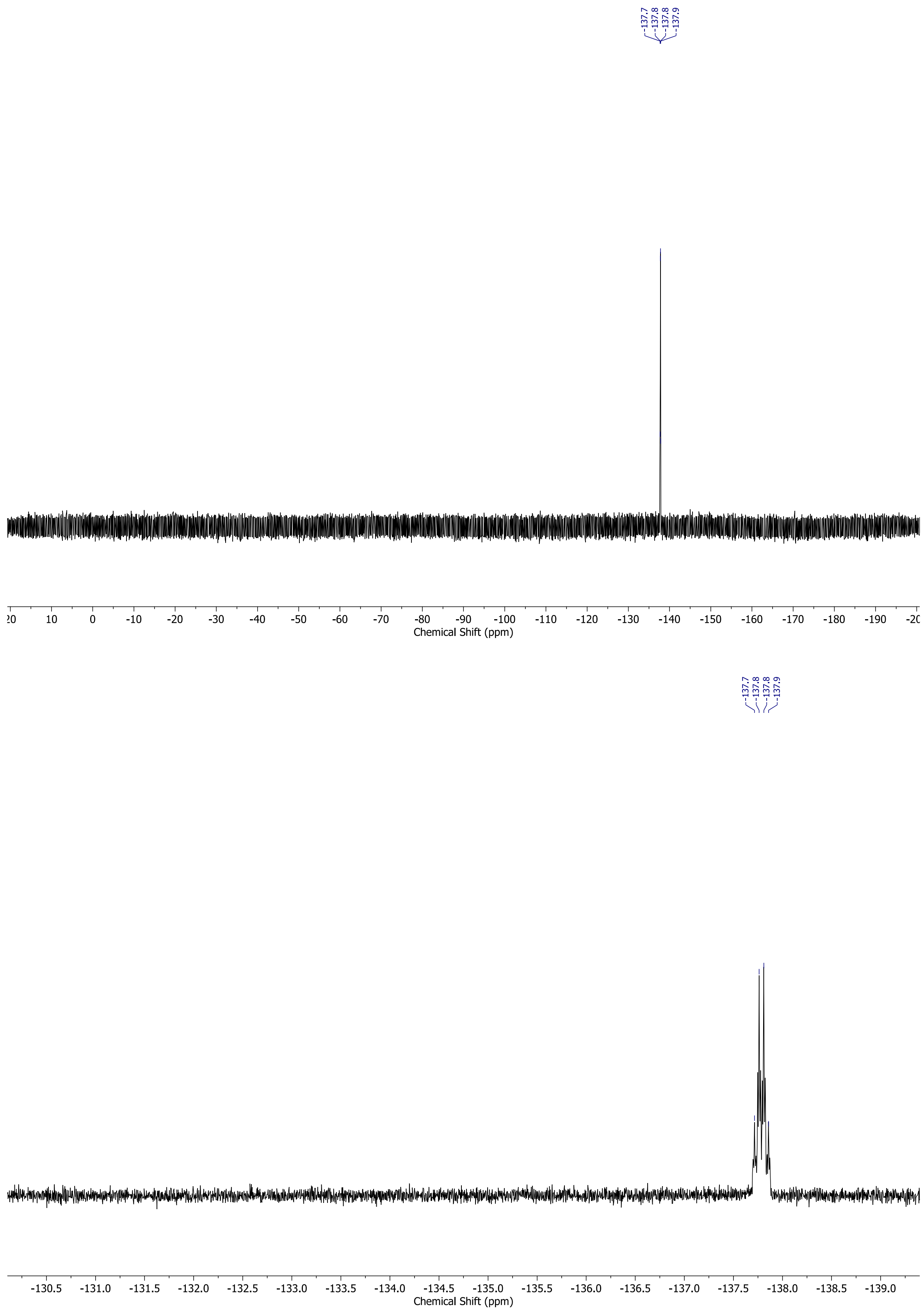

Figure S50. ^{19}F NMR spectrum of (E_SE_S)-**1** ($CDCl_3$, 20 °C, full (top) and zoomed (bottom)).

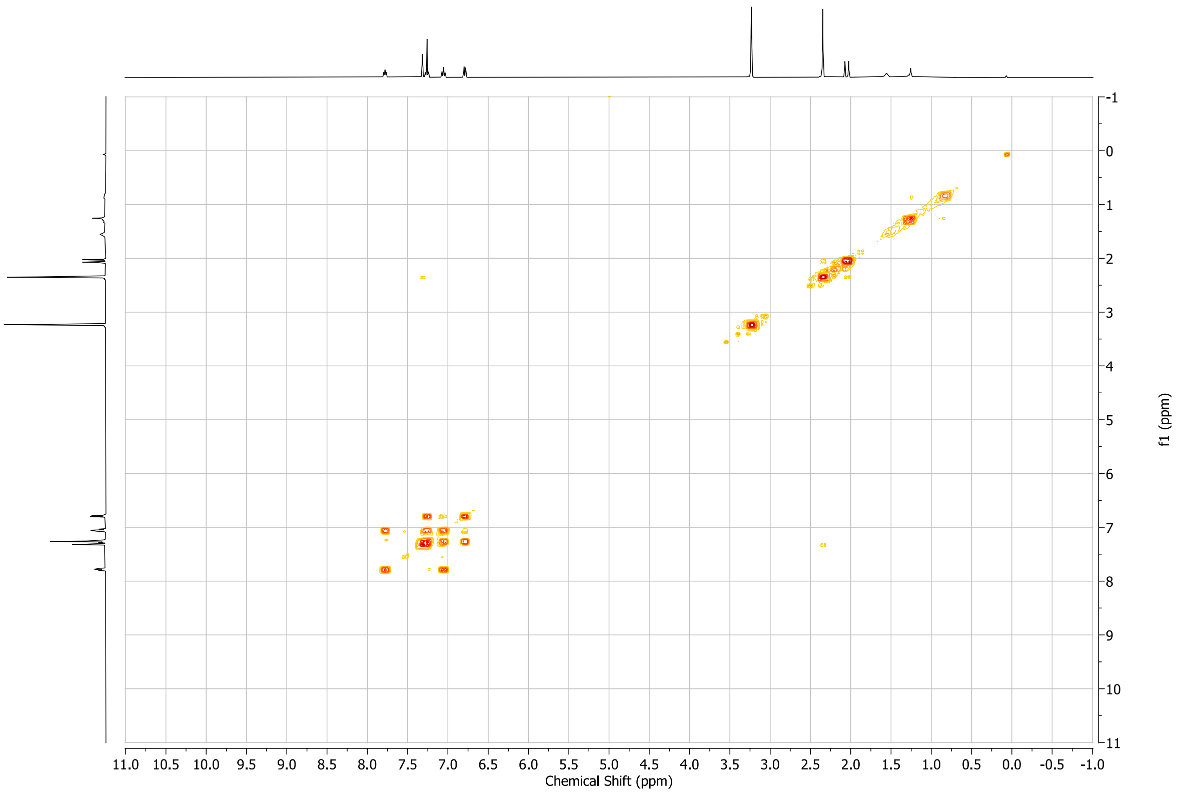

Figure S51. COSY NMR spectrum of (E_SE_S)-**1** ($CDCl_3$, 20 °C).

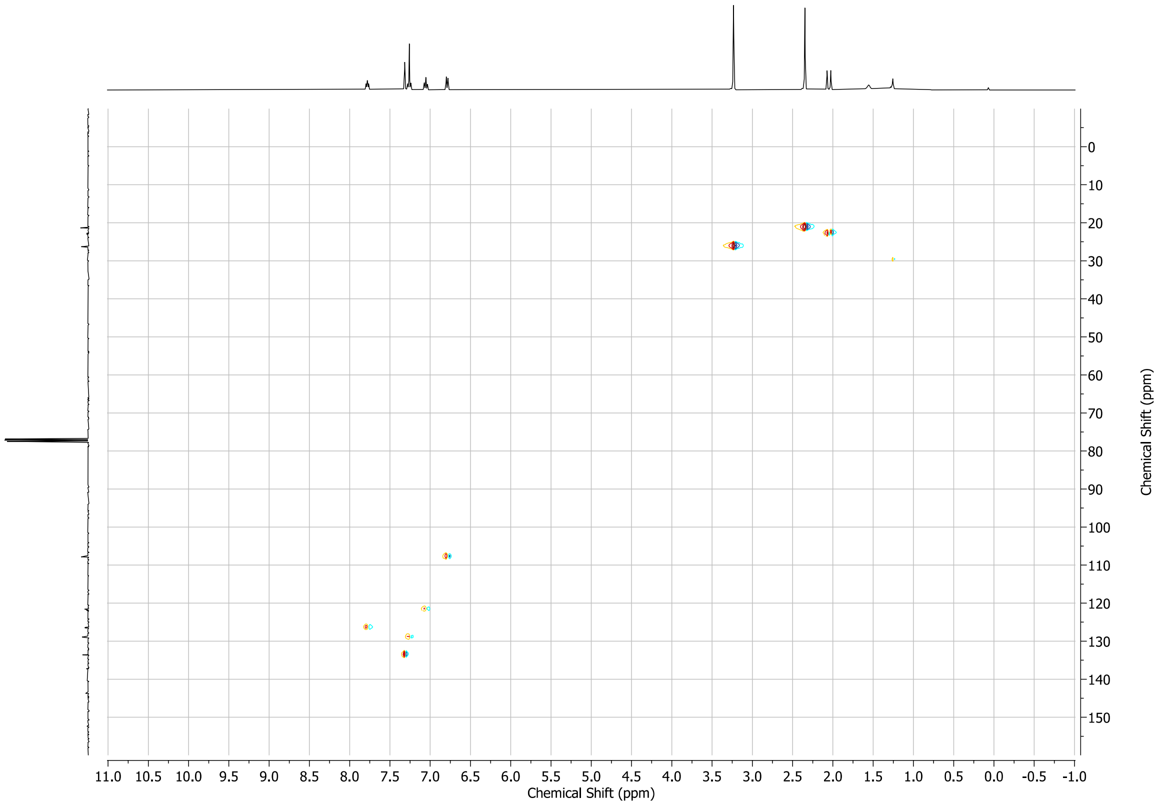

Figure S52. HSQC NMR spectrum of (E_SE_S)-**1** ($CDCl_3$, 20 °C).

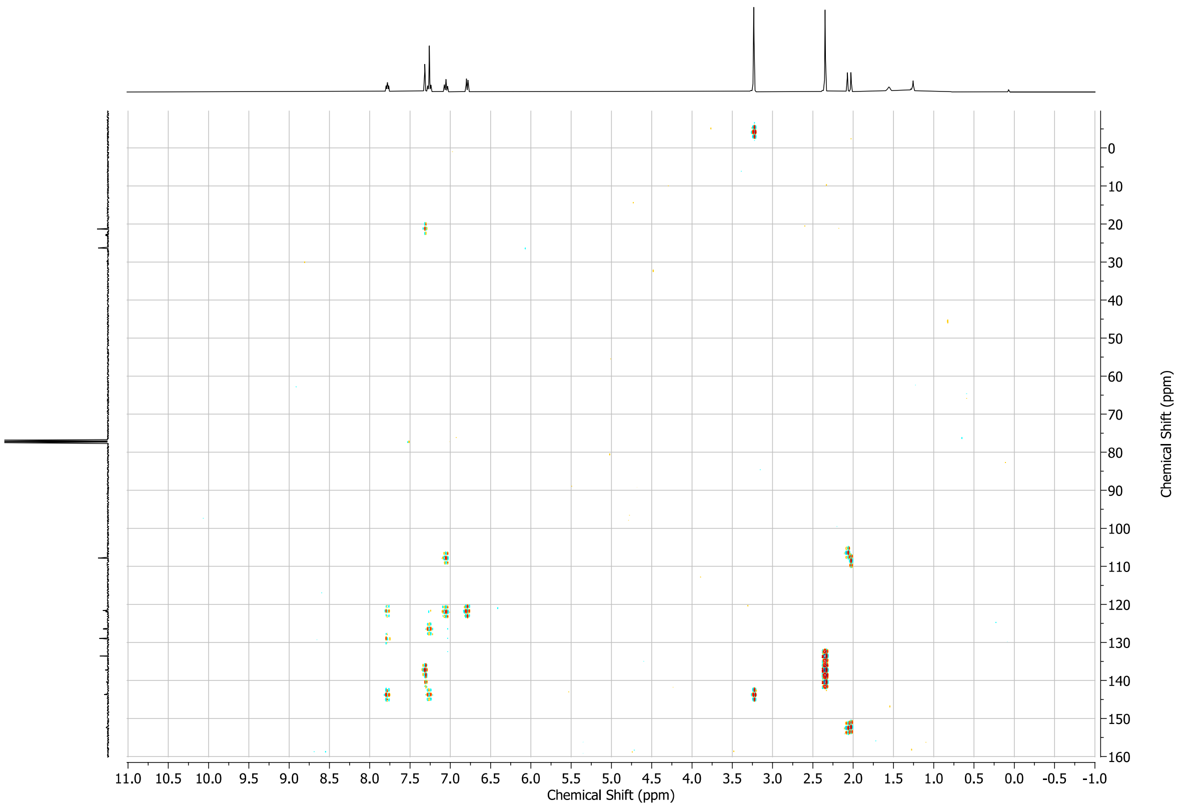

Figure S53. HMBC NMR spectrum of (*E*$_S$*E*$_S$)-**1** ($CDCl_3$, 20 °C).

8. HRMS Spectra

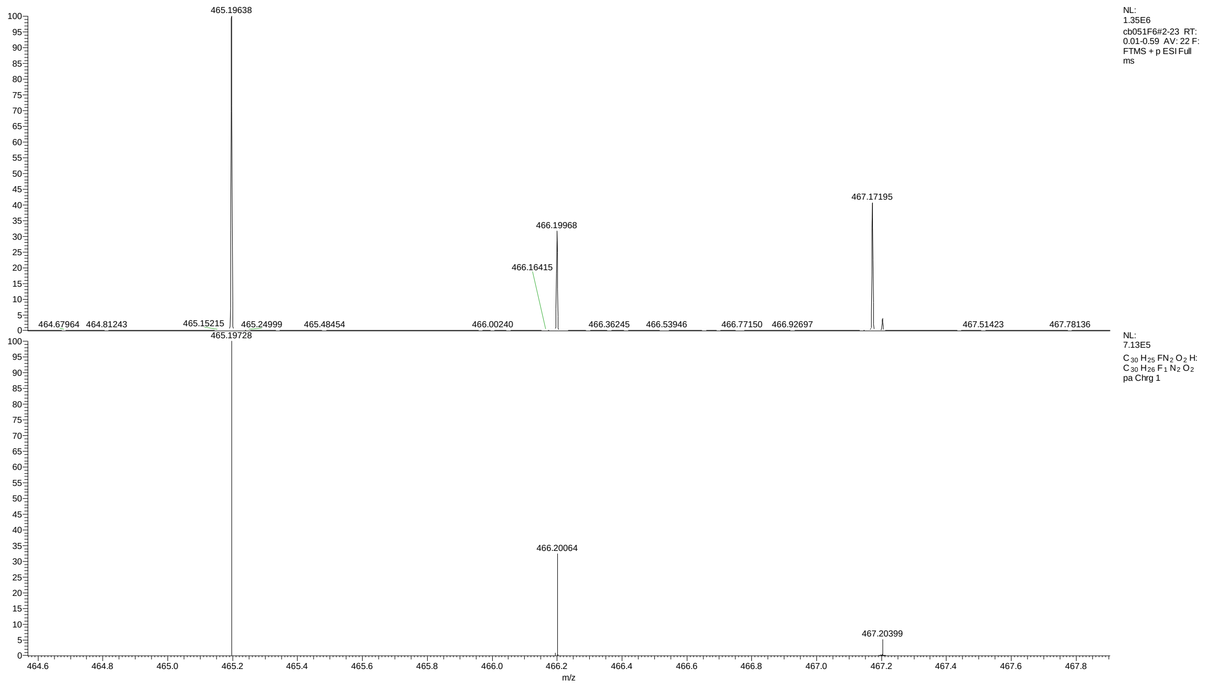

Figure S54. HRMS (ESI$^+$) of (Z_SZ_S)-**1** (top: measured, bottom: calcd.).

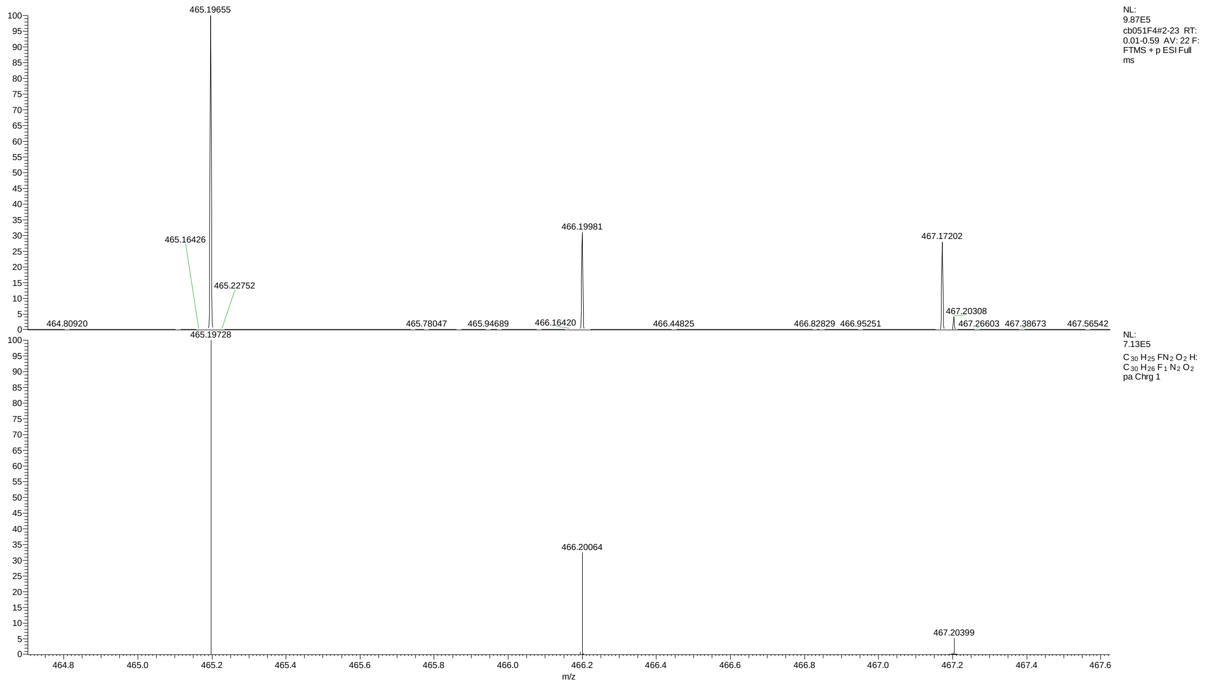

Figure S55. HRMS (ESI$^+$) of (E_SZ_S)-**1** (top: measured, bottom: calcd.).

9. References

1. Kistemaker, J. C. M., Štacko, P., Visser, J. & Feringa, B. L. Unidirectional rotary motion in achiral molecular motors. *Nat. Chem.* **7**, 890–896 (2015).
2. Bergmann, F. T. *et al.* COPASI and its applications in biotechnology. *J. Biotechnol.* **261**, 215–220 (2017).
3. Klok, M., Janssen, L. P. B. M., Browne, W. R. & Feringa, B. L. The influence of viscosity on the functioning of molecular motors. *Faraday Discuss.* **143**, 319 (2009).
4. Sheldrick, G. M. *SHELXT* – Integrated space-group and crystal-structure determination. *Acta Cryst. A* **71**, 3–8 (2015).
5. Sheldrick, G. M. A short history of *SHELX*. *Acta Cryst. A* **64**, 112–122 (2008).
6. Dolomanov, O. V., Bourhis, L. J., Gildea, R. J., Howard, J. A. K. & Puschmann, H. *OLEX2* : a complete structure solution, refinement and analysis program. *J. Appl. Crystallogr.* **42**, 339–341 (2009).
7. Kistemaker, J. C. M. *et al.* Third-Generation Light-Driven Symmetric Molecular Motors. *J. Am. Chem. Soc.* **139**, 9650–9661 (2017).
8. Neese, F., Wennmohs, F., Becker, U. & Riplinger, C. The ORCA quantum chemistry program package. *J. Chem. Phys.* **152**, 224108 (2020).
9. Grimme, S., Hansen, A., Ehlert, S. & Mewes, J.-M. r2SCAN-3c: A “Swiss army knife” composite electronic-structure method. *J. Chem. Phys.* **154**, 064103 (2021).
10. Barone, V. & Cossi, M. Quantum Calculation of Molecular Energies and Energy Gradients in Solution by a Conductor Solvent Model. *J. Phys. Chem. A* **102**, 1995–2001 (1998).
